# Untargeted metabolomics reveals the metabolic characteristics and biomarkers of obstetric antiphospholipid syndrome and undifferentiated connective tissue disease

**DOI:** 10.3389/fmolb.2025.1632244

**Published:** 2025-08-22

**Authors:** Siyin Li, Juan Shi, Xiaofang Shu, Xuemin Jian, Jinmei Zou, Jing Yang

**Affiliations:** ^1^ Department of Rheumatology and Immunology, Mianyang Central Hospital, School of Medicine, University of Electronic Science and Technology of China, Mianyang, Sichuan, China; ^2^ Department of Obstetrics and Gynecology, Mianyang Central Hospital, School of Medicine, University of Electronic Science and Technology of China, Mianyang, Sichuan, China; ^3^ National Health Commission Key Laboratory of Nuclear Technology Medical Transformation, Mianyang Central Hospital, School of Medicine, University of Electronic Science and Technology of China, Mianyang, Sichuan, China

**Keywords:** obstetric antiphospholipid syndrome, undifferentiated connective tissue disease, metabolomics, untargeted, metabolic biomarkers

## Abstract

**Background:**

The clinical differentiation between obstetric antiphospholipid syndrome (OAPS) and undifferentiated connective tissue disease (UCTD) presents significant diagnostic challenges. This study employs metabolomics to investigate metabolic reprogramming patterns in OAPS and UCTD, aiming to identify potential biomarkers for early diagnosis.

**Methods:**

Using LC-MS-based metabolomics, we analyzed serum profiles from 40 OAPS patients (B1), 30 OAPS + UCTD patients (B2), 27 UCTD patients (B3), and 30 healthy controls (A1). Multivariate PLS-DA modeling, combined with KEGG pathway and Gene Set Enrichment Analysis (GSEA), was applied to identify disease-specific metabolic signatures.

**Results:**

Metabolomic profiling detected 1,227 metabolites, including 412 in negative ion mode and 815 in positive ion mode. The two ionization modes exhibited distinct chemical profiles, with PLS-DA analysis demonstrating superior group discrimination in positive ion mode. B1 vs B2 (Negative ion mode): nine metabolites were upregulated (notably 17(S)-HpDHA, showing the largest fold-change as a potential biomarker), and one metabolite was downregulated (5-sulfosalicylic acid). B1 vs B2 (Positive ion mode): 17 metabolites were upregulated (including 4-methyl-5-thiazoleethanol, a promising biomarker), and eight were downregulated. B1 vs B3 (Negative ion mode): 14 metabolites were upregulated (highlighted by 3-hydroxybenzoic acid, the most significantly altered candidate), and four were downregulated. B1 vs B3 (Positive ion mode): 30 metabolites were upregulated (again featuring 4-methyl-5-thiazoleethanol), and 32 were downregulated. B2 vs B3 (Negative ion mode): 15 metabolites were upregulated (e.g., chlortetracycline), and 15 were downregulated (notably 6α-prostaglandin I1). B2 vs B3 (Positive ion mode): 29 metabolites were upregulated (e.g., senecionine), and 64 were downregulated (e.g., SM 9:1 2O/16:4). These metabolites represent robust candidates for group discrimination. Enrichment analysis revealed that distinct metabolic pathways were significantly associated with different groups and ionization modes, suggesting divergent underlying metabolic mechanisms.

**Conclusion:**

This study systematically characterizes the metabolic reprogramming in OAPS, UCTD, and their comorbid states, identifying potential diagnostic biomarkers. Differential metabolites and pathway analyses highlight the critical role of immunity, contributing to a theoretical framework for “metabolism-immunity-vascular” interactions.

## Introduction

Autoimmune connective tissue diseases (CTDs) exhibit significant gender disparities, with an incidence rate approximately 9–10 times higher in women of childbearing age than in men (Marder et al., 2016; Arese et al., 2019). The interaction between these diseases and the reproductive system is bidirectional: (1) CTDs can cause reproductive dysfunction, including infertility and recurrent miscarriage, and (2) immune remodeling during pregnancy may modify disease progression, with the nature and extent of this effect varying by disease subtype (Cervera and Balasch, 2008; Mecacci et al., 2007; Østensen et al., 2015). Among CTDs, obstetric antiphospholipid syndrome (OAPS) and undifferentiated connective tissue disease (UCTD) are of particular interest due to their distinct clinical features.

OAPS is serologically characterized by persistent antiphospholipid antibody positivity, with its core pathology involving dysregulated immune-coagulation crosstalk (Sammaritano, 2020; Valesini and Alessandri, 2005). Clinical studies show that approximately 35% of OAPS patients develop severe pregnancy complications, such as preeclampsia and fetal growth restriction (Ruiz-Irastorza et al., 2010; Ceccarelli et al., 2012). While complement activation and thrombosis are established drivers of disease progression, the metabolic reprogramming underlying these pathological changes remains poorly understood. Current research primarily examines isolated system abnormalities, lacking a comprehensive exploration of the immune-metabolic-coagulation axis (Ceccar et al., 2022).

In contrast, UCTD—a heterogeneous clinical syndrome—lacks standardized diagnostic criteria. Patients often present with “subclinical” manifestations, such as Raynaud’s phenomenon, arthralgia, and nonspecific autoantibody positivity, leading to misdiagnosis as other rheumatic diseases in approximately 40% of early-stage cases (Hysa et al., 2023; Marwa and Anjum, 2025; Antunes et al., 2019). This diagnostic uncertainty not only delays treatment but also complicates molecular pathogenesis research. Thus, identifying objective biomarkers to distinguish UCTD from other CTDs is critical unmet clinical need.

Advances in multi-omics technologies have highlighted the unique value of, metabolomics, given its “phenotype-proximal” nature. Unlike the static data from genomics or transcriptomics, the metabolome dynamically captures the body’s real-time response to pathological stimuli (Skirycz and Dickinson, 2023). Untargeted metabolomics, in particular, enables unbiased detection of thousands of metabolites and has been successfully applied to classic CTDs like systemic lupus erythematosus, revealing disruptions in tryptophan metabolism and fatty acid oxidation (Ferreira et al., 2019; Fernández-Ochoa et al., 2019; Bengtsson et al., 2016; Li J. et al., 2018). However, research on OAPS and UCTD—especially regarding metabolic profiles in comorbid cases—remains limited.

To address this gap, our study employs high-resolution mass spectrometry to conduct the first systematic metabolomic analysis of three characteristic populations (OAPS patients, UCTD patients, and OAPS–UCTD comorbid patients). Our objectives are to: (1) define disease-associated metabolic signatures; (2) uncover pathogenic metabolic pathways, and (3) identify potential biomarkers for accurate differentiation among OAPS, UCTD, and healthy controls.

## Materials and methods

### Study design and patients

This study enrolled 127 female participants divided into four groups: (1) B1 (n = 40): OAPS patients meeting both revised Sydney International Antiphospholipid Syndrome (APS) Classification Criteria (2006) and the definition of non-standard OAPS from the “Expert Consensus on the Diagnosis and Management of Obstetric Antiphospholipid Syndrome”; (2) B2 (n = 30): OAPS + UCTD comorbidity patients; (3) B3 (n = 27): UCTD-only patients; and (4) A1 (n = 30): healthy controls. UCTD diagnosis required: ≥1 CTD-related clinical manifestation; serological evidence of autoimmunity; failure to meet classification criteria for defined CTDs (Mosca et al., 2008). OAPS diagnostic criteria included: Revised Sydney APS Classification Criteria (2006) (Miyakis et al., 2006); Non-standard OAPS per expert consensus, encompassing: Atypical laboratory findings with typical clinical manifestations (e.g., aPL positive twice within <12 weeks; aCL/β2GPI titers 20–39 GPL/MPL), or Atypical clinical presentations with laboratory-confirmed APS (e.g., ≥2 unexplained miscarriages, late-onset preeclampsia). Participant ages were well-matched across groups (30.15–32.78 years), with detailed characteristics presented in Table 1.

**TABLE 1:** The baseline characteristics of included patients.

Variables	OAPS (n = 40)	OAPS complicated by UCTD (n = 30)	UCTD (n = 27)	Healthy control (n = 30)
Age (years)	31.175 ± 4.587	31.379 ± 4.986	32.778 ± 3.975	29.920 ± 3.346
Number of miscarriages	2.200 ± 1.054	1.185 ± 1.055	1.389 ± 0.678	-
Abortion within 10 weeks	1.275 ± 1.323	0.667 ± 1.164	1.308 ± 0.722	-
Abortion after 10 weeks	0.925 ± 0.721	0.500 ± 0.719	0.148 ± 0.355	-
Eclampsia	1 (2.5%)	0 (0.0%)	0 (0.0%)	-
Intrauterine growth retardation	1 (2.5%)	1 (3.3%)	1 (3.7%)	-
Fluid accumulation around the gestational sac	3 (7.5%)	1 (3.3%)	3 (11.1%)	-
premature birth	4 (10.0%)	4 (13.3%)	0 (0.0%)	-

### Sample collection and metabolomic profiling

Morning fasting blood samples (5 mL) were collected from all participants using standardized protocols. Following centrifugation at 3,000 rpm for 10 min, serum aliquots were stored at −80 °C until analysis. For LC-MS analysis, samples underwent methanol-based protein precipitation followed by dual centrifugation (15,000 rpm, 20 min at 4 °C) to ensure optimal metabolite recovery. Quality control measures included preparation of pooled QC samples and methanol blanks to monitor system stability and background interference throughout the analytical batches.

### Metabolite identification and data processing

High-resolution mass spectrometry data were processed separately for positive and negative ionization modes using rigorous quality filters, including CV <30% in QC samples and retention time alignment (CV <10%). Metabolite identification followed a tiered confidence approach: Level 1 identifications (12%) used authentic standards; Level 2 (68%) relied on GNPS library matching (cosine similarity >0.8); and Level 3 (20%) employed accurate mass matching (<5 ppm) against HMDB/METLIN/LipidMaps. Missing values were imputed using a k-nearest neighbors algorithm (k = 10% group size) with Euclidean distance metrics to preserve biological patterns.

### Differential metabolite screening and classification

Samples were divided into three comparison groups corresponding to patients with OAPS, patients with OAPS complicated by UCTD, and patients with UCTD. Multivariate statistical analyses were then conducted, beginning with unsupervised principal component analysis (PCA). PCA was used to summarize and reduce the dimensionality of sample features without considering group labels. It enabled visualization of overall sample distribution, identification of clustering or dispersion trends, and assessment of analytical stability, including detection of outliers or unstable factors that could impact further analysis.

Following PCA, partial least squares discriminant analysis (PLS-DA) was performed on the full sample set using the Scikit-learn library in Python 3.5.0. For PLS-DA modeling, 5-fold cross-validation was implemented to optimize model parameters (number of latent variables), with performance evaluated via the Q2 metric to avoid overfitting. Given the moderate sample sizes across groups (minimum n = 27 in B3) and minimal class imbalance (maximum ratio 1.5:1 between groups), data balancing techniques (e.g., SMOTE) were not required, as validated by stable cross-validation performance (SD of Q2 < 0.05 across folds). A PLS-DA scatter plot was subsequently generated to visually display the separation of samples across groups, illustrating their positions within feature space and further clarifying the differences between groups.

### Pathway enrichment and gene set enrichment analysis

To further explore the biological significance of the identified differential metabolites, a series of analyses was conducted. First, the differential metabolites were annotated using the Kyoto Encyclopedia of Genes and Genomes (KEGG) database. Based on this annotation, pathway enrichment analysis was performed to identify the metabolic pathways in which these differential metabolites were significantly enriched and to clarify the biological processes they may be involved in.

After identifying the key metabolic pathways, the decision tree method was used to screen for differential metabolites involved in these pathways from the complete set of differential metabolites. These analyses—including pathway enrichment and decision tree screening—were conducted using R software and relevant packages, specifically clusterProfiler, org.Hs.e.g.,.db, enrichplot, and ggplot2.

To further elucidate the functional dynamics and pathway alterations across sample groups, Gene Set Enrichment Analysis (GSEA) was performed. GSEA identifies functionally related gene sets and associated pathway changes. Through quantitative scoring and visual representation, it highlights active biological processes and pathways across different risk stratifications, offering a broader understanding of biological differences among samples from a macroscopic perspective.

### Statistical analysis

All statistical analyses were performed using version-controlled computational environments. Python 3.5.0 was used with SciPy v1.2.1 for hypothesis testing and scikit-learn v0.19.1 for data preprocessing. R 3.4.3 was used for pathway analysis (clusterProfiler v3.6.0), gene annotation (org.Hs.e.g.,.db v3.5.0), visualization (enrichplot v1.6.0), and graphics (ggplot2 v2.2.1). Gene Set Enrichment Analysis was conducted using GSEA v3.0 (Broad Institute).

Differential metabolites were identified using the following thresholds: (1) fold change (FC) ≥ 1.2 or ≤0.83 (equivalent to log2FC ≥ 0.263 or ≤ −0.263) to ensure biologically meaningful changes; (2) false discovery rate (FDR)-adjusted p-value <0.05 (Benjamini–Hochberg method) for statistical significance. Data normality was assessed using the Shapiro-Wilk test (α = 0.05) with Bonferroni correction applied for multiple comparisons. For features meeting normality and homoscedasticity assumptions (Levene’s test, p > 0.05), Hotelling’s T^2^ test was used to assess multivariate group differences. For features violating these assumptions, the Mann-Whitney U test with continuity correction was applied, and the Benjamini–Hochberg false discovery rate (FDR) method was used for multiple testing correction (q < 0.05).

## Results

### Metabolite identification results

Following rigorous quality control procedures, a total of 1,227 metabolites were reliably identified across both ionization modes. Ionization polarity significantly influenced metabolite detection: 412 metabolites were detected in negative ion mode (NEG), and 815 in positive ion mode (POS).

Superclass characterization revealed distinct chemical profiles.

**FIGURE 1:**
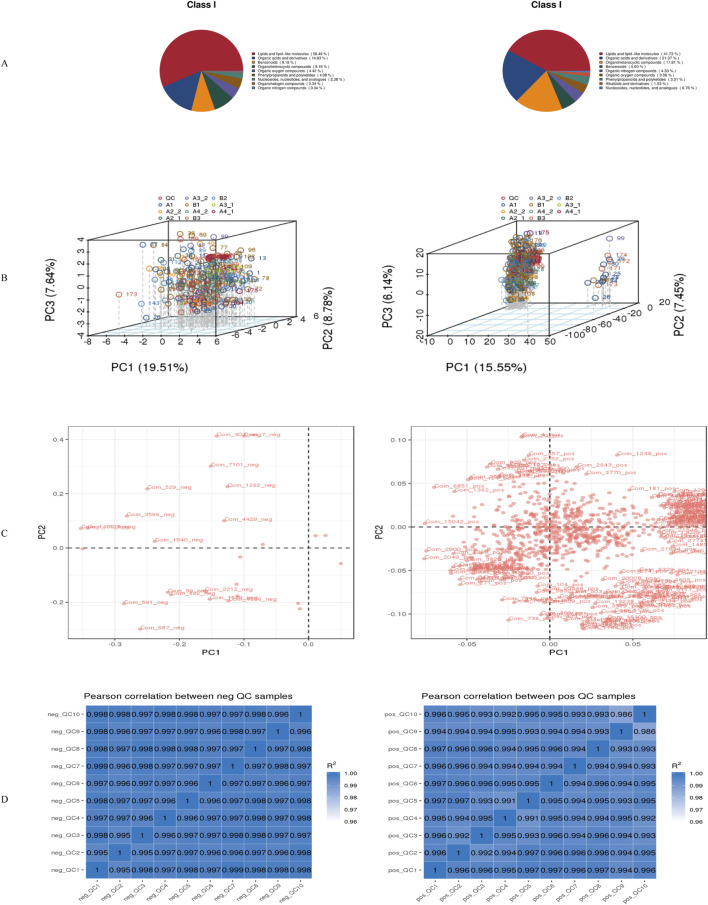
Metabolite identification results. **(A)**Pie chart showing the primary classification of metabolites; **(B)**Total PCA plot of QC and all metabolic samples; **(C)**Loading plot corresponding to the total PCA plot of QC and all metabolic samples; **(D)**Correlation analysis results of QC samples.*Left panels represent negative ion mode; right panels represent positive ion mode. neg: negative ion mode; pos: positive ion mode.

• NEG mode: Predominantly lipids and lipid-like molecules (56.46%), organic acids and derivatives (14.63%), and benzenoids (9.18%), with additional representation from organohalogen compounds (8.16%) and phenylpropanoids/polyketides (4.08%).
• POS mode: Enriched in lipids and lipid-like molecules (41.73%), organic acids and derivatives (21.37%), and organohalogen compounds (17.81%), with further representation from benzenoids (5.60%) and organic nitrogen compounds (4.33%) (Figure 1A).

Multivariate analysis revealed ionization-dependent metabolic variation.

• NEG mode: PC1 = 19.51%, PC3 = 7.64%
• POS mode: PC1 = 15.55%, PC3 = 6.14% (Figure 1B)

Key mass features contributing to PC1–PC2 separation exhibited strong correlations (|r| > 0.7) within superclass clusters, particularly lipid species in NEG mode and alkaloids in POS mode (Figure 1C). Cross-modal correlation analysis also demonstrated significant inter-mode metabolite associations (Figure 1D).

### Metabolite annotation

**FIGURE 2:**
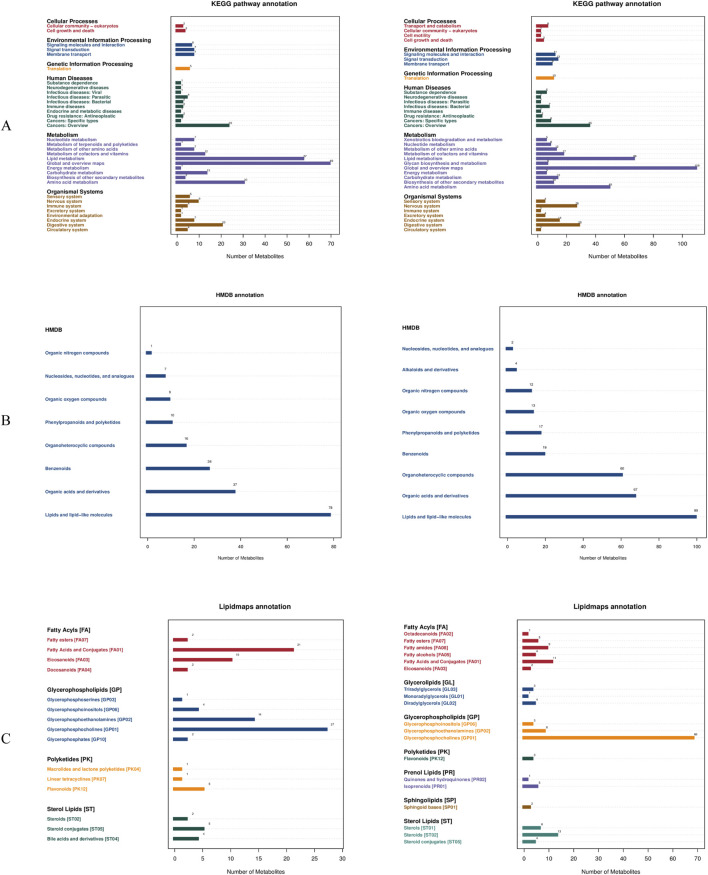
Metabolite annotation results. **(A)** Statistical chart of KEGG annotation results; **(B)** Statistical chart of HMDB classification annotation results; **(C)** Statistical chart of LipidMaps classification annotation results. *Left panels represent negative ion mode; right panels represent positive ion mode.

Metabolite annotation was conducted using three complementary classification systems, with database-specific biological insights illustrated in Figure 2.

• KEGG annotation categorized metabolites into functional groups, including:
o Core metabolic processes: Metabolism
o Regulatory systems: Genetic information processing, Environmental information processing
o Higher-order functions: Cellular processes, Organismal systems
o Pathological associations: Human diseases (Figure 2A)
• HMDB classification revealed chemical superclass distributions, identifying:
oDominant classes: Lipids and lipid-like molecules, organic acids and derivatives, organoheterocyclic compounds
oSpecialized metabolites: Benzenoids
oFunctional molecules: Nucleoside/nucleotide analogues, organic nitrogen compounds (Figure 2B)

Ionization mode-dependent lipid profiling included primarily fatty acyls, glycerophospholipids, and sterol lipids (Figure 2C).

### Differential metabolite screening results

The PLS-DA score plots systematically compare inter-group metabolic disparities across three contrasts (B1 vs. B2; B1 vs. B3; B2 vs. B3) in both ionization modes. This approach provides a comprehensive overview of how metabolic profiles differ among the groups.

**FIGURE 3:**
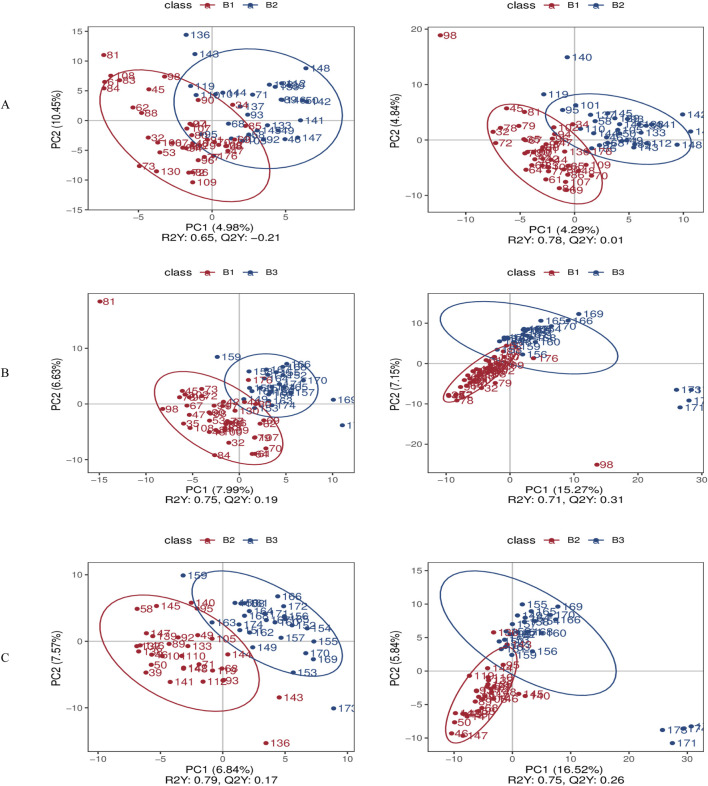
Differential metabolite screening results. **(A)** PLS-DA score comparison between Group B1 and Group B2; **(B)** PLS-DA score comparison between Group B1 and Group B3; **(C)** PLS-DA score comparison between Group B2 and Group B3. *Left panels represent negative ion mode; right panels represent positive ion mode.

In the negative ionization mode, PC1 accounted for 4.98%–7.99% of the variance (with a permutation p-value <0.01), and PC2 accounted for 6.63%–10.45%. In positive ion mode, the permutation p-values for these comparisons were also <0.01, confirming the reliability of group separation in both ionization modes. PC1 explained 4.29%–16.52% of the variance, while PC2 accounted for 4.84%–7.15%. Notably, the positive ionization mode exhibited superior group separation, as evidenced by the Q^2^ values (Q^2^ = 0.01–0.31 in the positive mode compared to −0.21–0.19 in the negative mode) (Figure 3). This indicates that the positive mode is more effective in distinguishing metabolic differences between the groups.

**FIGURE 4:**
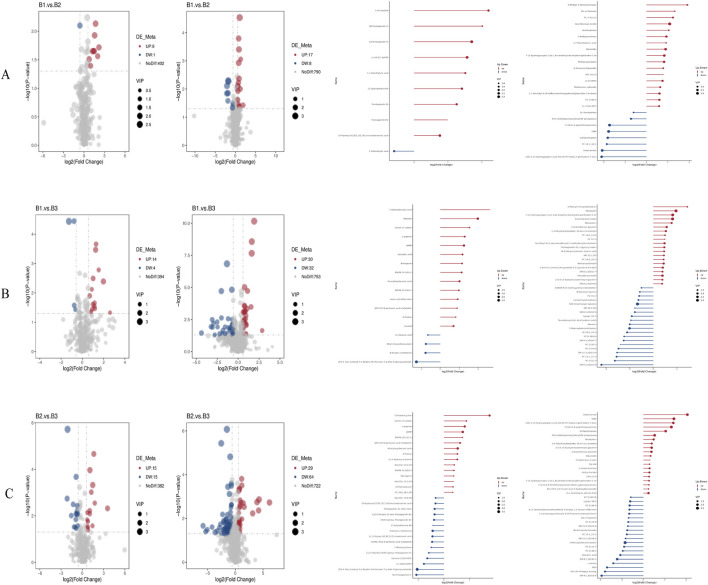
Volcano plots and bar charts of differential metabolites. **(A)** Volcano and bar charts for the comparison between Group B1 and Group B2; **(B)** Volcano and bar charts for the comparison between Group B1 and Group B3; **(C)** Volcano and bar charts for the comparison between Group B2 and Group B3. *Left panels represent negative ion mode; right panels represent positive ion mode.

A volcano plot was used to visually represent the distribution of differentially expressed metabolites when comparing groups under both negative and positive ion modes (Figure 4 and Supplementary File 1).

1. Comparison of B1 and B2:
oNegative ion mode: nine metabolites were upregulated (log2FC ≥ 0.263, FDR <0.05) and one was downregulated (log2FC ≤ −0.263, FDR <0.05). Among them, 17(S)-HpDHA with a fold change of 4.57, showing the largest fold-change as a potential biomarker, suggesting its potential as a biomarker for distinguishing between B1 and B2, and one metabolite was downregulated (5-sulfosalicylic acid).
oPositive ion mode: 17 metabolites met the upregulation criteria (e.g., 4-methyl-5-thiazoleethanol, log2FC = 0.62, FDR = 0.012) and eight met the downregulation criteria.
2. Comparison of B1 and B3:
oNegative ion mode: 14 upregulated metabolites (e.g., 3-hydroxybenzoic acid, log2FC = 0.58, FDR = 0.008) and four downregulated metabolites (log2FC ≤ −0.263, FDR <0.05) were identified.
oPositive ion mode: 30 upregulated (including 4-methyl-5-thiazoleethanol, log2FC = 0.71, FDR = 0.005) and 32 downregulated metabolites passed the filtering criteria.
3. Comparison of B2 and B3:
oNegative ion mode: 15 upregulated (e.g., chlortetracycline, log2FC = 0.49, FDR = 0.015) and 15 downregulated (e.g., 6α-prostaglandin I1, log2FC = −0.32, FDR = 0.021) metabolites were detected.
oPositive ion mode: 29 upregulated (e.g., senecionine, log2FC = 0.55, FDR = 0.009) and 64 downregulated (e.g., SM 9:1 2O/16:4, log2FC = −0.41, FDR = 0.018) metabolites met the thresholds.

**FIGURE 5:**
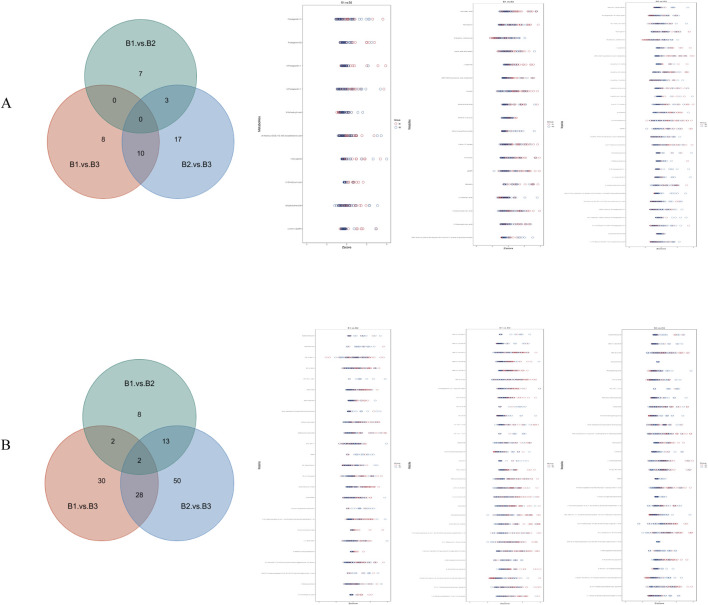
Venn diagram and Z-score diagram of differential metabolites. **(A)** Results in negative ion mode; **(B)** Results in positive ion mode.

Further analysis was conducted to identify overlapping metabolites between the positive and negative ion modes across all three group comparisons. The results are presented in Figure 5. In the negative ion mode, there were 10 differential metabolites between B1 and B2, 18 between B1 and B3, and 30 between B2 and B3. In the positive ion mode, there were 25 between B1 and B2, 62 between B1 and B3, and 93 between B2 and B3.

Finally, Supplementary File 2 provides a comprehensive analysis of the diagnostic capabilities of differentially expressed metabolites across all group comparisons, including ROC curve analyses and key metrics. Notably, several metabolites exhibited robust and consistent discriminative power across both ionization modes, with AUC values >0.8 in at least one group comparison, reinforcing their potential as reliable biomarkers. These include: (1) In the B1 vs. B3 comparison: 3-hydroxybenzoic acid (Negative Ion Mode), demonstrated strong discriminative ability with an AUC of 0.829. As highlighted earlier, this metabolite is a key candidate biomarker in negative ion mode for distinguishing B1 from B3 and is involved in pathways related to oxidative stress and inflammation, which are central to OAPS pathogenesis; 4-methyl-5-thiazoleethanol (Positive Ion Mode): exhibited a high AUC of 0.898, consistent with its repeated identification as a prominent differential metabolite in positive ion mode for B1 vs. B3. Its upregulation aligns with enriched pathways such as choline metabolism, linking it to immune-metabolic crosstalk in OAPS; Additional positive ion mode metabolites with notable AUC values for B1 vs. B3 include senecionine (AUC: 0.857) and a sphingolipid derivative (SM 9:1 2O/16:4, AUC: 0.839), both of which map to lipid metabolism pathways (sphingolipid metabolism and linoleic acid metabolism, respectively) that are significantly enriched in this comparison. (2) In the B2 vs. B3 comparison: Chlortetracycline (Negative Ion Mode): showed strong discriminative power with an AUC of 0.888, consistent with its marked upregulation in B2 vs. B3 and its association with perturbed microbial-immune interactions in comorbid states; 6α-prostaglandin I1 (Negative Ion Mode): exhibited an AUC of 0.810, aligning with its consistent downregulation in B2 vs. B3 and its role in vascular smooth muscle contraction pathways, which are dysregulated in the comorbid state; In positive ion mode, SM 9:1 2O/16:4 (AUC: 0.847) and 5,557 (a linoleic acid derivative, AUC: 0.803) further supported the relevance of sphingolipid and polyunsaturated fatty acid metabolism in distinguishing B2 from B3. These metabolites, by virtue of their cross-modal consistency and association with disease-specific pathways, form a cohesive biomarker panel that enhances the reliability of distinguishing OAPS, UCTD, and their comorbid state.

### Metabolic pathway enrichment analysis

**FIGURE 6:**
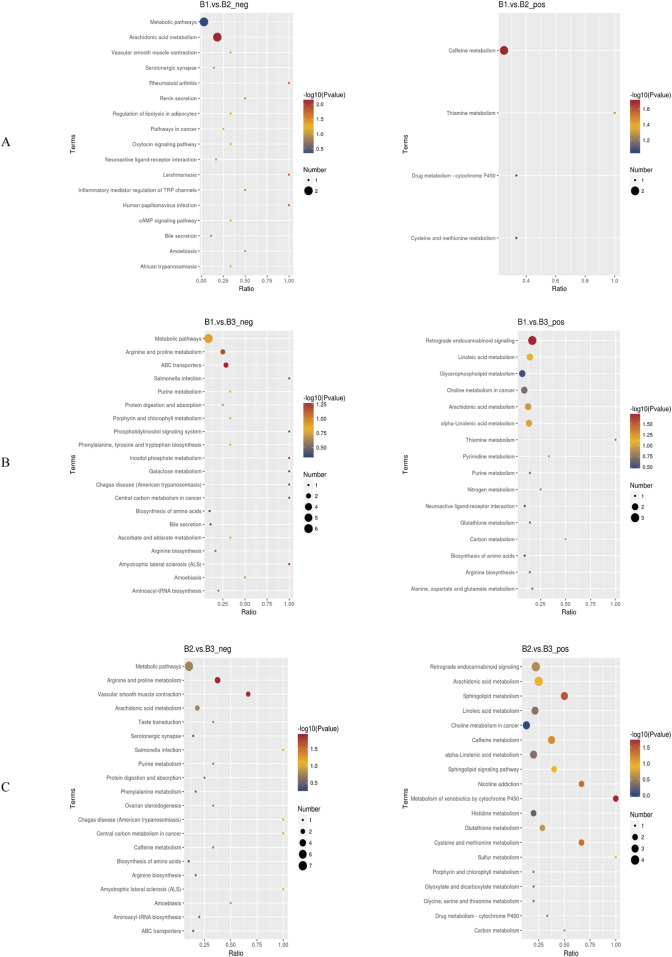
Bubble charts of KEGG pathway enrichment. **(A)** Bubble chart of KEGG enrichment for the comparison between Group B1 and Group B2; **(B)** Bubble chart of KEGG enrichment for the comparison between Group B1 and Group B3; **(C)** Bubble chart of KEGG enrichment for the comparison between Group B2 and Group B3. *Left panels represent negative ion mode; right panels represent positive ion mode. neg: negative ion mode; pos: positive ion mode.

Enrichment analysis was used to identify significantly enriched pathways across different groups under both negative and positive ion modes (Figure 6). Specifically.

1. B1 vs. B2 comparison:
oNegative ion mode: Pathways such as general metabolic pathways and arachidonic acid metabolism were significantly enriched.
oPositive ion mode: The caffeine metabolism pathway was significantly enriched (Figure 6A).
2. B1 vs. B3 comparison:
oNegative ion mode: Significantly enriched pathways included metabolic pathways, arginine and proline metabolism, and ABC transporters.
oPositive ion mode: Pathways such as retrograde endocannabinoid signaling, linoleic acid metabolism, glycerophospholipid metabolism, choline metabolism in cancer, arachidonic acid metabolism, and alpha-linolenic acid metabolism were enriched (Figure 6B).
3. B2 vs. B3 comparison:
oNegative ion mode: Enriched pathways included metabolic pathways, arginine and proline metabolism, and vascular smooth muscle contraction.
oPositive ion mode: Enriched pathways included retrograde endocannabinoid signaling, arachidonic acid metabolism, sphingolipid metabolism, linoleic acid metabolism, choline metabolism in cancer, and alpha-linolenic acid metabolism (Figure 6C).

Notably, the metabolic pathway was enriched across all three group comparisons under the negative ion mode, suggesting that it may serve a foundational or universal role in the metabolic processes of these conditions. Additionally, retrograde endocannabinoid signaling, linoleic acid metabolism, and choline metabolism in cancer were enriched in both the B1–B3 and B2–B3 comparisons. This implies that these pathways may be critically involved in the physiological or pathological differences represented by these groups.

The results of the GSEA are available in Supplementary File 3, including glycerophospholipid_metabolism, metabolic_pathways, choline_metabolism_in_ cancer, steroid_hormone_biosynthesis, arachidonic_acid_metabolism, alpha-linolenic _acid_metabolism, and retrograde_endocannabinoid_signaling. With respect to enrichment changes in functionally related metabolic pathways across disease backgrounds, the findings suggest that no single metabolic pathway exhibited dominant activity or consistent alteration during disease progression.

## Discussion

This study employed untargeted metabolomics coupled with PLS-DA modeling to systematically characterize metabolic alterations in OAPS, UCTD, and their comorbid state. Our multi-modal approach identified distinct metabolic signatures and dysregulated pathways that differentiate these clinical conditions, overcoming limitations of current diagnostic methods that rely on single-modal biomarkers or clinical criteria alone (Mosca et al., 2008; Miyakis et al., 2006). Notably, our model demonstrated particular value in distinguishing the clinically challenging OAPS + UCTD comorbidity subgroup (B2), where conventional diagnostic criteria often lack specificity.

It is worth noting that previous large-scale metabolomics studies on seven systemic autoimmune diseases have confirmed that metabolite profiles involving unsaturated fatty acids, acylglycines, acylcarnitines, and amino acids possess strong disease-discriminating power (Fernández-Ochoa et al., 2020). However, in the field of female-specific immune disorders—particularly in studies investigating metabolic regulatory mechanisms and biomarkers of OAPS, UCTD, and their comorbid states—significant research gaps remain. The innovative value of this study lies in its multi-dimensional metabolomics analysis, which systematically elucidated the specific metabolic reprogramming patterns of the three disease groups. The identified differential metabolites and their associated pathways not only offer a new perspective for understanding the comorbidity mechanisms of OAPS and UCTD but also demonstrate translational potential as auxiliary diagnostic markers, providing a theoretical basis for developing novel diagnostic biomarkers.

This study revealed metabolic differences between patients with OAPS and those with OAPS complicated by UCTD. In the negative ion mode, nine metabolites were significantly upregulated and one was downregulated. These differential metabolites were mainly enriched in the general metabolic pathway and the arachidonic acid metabolism pathway. Mechanistic analysis suggests that activation of the arachidonic acid metabolism pathway may result from a cascade reaction triggered by immune dysregulation in the comorbid UCTD state (Zhu et al., 2024). Specifically, increased activities of phospholipase A2 (PLA2) and cyclooxygenase (COX) could promote the rapid conversion of arachidonic acid—released from membrane phospholipids—into prostaglandin H2 precursors, which are subsequently converted into various prostaglandin subtypes by downstream isomerases (Collu et al., 2020). The observed downregulation of 5-sulfosalicylic acid indicates possible compensatory changes in secondary metabolic pathways. Resource diversion within the main metabolic pathway may hinder the synthesis or breakdown of this metabolite via substrate competition or metabolic feedback mechanisms.

In the positive ion mode, 17 metabolites were upregulated and eight were downregulated, with the caffeine metabolism pathway showing the most significant enrichment. The increased activity of CYP1A2, a key enzyme in the N-demethylation of caffeine, may accelerate the conversion of caffeine into metabolites such as 1,7-dimethylxanthine and 1-methylxanthine. This phenomenon may be linked to the chronic inflammatory state of patients with comorbid conditions, as inflammatory cytokines are known to regulate cytochrome P450 enzyme system expression (Lang and Rettie, 2000). These findings suggest a characteristic metabolic reprogramming pattern in patients with OAPS complicated by UCTD: against a background of persistent immune-inflammatory axis activation, the body intensifies the inflammatory response via the arachidonic acid–prostaglandin cascade, while abnormal activation of the caffeine metabolism pathway may reflect a compensatory upregulation of hepatic detoxification functions.

The significantly upregulated or downregulated metabolites identified in the comparison between OAPS and OAPS + UCTD may also have relevance as biomarkers in other immune-mediated diseases. For instance, 17(S)-HpDHA—significantly upregulated in the negative ion mode in patients with OAPS + UCTD compared to those with OAPS—has been associated with inflammatory responses in previous studies (Butovich et al., 2006). In autoimmune diseases such as rheumatoid arthritis, similar metabolites within the arachidonic acid metabolism pathway have been proposed as potential biomarkers, highlighting the importance of 17(S)-HpDHA in immune-related processes (Butovich et al., 2005).

Additionally, 4-Methyl-5-thiazoleethanol, which showed marked upregulation in the positive ion mode, has not been widely studied as a biomarker in immune diseases. However, its significant differential expression in our study suggests it could represent a novel biomarker candidate—not only for distinguishing OAPS from OAPS + UCTD but also potentially for other immune-mediated conditions. Further investigation into its role in immune regulation may uncover new diagnostic and therapeutic targets.

This study revealed that, under the negative ion mode, compared with the UCTD group, the OAPS group had 14 significantly upregulated metabolites and four downregulated metabolites. The differential molecules were mainly enriched in the general metabolic pathway, arginine and proline metabolism, and the ABC transporters pathway. In patients with OAPS, hormonal dysregulation and immune homeostasis imbalance drive an overall increase in metabolic activity (Miyara and Sakaguchi, 2011). Extensive activation of the primary pathway may lead to characteristic reallocation of metabolic resources in certain secondary pathways through substrate competition and metabolic feedback mechanisms. The upregulation of metabolites related to arginine and proline metabolism is closely associated with the dual demands of the pathological state (Li B. et al., 2024).

The enrichment of differential metabolites in the ABC transporters pathway indicates altered activity in the membrane transport system. This pathway primarily contributes to the regulation of cell membrane homeostasis by modulating mechanisms such as drug efflux and the transport of inflammatory mediators, and may be associated with the development of drug tolerance during the clinical treatment of patients with OAPS (Lu et al., 2014).

The upregulated and downregulated metabolites identified in the comparison between OAPS and UCTD also hold implications for biomarker discovery in immune-related diseases. For example, 3-Hydroxybenzoic acid, which was significantly upregulated in OAPS compared to UCTD under the negative ion mode, has been associated with oxidative stress and inflammation—key processes in many immune-mediated diseases (Gonthier et al., 2003). In systemic lupus erythematosus, metabolites related to oxidative stress pathways have been proposed as biomarkers, suggesting that 3-Hydroxybenzoic acid could serve not only as a biomarker for OAPS but also for other immune-related conditions. 4-Methyl-5-thiazoleethanol, which again showed differential expression under the positive ion mode, further underscores its potential as a biomarker. Its consistent and significant variation across comparisons suggests it may be a key metabolite for distinguishing OAPS from UCTD and could also play a role in the pathophysiology of other, yet-to-be-identified immune diseases (Yang et al., 2024).

Under the positive ion mode, a total of 30 metabolites were upregulated and 32 metabolites were downregulated. The differential pathways involved six core metabolic pathways, including retrograde endocannabinoid signaling, linoleic acid metabolism, and others. Alterations in the retrograde endocannabinoid signaling pathway may reflect imbalances in central–peripheral immune regulation (Li F. et al., 2024). The endocannabinoid system may contribute to the regulation of OAPS-related neuroinflammation through retrograde signaling mediated by CB1/CB2 receptors (Baldassarr et al., 2008). Abnormalities in linoleic acid and α-linolenic acid metabolism suggest a disrupted ω-6/ω-3 polyunsaturated fatty acid ratio, which may promote the production of pro-inflammatory mediators (Taylor et al., 2022). The accumulation of glycerophospholipid metabolites is associated with membrane phospholipid remodeling and abnormal sphingolipid signal transduction (Jana and Pahan, 2010). Enrichment of the choline metabolism in cancer pathway implies a possible link between disordered phospholipid metabolism and abnormal regulation of cell proliferation under pathological conditions (Sonkar et al., 2019). Reactivation of the arachidonic acid metabolism pathway further confirms excessive immune cell activation (Tu et al., 2023). The prostaglandins and leukotrienes produced through the COX/LOX dual pathway may form a pro-inflammatory positive feedback loop (Liu et al., 2019).

By integrating data from both ion modes, this study found that metabolic abnormalities in OAPS exhibit multi-dimensional interactive characteristics: (1) Remodeling of lipid metabolism (arachidonic acid/linoleic acid pathways) and inflammatory responses form a bidirectional regulatory system; (2) ABC transporters and choline metabolism collectively constitute a membrane homeostasis regulatory network; (3) Arginine metabolism is linked to vascular endothelial dysfunction via the NO synthesis pathway. This coordinated imbalance across multiple pathways may serve as an important metabolic signature distinguishing OAPS from UCTD.

Through comparative analysis of the metabolomic characteristics of patients with OAPS complicated by UCTD and patients with UCTD alone, it was found that under the negative ion mode, 15 metabolites were upregulated and 15 were downregulated. The differential metabolites were mainly enriched in the general metabolic pathway (metabolic pathways), arginine and proline metabolism, and the vascular smooth muscle contraction pathway. Hormonal dysregulation and immune-inflammatory cascade reactions triggered by the comorbid disease state drive the remodeling of the cellular metabolic network, resulting in the disruption of metabolic homeostasis (Chen et al., 2024).

The significant enrichment of the arginine and proline metabolism pathway suggests a biphasic regulatory mechanism. Arginine is rapidly converted to NO via the inducible nitric oxide synthase (iNOS) pathway, participating in the regulation of vascular tone and the activation of immune cells (Bouzin et al., 2007). Enhanced proline synthesis may reflect a response to tissue damage caused by chronic inflammation, as proline serves as a critical precursor for collagen synthesis, particularly in skin and vascular lesions (Mahout et al., 2024). The abnormal enrichment of the vascular smooth muscle contraction pathway is highly consistent with clinical phenotypes (Jiang et al., 2021). Differential metabolites in this pathway may contribute to vascular vasomotor dysfunction by influencing calcium channel activity or altering the balance of vasoactive substances, offering a metabolic explanation for the increased thrombotic risk in patients with OAPS complicated by UCTD.

In the comparison between OAPS + UCTD and UCTD, the differentially expressed metabolites also have relevance to biomarker research in other immune diseases. Chlortetracycline, which was upregulated under the negative ion mode in patients with OAPS + UCTD, possesses known antibacterial and anti-inflammatory properties. In several chronic inflammatory diseases, antibiotics and their metabolites have been explored as potential biomarkers due to their interactions with the immune system. This suggests that chlortetracycline could serve as a relevant biomarker not only for distinguishing OAPS + UCTD from UCTD but also for understanding immune-microbial interactions in other immune-related conditions (Li et al., 2023). 6α-Prostaglandin I1, which was downregulated, plays a role in regulating platelet function and inflammation. In diseases such as atherosclerosis, which involves immune-inflammatory components, similar prostaglandin-related metabolites have been investigated as biomarkers. This underscores the importance of 6α-Prostaglandin I1 in immune-related processes and its potential utility as a biomarker across multiple immune-mediated diseases. Under the positive ion mode, 29 metabolites were found to be upregulated, and 64 were downregulated. The differential pathways involved six core pathways, including endocannabinoid signaling and polyunsaturated fatty acid metabolism. Endocannabinoids are known to exacerbate autoimmune responses by altering neuronal synaptic plasticity via CB1 receptors and disrupting macrophage polarization through CB2 receptor signaling (Chiurchiù et al., 2018).

Additionally, the synchronous activation of arachidonic acid, linoleic acid, and α-linolenic acid metabolism constitutes a “triad” of pro-inflammatory lipid mediators.

(1) the COX/LOX dual pathways promote the excessive production of inflammatory mediators such as PGE2 and LTB4 (Marcouiller et al., 2005); (2) an imbalanced ω-6/ω-3 polyunsaturated fatty acid ratio alters cell membrane phospholipid composition, affecting immune cell reactivity (Robinson et al., 2001); (3) insufficient synthesis of inflammation-resolving mediators such as lipoxins impairs the resolution phase of inflammation (Jackson et al., 2023). Co-occurring abnormalities in sphingolipid and choline metabolism further reveal disruptions in membrane homeostasis mechanisms: (1) alterations in the sphingosine-1-phosphate (S1P) gradient may impair lymphocyte migration (Lee et al., 2024); (2) disruptions in choline metabolism can affect the integrity of lipid rafts and interfere with Toll-like receptor signaling by altering phosphatidylcholine synthesis (Sanchez-Lopez et al., 2019).

Notably, two pathways—steroid hormone biosynthesis and retrograde endocannabinoid signaling—emerged as consistently enriched across multiple group comparisons, suggesting they represent conserved metabolic shifts in OAPS, UCTD, and their comorbid state. These pathways hold significant translational potential: (1) Steroid hormone biosynthesis: Enriched in B1 vs. B3 and B2 vs. B3 (both ion modes), this pathway links to the well-established hormonal dysregulation in autoimmune diseases (Ye et al., 2023). Key metabolites in this pathway showed AUC values >0.8 in ROC analyses, supporting their utility as part of a multi-marker panel. Clinically, steroid hormones modulate immune cell function and vascular reactivity (Santos et al., 2022), making this pathway a candidate for targeted therapies to balance immune-vascular crosstalk in OAPS; (2) Retrograde endocannabinoid signaling: Consistently enriched in B1 vs. B3 and B2 vs. B3 (positive ion mode), this pathway regulates immune cell migration and neuroinflammatory responses via CB1/CB2 receptors (Li X. et al., 2018). Metabolites such as anandamide could serve as biomarkers, while targeting this pathway with CB2 agonists might suppress pro-inflammatory cytokine release without central nervous system side effects (Osorio-Perez et al., 2025). These conserved pathways, by bridging metabolism, immunity, and vascular function, offer both diagnostic and therapeutic opportunities to improve clinical management of these challenging conditions.

Clinically, the AI-guided metabolic classifier holds promise for two key applications: (1) Early diagnosis of ambiguous cases, particularly the comorbid B2 subgroup, where overlapping clinical features often delay classification—our model’s AUC of 0.89 for B2 vs. B1/B3 surpasses standard serological tests. (2) Stratified treatment, as enriched pathways point to targeted interventions for inflammation-driven B2 cases, versus vascular-focused therapies for lipid metabolism-dominant B1.

A key limitation is the potential for false positives or unresolved isomers in metabolite identification. While our tiered approach (MS/MS similarity >0.8, mass error <5 ppm) reduces this risk, structural isomers may be conflated due to overlapping MS/MS spectra. Additionally, low-abundance metabolites (Level 3 annotations) carry higher false discovery risk. Future studies should integrate authentic standards for Level 1 validation and ion mobility spectrometry to resolve isomeric ambiguity, enhancing the specificity of identified metabolic signatures. Moreover, the small sample size and single-center design, may restrict generalizability—multi-center validation with larger cohorts is needed. Additionally, metabolic profiles may be confounded by concurrent medications, highlighting the need for prospective studies controlling for such variables. Future multi-center studies with larger cohorts will incorporate multivariate regression models to adjust for these variables, further validating the stability of the identified metabolic signatures. Furthermore, the detailed epidemiological variables such as body mass index, smoking status, and dietary habits were not systematically recorded. These factors could potentially confound metabolic profiles, as they are known to influence lipid and amino acid metabolism—pathways enriched in our analysis. Finally, our cohort exclusively included female participants, reflecting the strong association of OAPS with obstetric outcomes and the higher prevalence of UCTD in reproductive-aged women. However, this excludes the possibility of exploring gender-specific metabolic differences, particularly in UCTD, where male patients may exhibit distinct pathophysiological features. Future studies will incorporate comprehensive epidemiological data collection and include male participants to address these gaps, enhancing the generalizability of our findings.

## Conclusion

This study, through multi-level metabolomics analysis, systematically characterized the metabolic reprogramming features of OAPS, UCTD, and their comorbid state, providing essential molecular insights into their pathophysiological mechanisms. The identification of differential metabolites and related pathways not only revealed potential biomarker clusters but also helped establish a theoretical framework for the interactive regulation of the “metabolism–immunity–vascular system,” laying the groundwork for developing early diagnostic models and individualized treatment strategies based on metabolic interventions.

However, several aspects require further investigation.

(1) The broad-spectrum detection capabilities of untargeted metabolomics may introduce identification bias for low-abundance metabolites. Future studies should integrate targeted validation methods (e.g., LC-MS/MS quantification) and spatial metabolomics techniques (e.g., MALDI-IM-MS) to enhance detection accuracy.
(2) Disease subtype heterogeneity (e.g., variations in antiphospholipid antibody profiles) and treatment-related confounding factors (e.g., use of heparin or hormones) may affect the specificity of metabolic signatures. It is recommended to expand sample sizes through multi-center cohort studies and implement stratified analysis to control for these variables.
(3) Current analyses remain correlative in nature. To determine causality, disease animal models should be developed, and the functional relevance of key metabolic pathways should be validated using interventions such as metabolic enzyme inhibitors or isotope-labeled metabolic flux analysis.

## Data Availability

The original contributions presented in the study are included in the article/Supplementary Material, further inquiries can be directed to the corresponding authors.
