# Supplementary material for: Untargeted metabolomics reveals the metabolic characteristics and biomarkers of obstetric antiphospholipid syndrome and undifferentiated connective tissue disease
Source: Front Mol Biosci. 2025 Aug 22;12:1632244. doi: 10.3389/fmolb.2025.1632244 (PMC12411200; doi:10.3389/fmolb.2025.1632244)
Supplement: Supplementary file 2 [file Supplementaryfile3.docx]

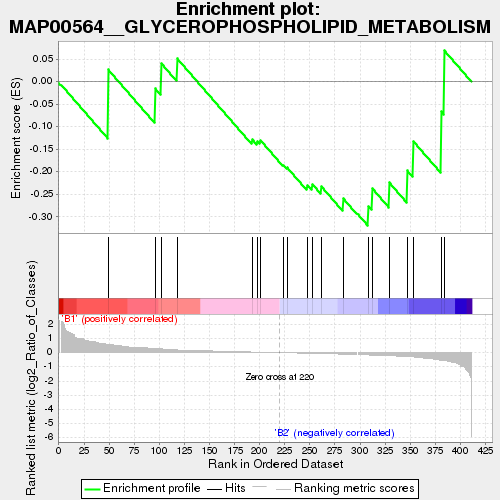


Figure S1. The results of the GSEA for GLYCEROPHOSPHOLIPID_METABOLISM conducted on Group B1 and Group B2 under the negative ion mode.


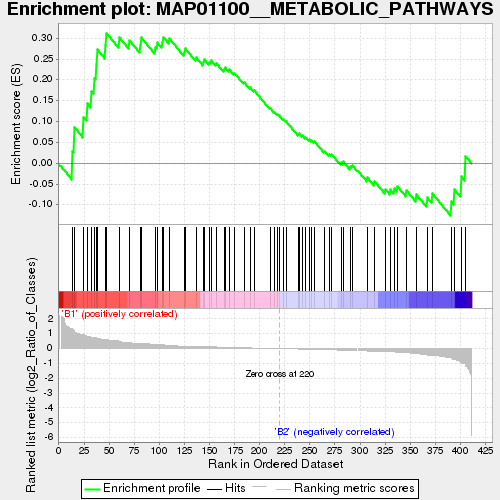


Figure S2. The results of the GSEA for METABOLIC_PATHWAYS

conducted on Group B1 and Group B2 under the negative ion mode.


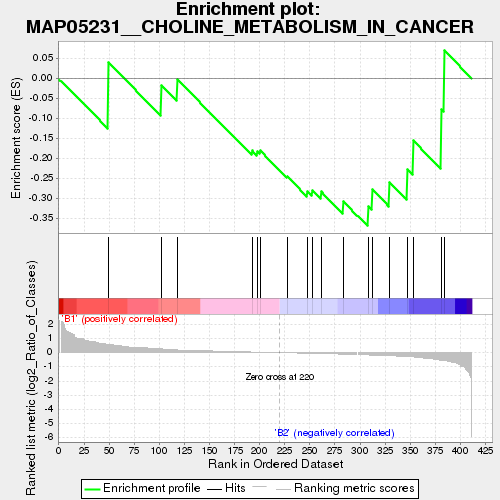


Figure S3. The results of the GSEA for CHOLINE_METABOLISM_IN_CANCER

conducted on Group B1 and Group B2 under the negative ion mode.


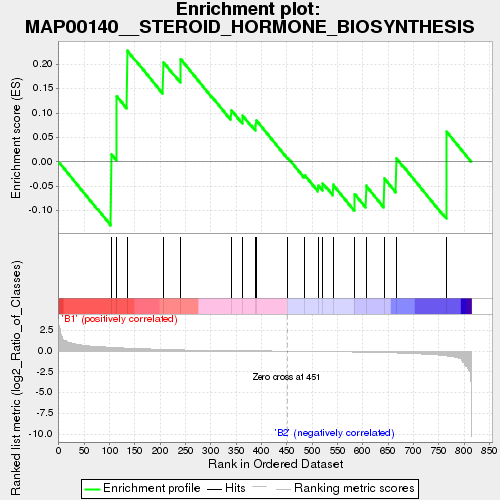


Figure S4. The results of the GSEA for STEROID_HORMONE_BIOSYNTHESIS

conducted on Group B1 and Group B2 under the positive ion mode.


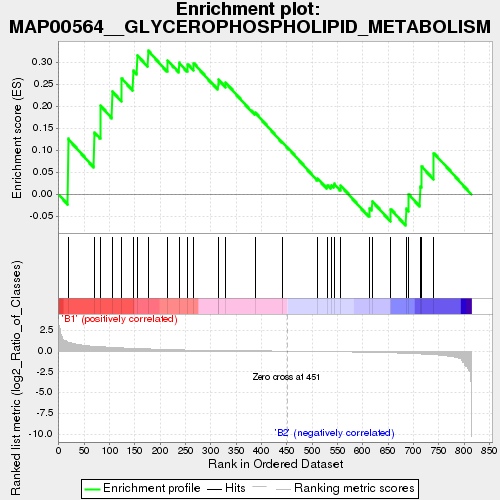


Figure S5. The results of the GSEA for GLYCEROPHOSPHOLIPID_METABOLISM

conducted on Group B1 and Group B2 under the positive ion mode.


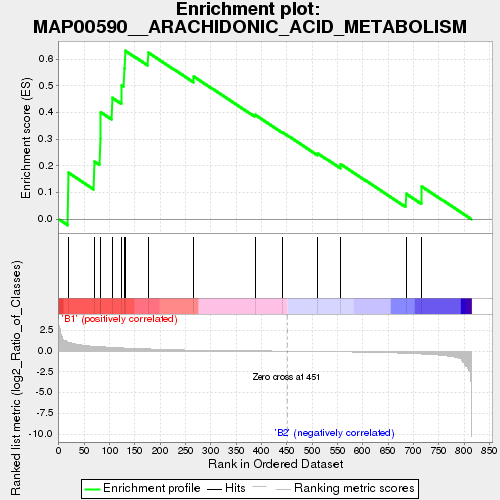


Figure S6. The results of the GSEA for ARACHIDONIC_ACID_METABOLISM

conducted on Group B1 and Group B2 under the positive ion mode.


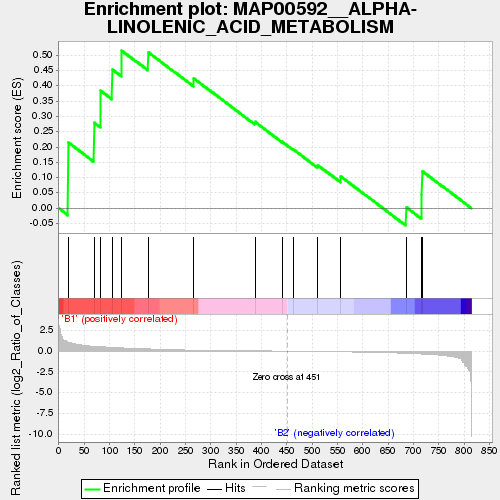


Figure S7.The results of the GSEA for ALPHA-LINOLENIC_ACID_METABOLISM conducted on Group B1 and Group B2 under the positive ion mode.


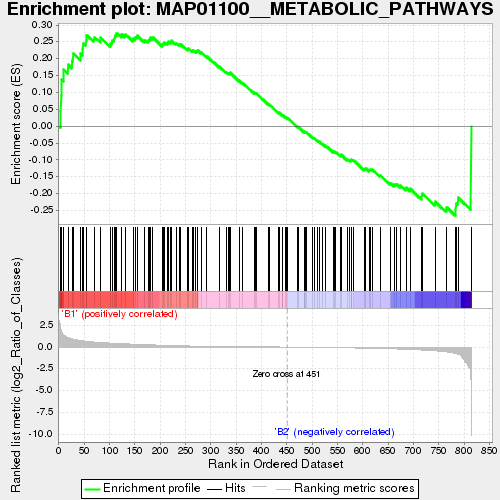


Figure S8. The results of the GSEA for METABOLIC_PATHWAYS conducted on Group B1 and Group B2 under the positive ion mode.


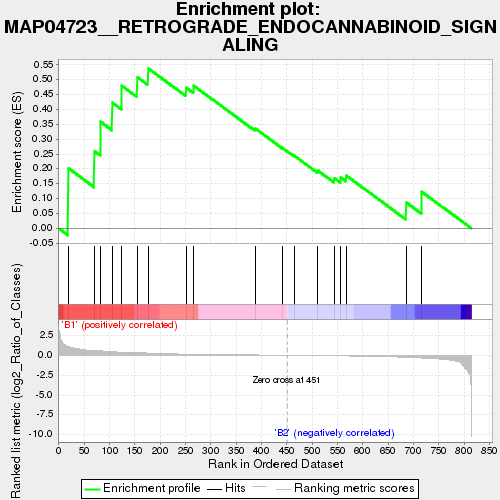


Figure S9. The results of the GSEA for RETROGRADE_ENDOCANNABINOID_SIGNALING conducted on Group B1 and Group B2 under the positive ion mode.


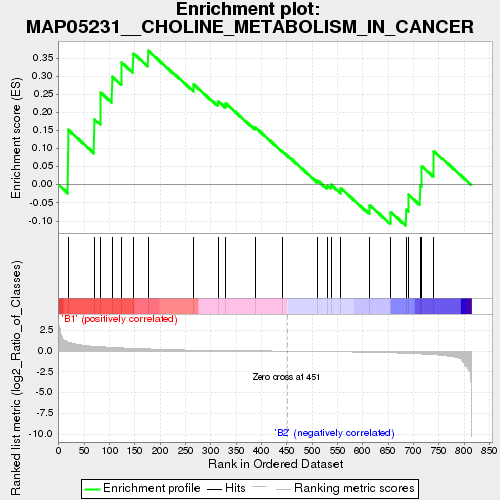


Figure S10. The results of the GSEA for CHOLINE_METABOLISM_IN_CANCER conducted on Group B1 and Group B2 under the positive ion mode.


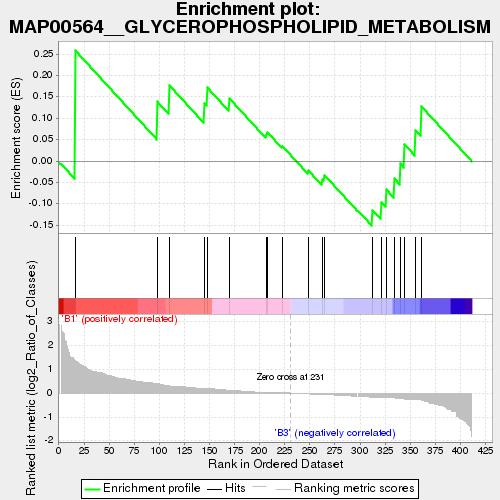


Figure S11. The results of the GSEA for GLYCEROPHOSPHOLIPID_METABOLISM conducted on Group B1 and Group B3 under the negative ion mode.


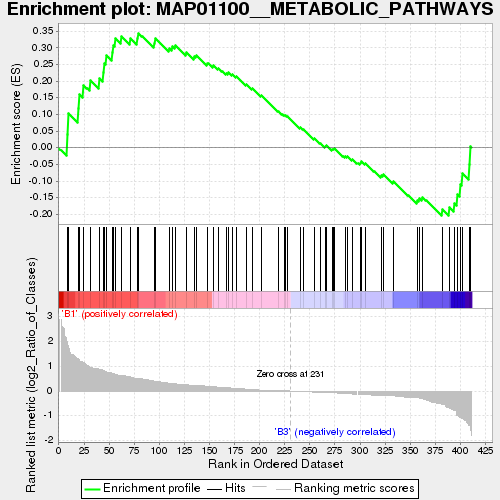


Figure S12. The results of the GSEA for METABOLIC_PATHWAYS conducted on Group B1 and Group B3 under the negative ion mode.


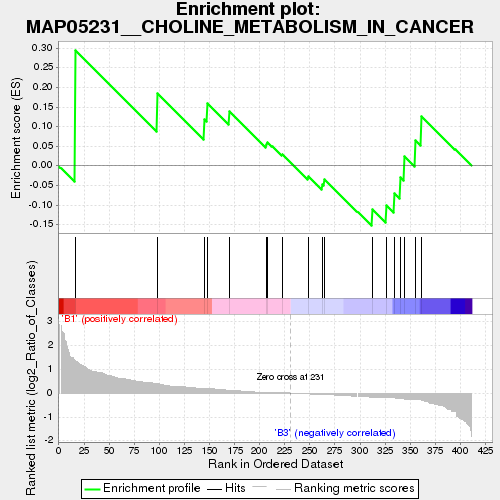


Figure S13. The results of the GSEA for CHOLINE_METABOLISM_IN_CANCER conducted on Group B1 and Group B3 under the negative ion mode.


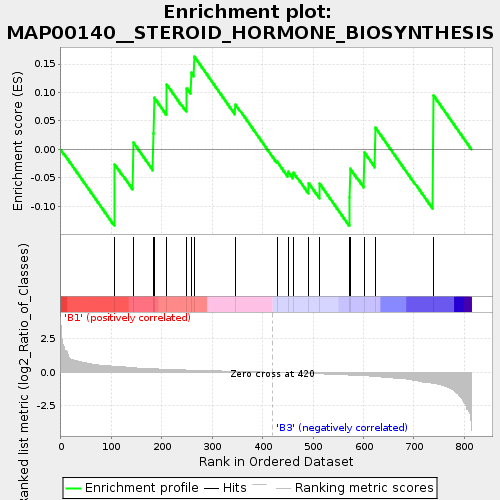


Figure S14. The results of the GSEA for STEROID_HORMONE_BIOSYNTHESIS conducted on Group B1 and Group B3 under the positive ion mode.


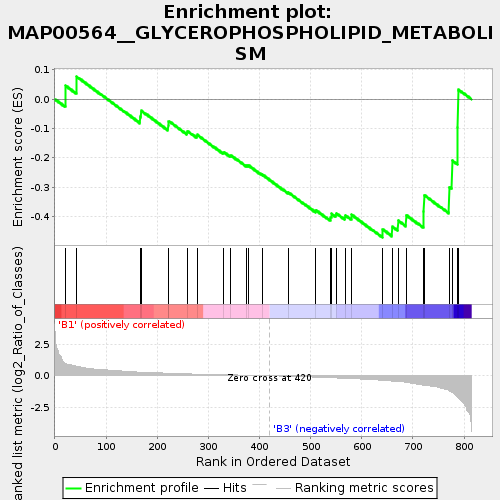


Figure S15. The results of the GSEA for GLYCEROPHOSPHOLIPID_METABOLISM conducted on Group B1 and Group B3 under the positive ion mode.


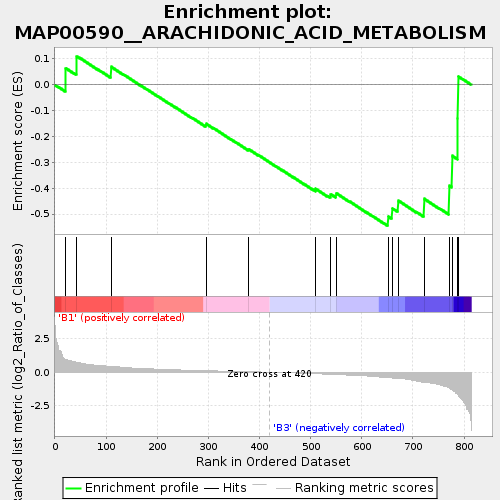


Figure S16. The results of the GSEA for ARACHIDONIC_ACID_METABOLISM conducted on Group B1 and Group B3 under the positive ion mode.


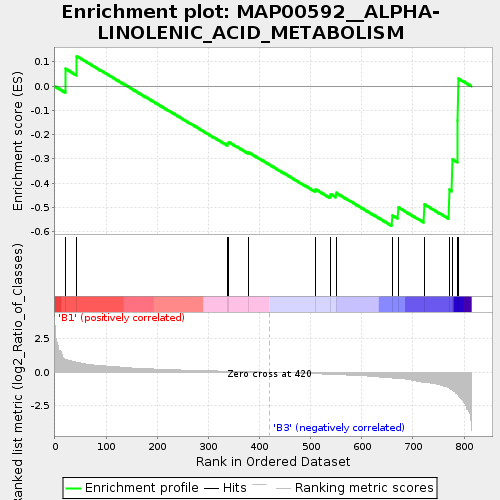


Figure S17. The results of the GSEA for ALPHA-LINOLENIC_ACID_METABOLISM conducted on Group B1 and Group B3 under the positive ion mode.


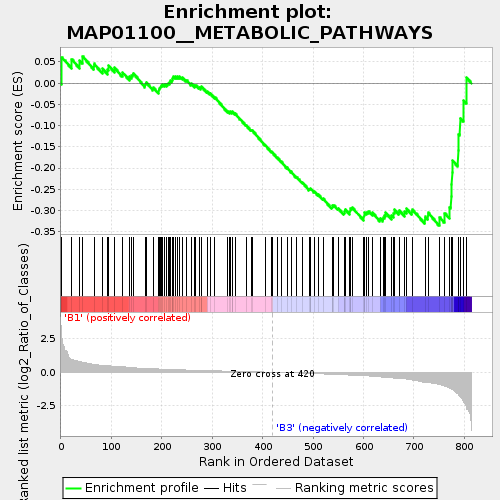


Figure S18. The results of the GSEA for METABOLIC_PATHWAYS conducted on Group B1 and Group B3 under the positive ion mode.


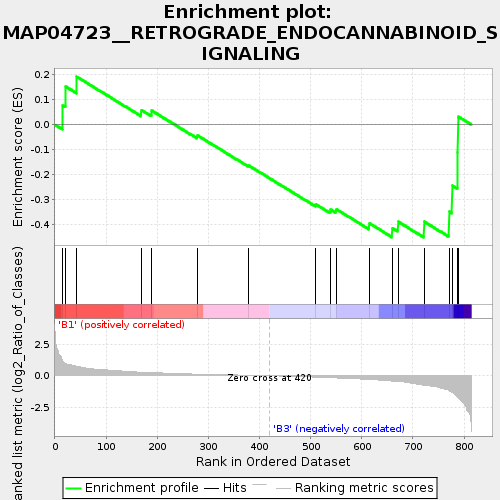


Figure S19. The results of the GSEA for RETROGRADE_ENDOCANNABINOID_SIGNALING conducted on Group B1 and Group B3 under the positive ion mode.


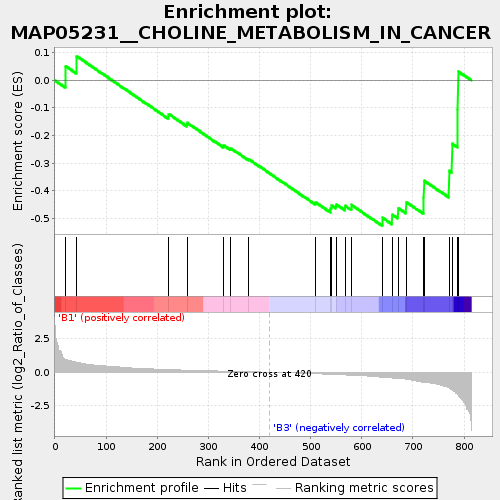


Figure S20. The results of the GSEA for CHOLINE_METABOLISM_IN_CANCER conducted on Group B1 and Group B3 under the positive ion mode.


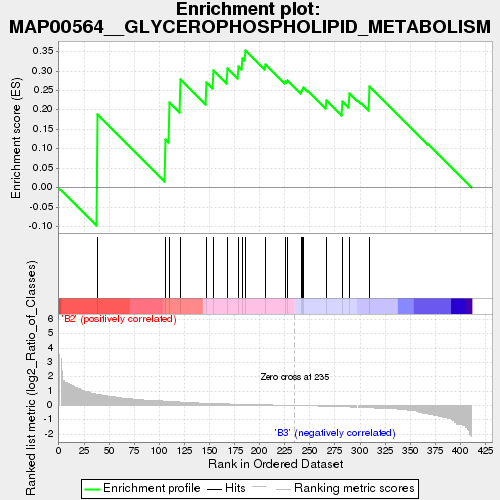


Figure S21. The results of the GSEA for GLYCEROPHOSPHOLIPID_METABOLISM conducted on Group B2 and Group B3 under the negative ion mode.


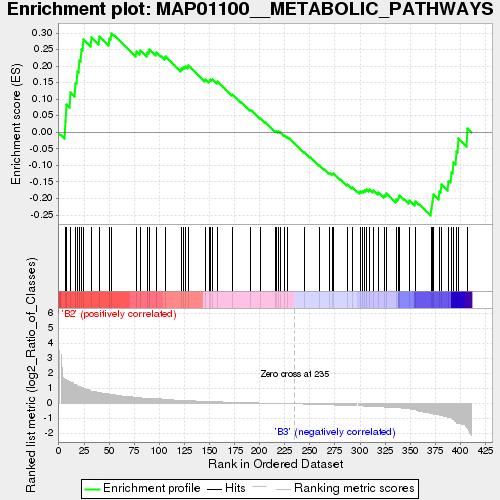


Figure S22. The results of the GSEA for METABOLIC_PATHWAYS conducted on Group B2 and Group B3 under the negative ion mode.


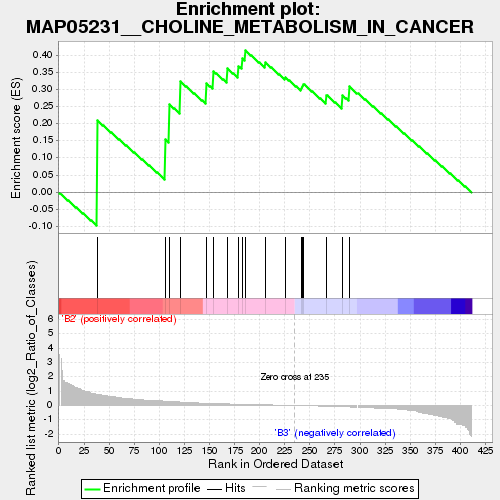


Figure S23. The results of the GSEA for CHOLINE_METABOLISM_IN_CANCER conducted on Group B2 and Group B3 under the negative ion mode.


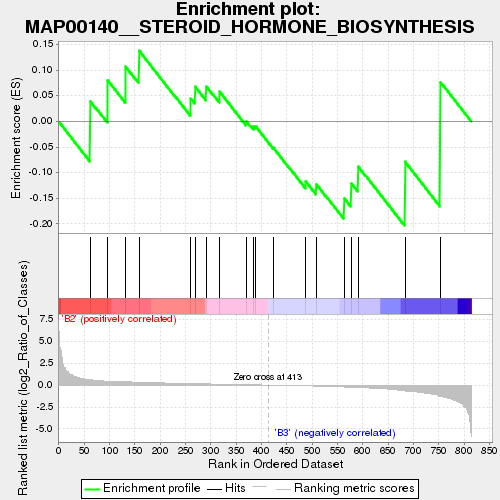


Figure S24. The results of the GSEA for STEROID_HORMONE_BIOSYNTHESIS conducted on Group B2 and Group B3 under the positive ion mode.


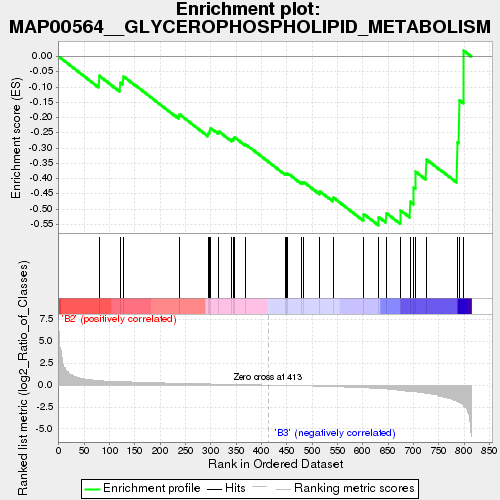


Figure S25. The results of the GSEA for GLYCEROPHOSPHOLIPID_METABOLISM conducted on Group B2 and Group B3 under the positive ion mode.


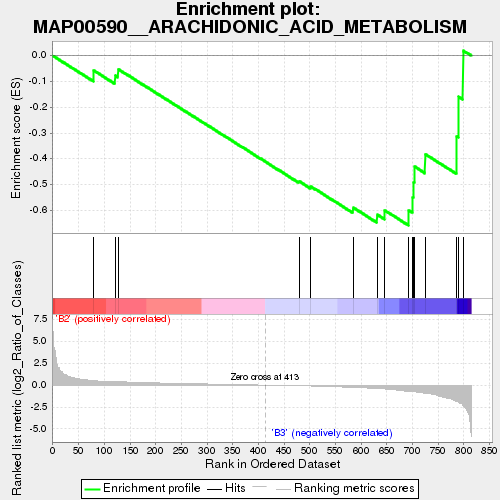


Figure S26. The results of the GSEA for ARACHIDONIC_ACID_METABOLISM conducted on Group B2 and Group B3 under the positive ion mode.


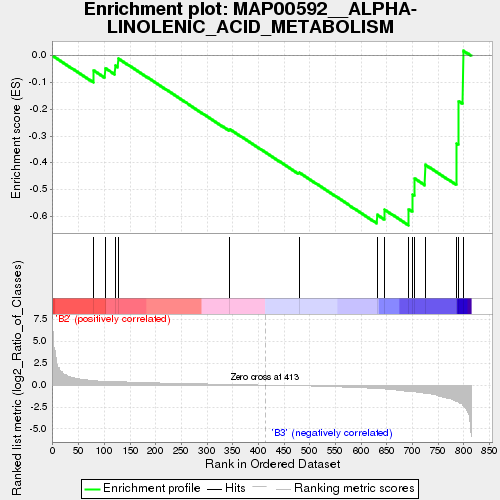


Figure S27. The results of the GSEA for ALPHA-LINOLENIC_ACID_METABOLISM conducted on Group B2 and Group B3 under the positive ion mode.


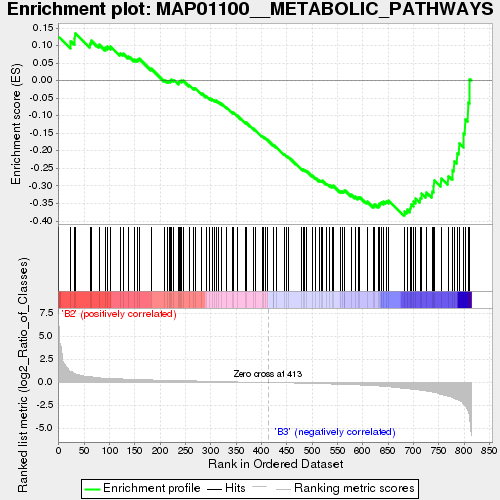


Figure S28. The results of the GSEA for METABOLIC_PATHWAYS conducted on Group B2 and Group B3 under the positive ion mode.


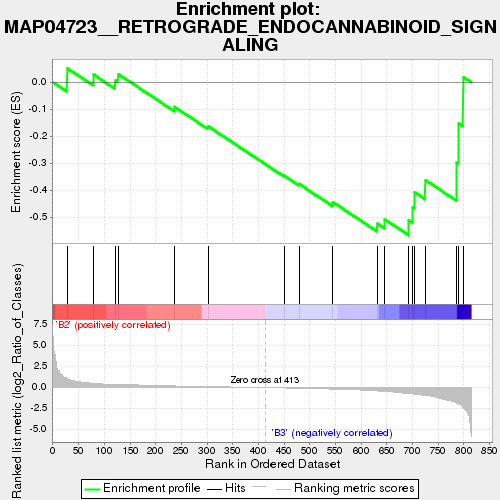


Figure S29. The results of the GSEA for RETROGRADE_ENDOCANNABINOID_SIGNALING conducted on Group B2 and Group B3 under the positive ion mode.


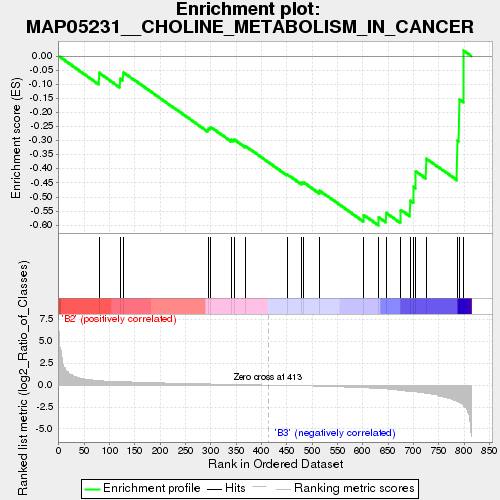


Figure S30. The results of the GSEA for CHOLINE_METABOLISM_IN_CANCER conducted on Group B2 and Group B3 under the positive ion mode.
