# Supplementary material for: Untargeted metabolomics reveals the metabolic characteristics and biomarkers of obstetric antiphospholipid syndrome and undifferentiated connective tissue disease
Source: Front Mol Biosci. 2025 Aug 22;12:1632244. doi: 10.3389/fmolb.2025.1632244 (PMC12411200; doi:10.3389/fmolb.2025.1632244)
Supplement: Supplementary file 3 [file Supplementaryfile2.docx]

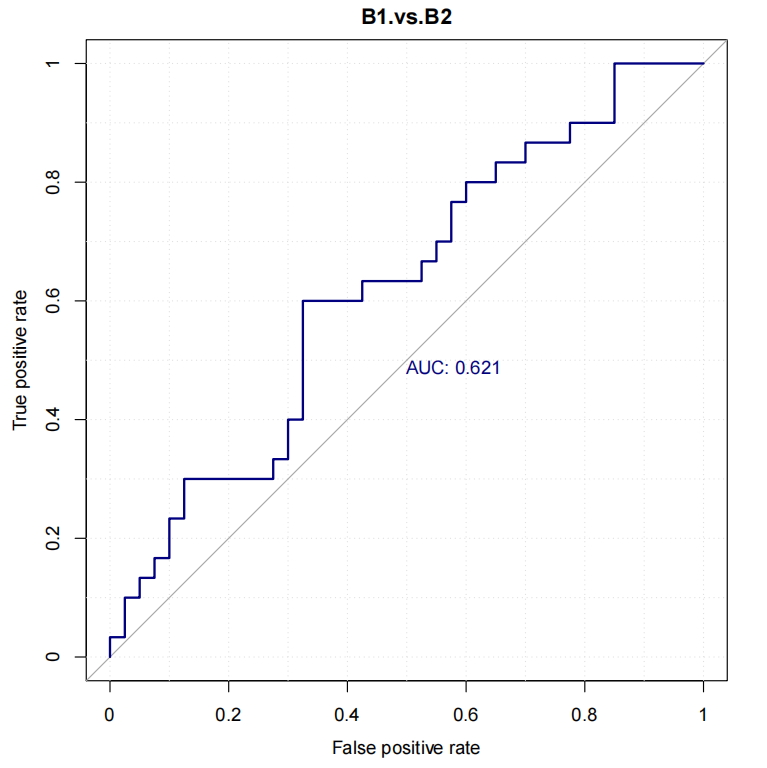


Figure S1: The Discriminatory Ability of Differential Metabolite 659 between Group B1 and Group B2 under the Negative Ion Mode


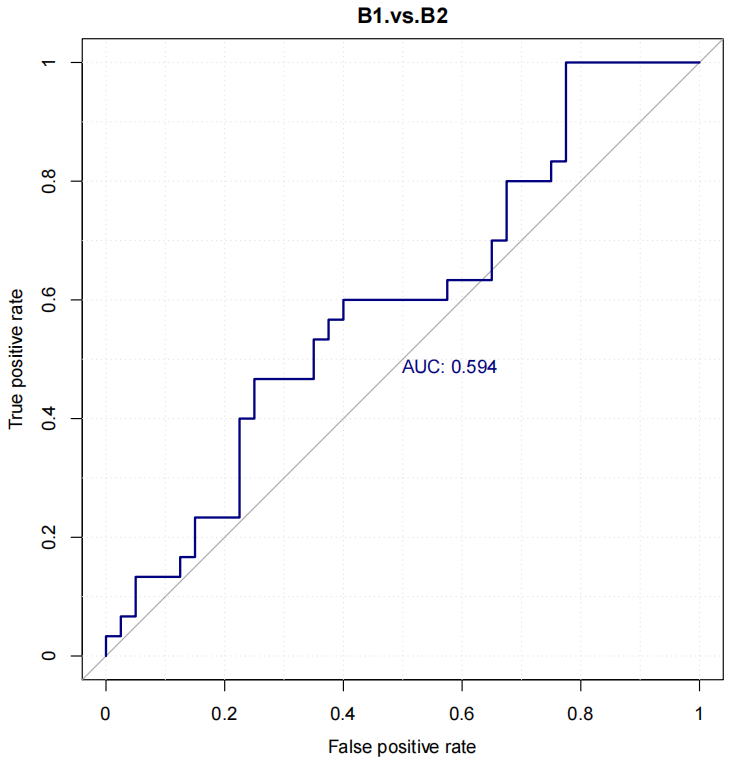


Figure S2: The Discriminatory Ability of Differential Metabolite 670 between Group B1 and Group B2 under the Negative Ion Mode


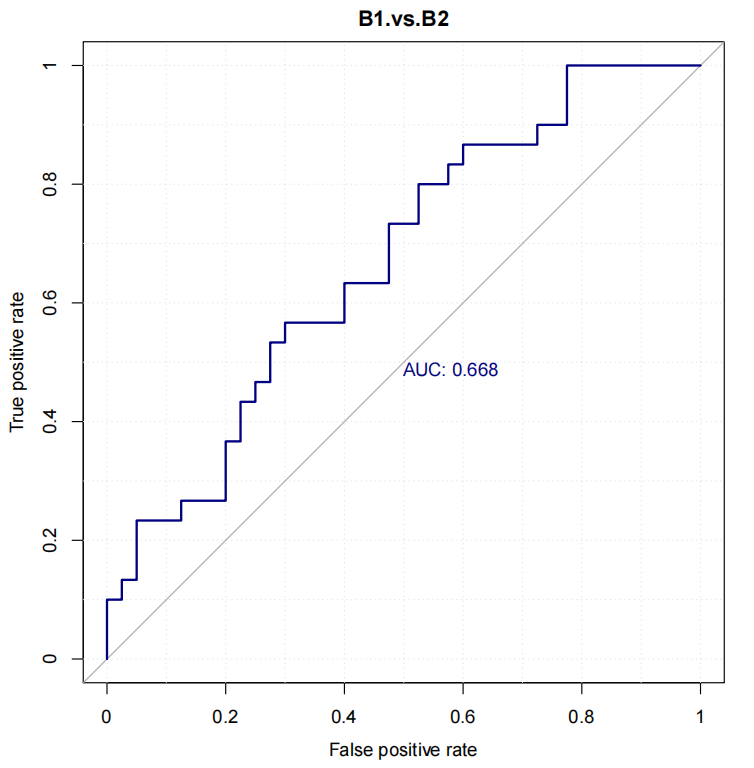


Figure S3: The Discriminatory Ability of Differential Metabolite 775 between Group B1 and Group B2 under the Negative Ion Mode


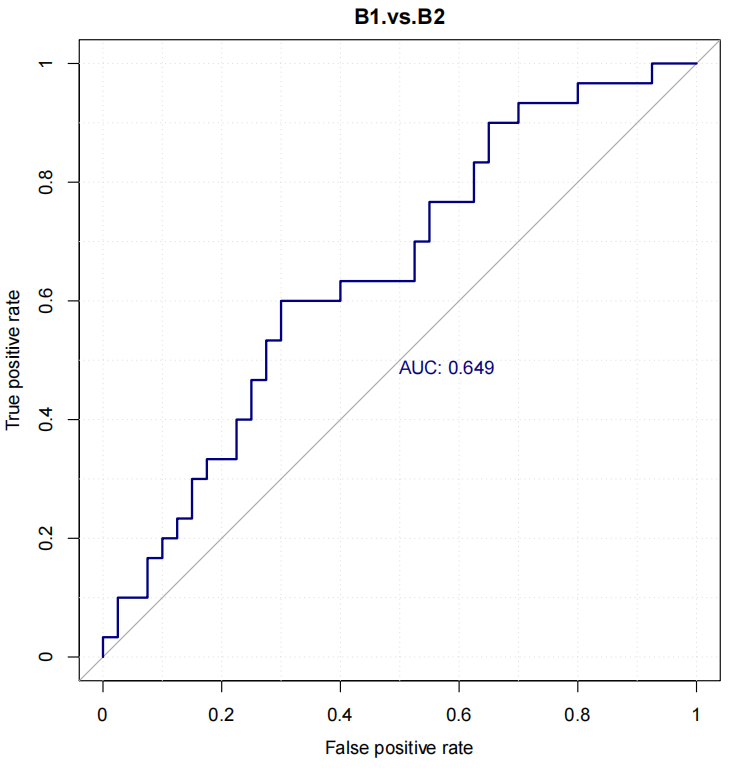


Figure S4: The Discriminatory Ability of Differential Metabolite 1262 between Group B1 and Group B2 under the Negative Ion Mode


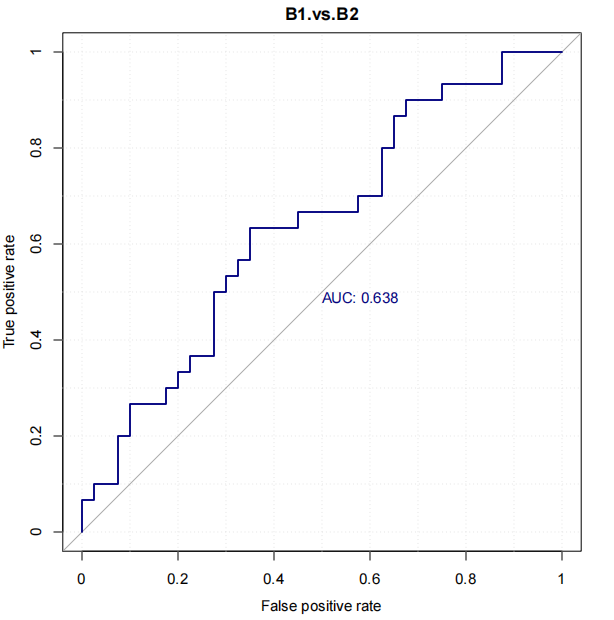


Figure S5: The Discriminatory Ability of Differential Metabolite 1593 between Group B1 and Group B2 under the Negative Ion Mode


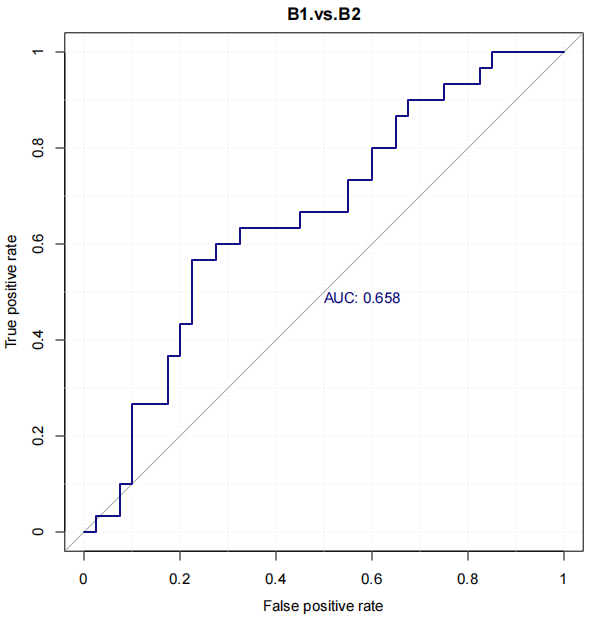


Figure S6: The Discriminatory Ability of Differential Metabolite 2016 between Group B1 and Group B2 under the Negative Ion Mode


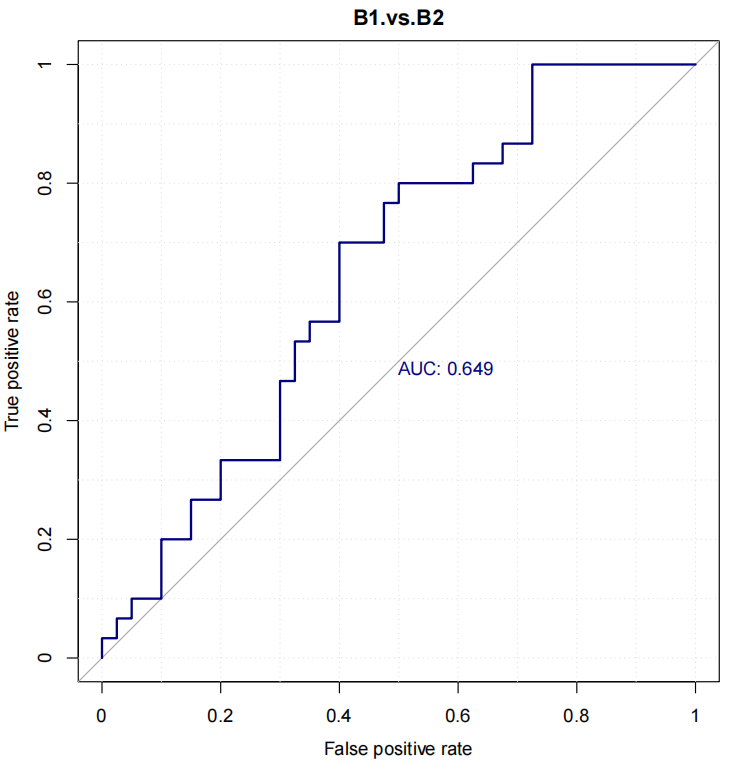


Figure S7: The Discriminatory Ability of Differential Metabolite 2843 between Group B1 and Group B2 under the Negative Ion Mode


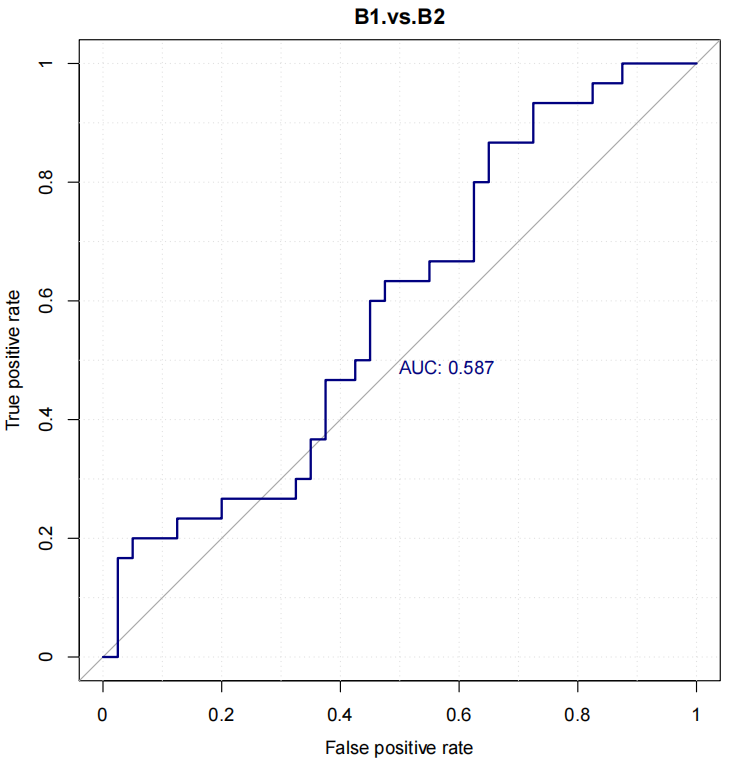


Figure S8: The Discriminatory Ability of Differential Metabolite 3227 between Group B1 and Group B2 under the Negative Ion Mode


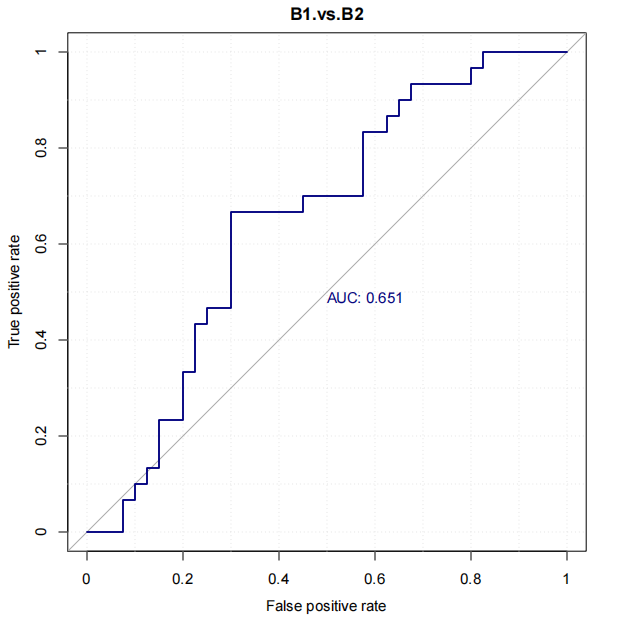


Figure S9: The Discriminatory Ability of Differential Metabolite 5646 between Group B1 and Group B2 under the Negative Ion Mode


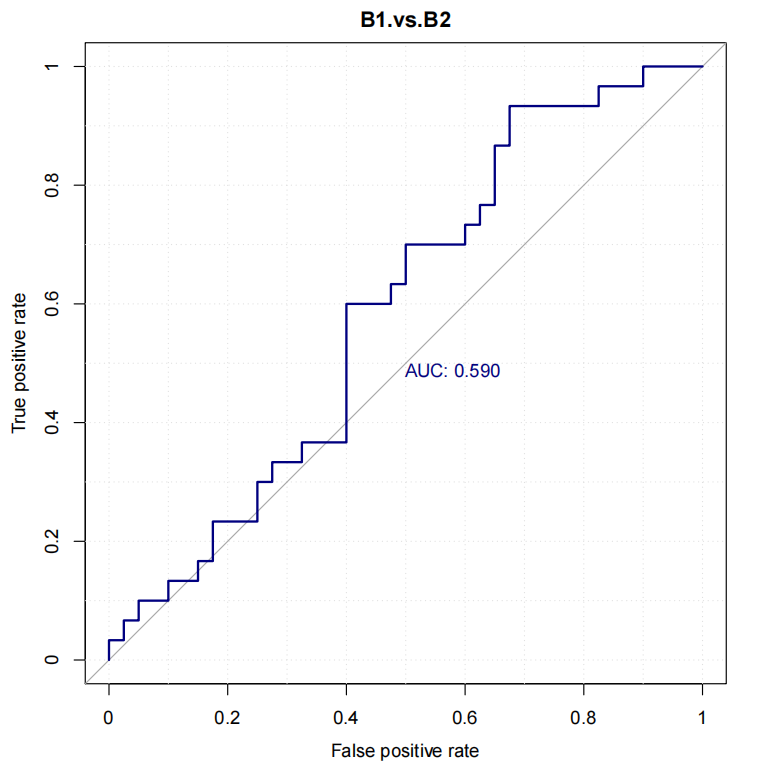


Figure S10: The Discriminatory Ability of Differential Metabolite 7769 between Group B1 and Group B2 under the Negative Ion Mode


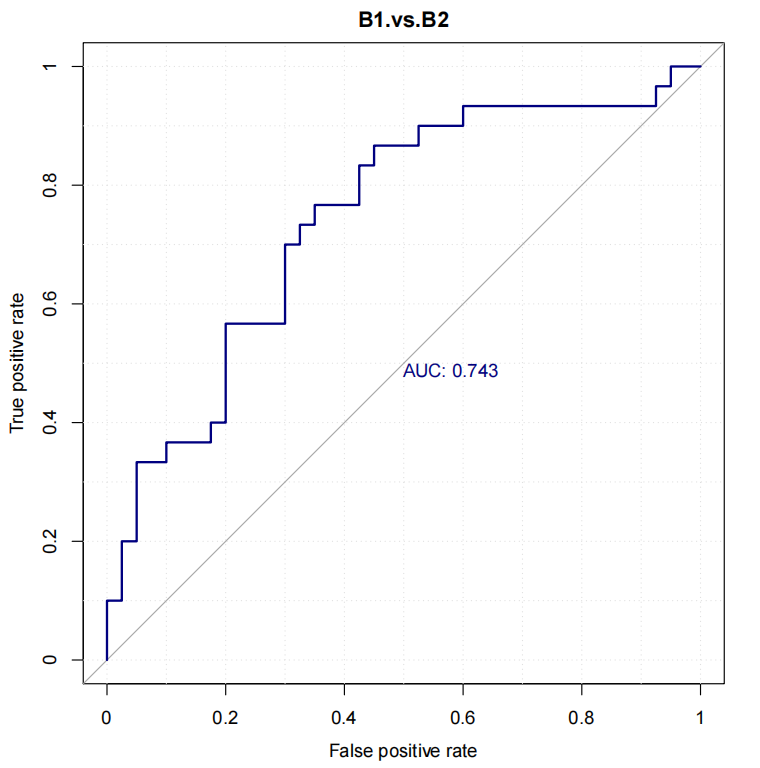


Figure S11: The Discriminatory Ability of Differential Metabolite 16 between Group B1 and Group B2 under the Positive Ion Mode


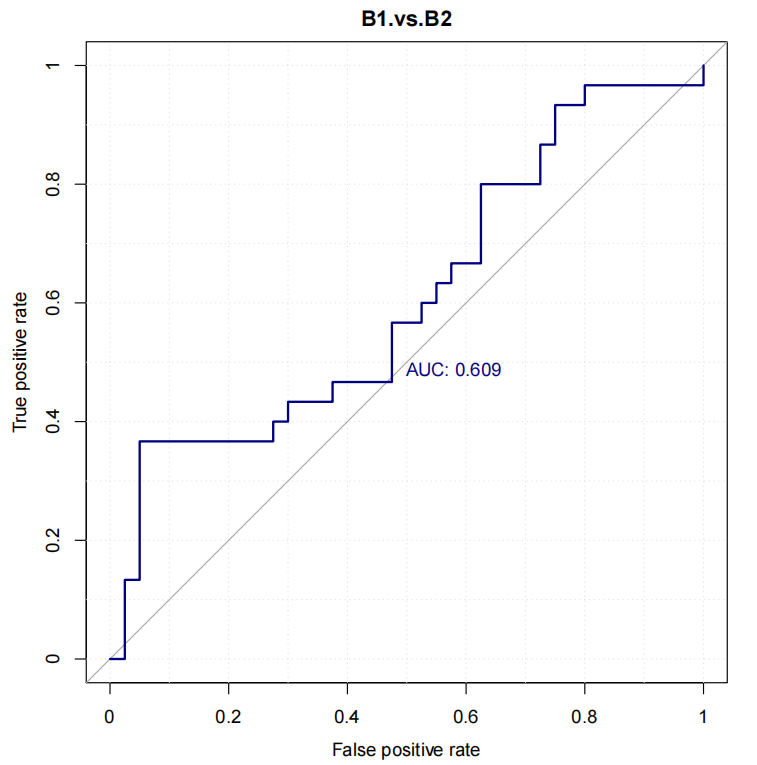


Figure S12: The Discriminatory Ability of Differential Metabolite 285 between Group B1 and Group B2 under the Positive Ion Mode


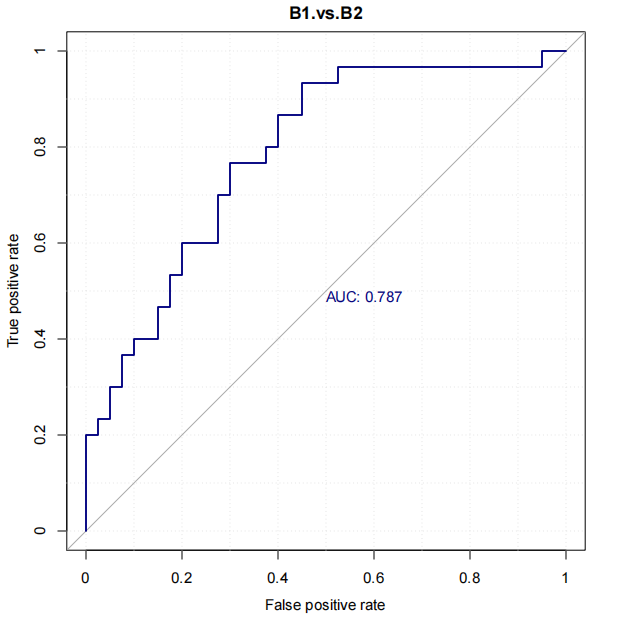


Figure S13: The Discriminatory Ability of Differential Metabolite 434 between Group B1 and Group B2 under the Positive Ion Mode


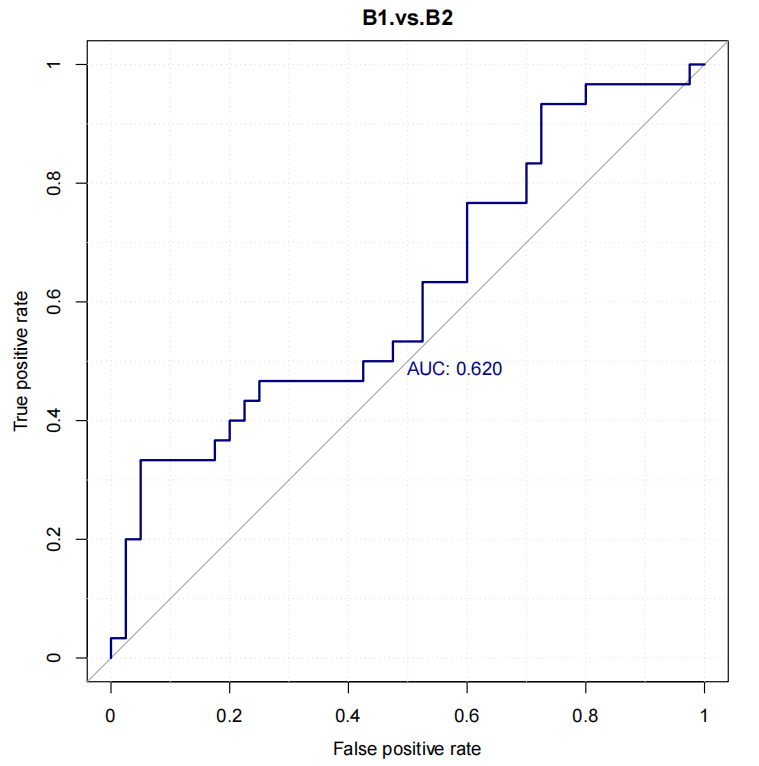


Figure S14: The Discriminatory Ability of Differential Metabolite 1063 between Group B1 and Group B2 under the Positive Ion Mode


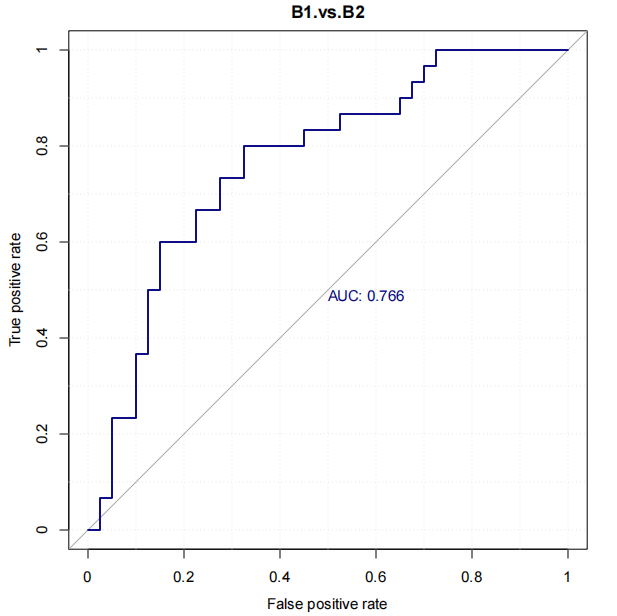


Figure S15: The Discriminatory Ability of Differential Metabolite 1485 between Group B1 and Group B2 under the Positive Ion Mode


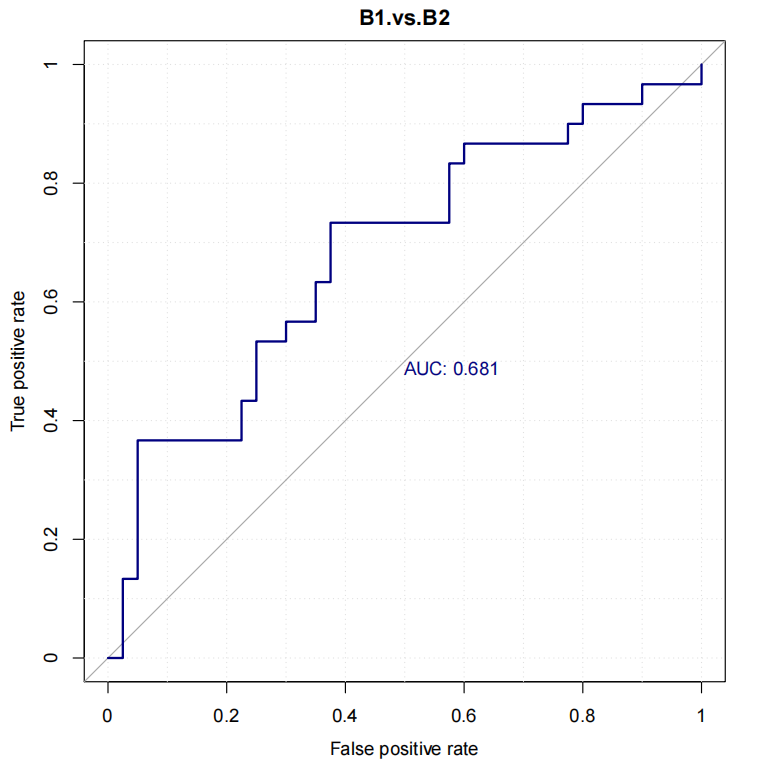


Figure S16: The Discriminatory Ability of Differential Metabolite 2241 between Group B1 and Group B2 under the Positive Ion Mode


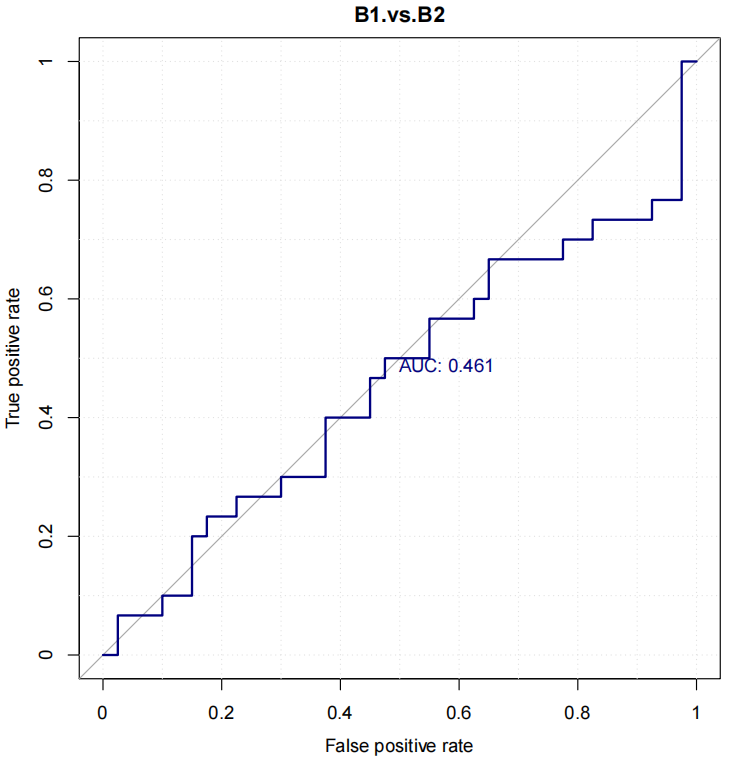


Figure S17: The Discriminatory Ability of Differential Metabolite 3566 between Group B1 and Group B2 under the Positive Ion Mode


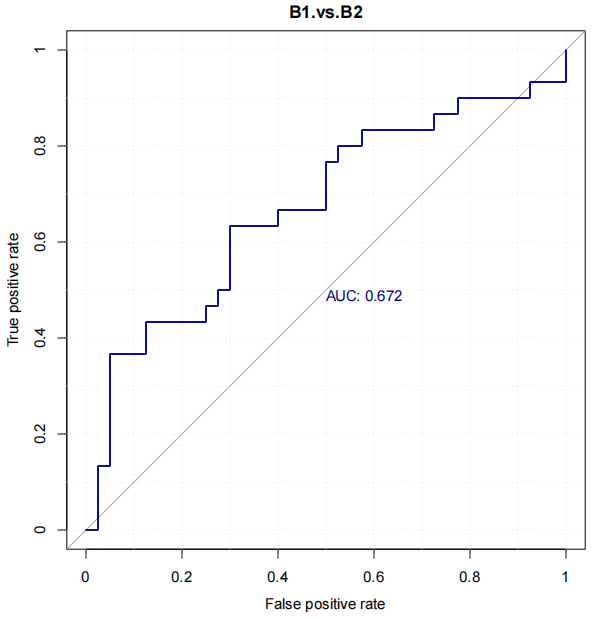


Figure S18: The Discriminatory Ability of Differential Metabolite 3640 between Group B1 and Group B2 under the Positive Ion Mode


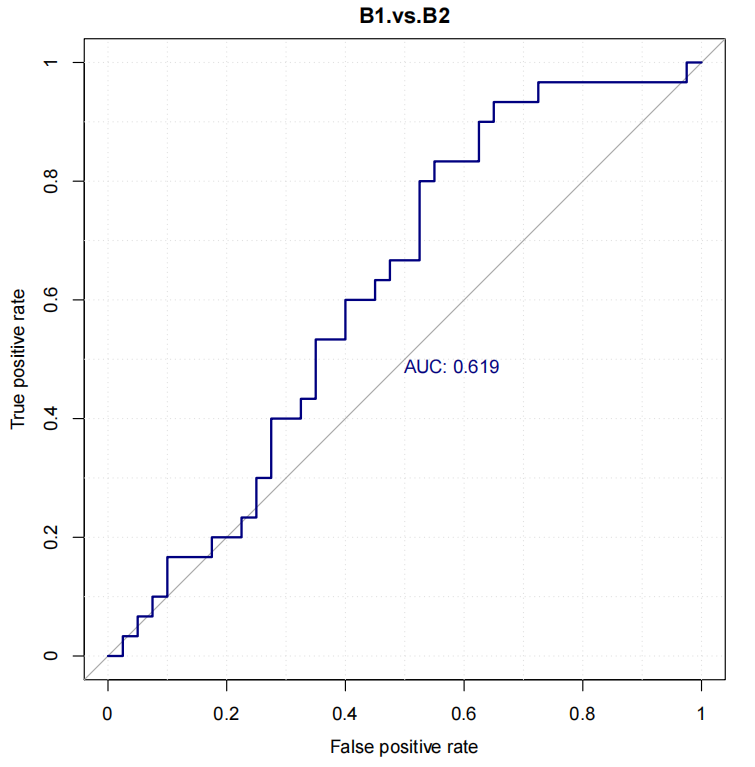


Figure S19: The Discriminatory Ability of Differential Metabolite 3662 between Group B1 and Group B2 under the Positive Ion Mode


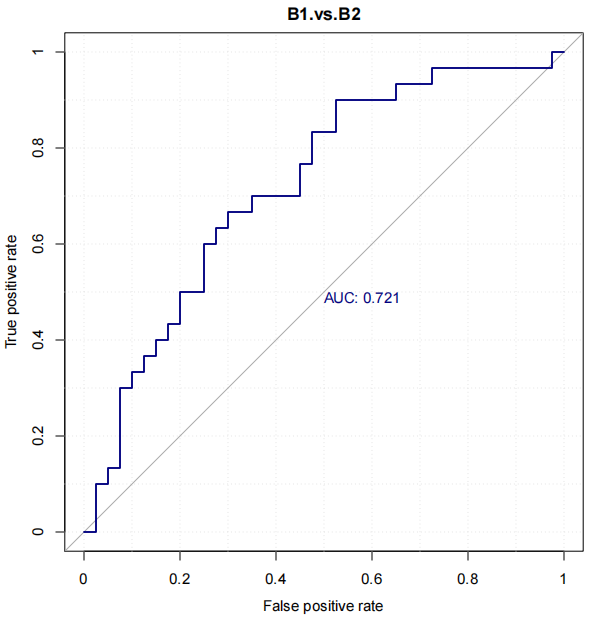


Figure S20: The Discriminatory Ability of Differential Metabolite 3854 between Group B1 and Group B2 under the Positive Ion Mode


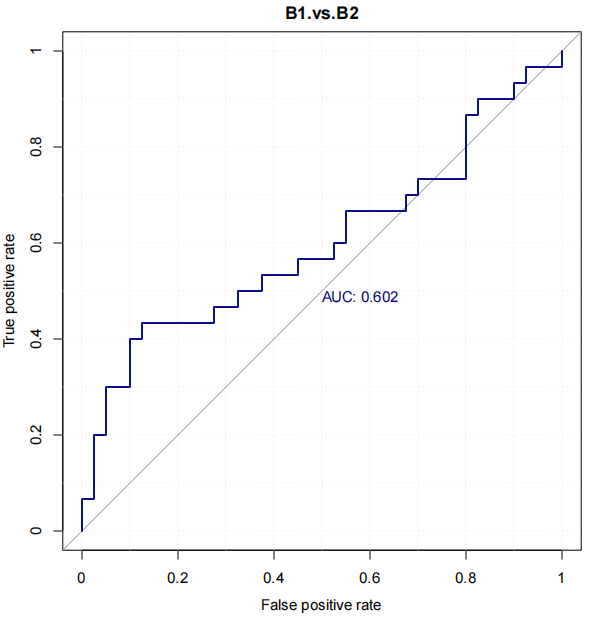


Figure S21: The Discriminatory Ability of Differential Metabolite 3881 between Group B1 and Group B2 under the Positive Ion Mode


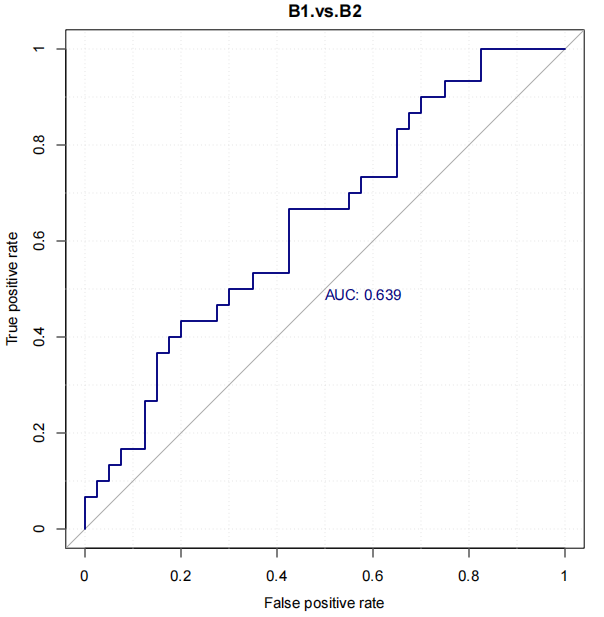


Figure S22: The Discriminatory Ability of Differential Metabolite 5657 between Group B1 and Group B2 under the Positive Ion Mode


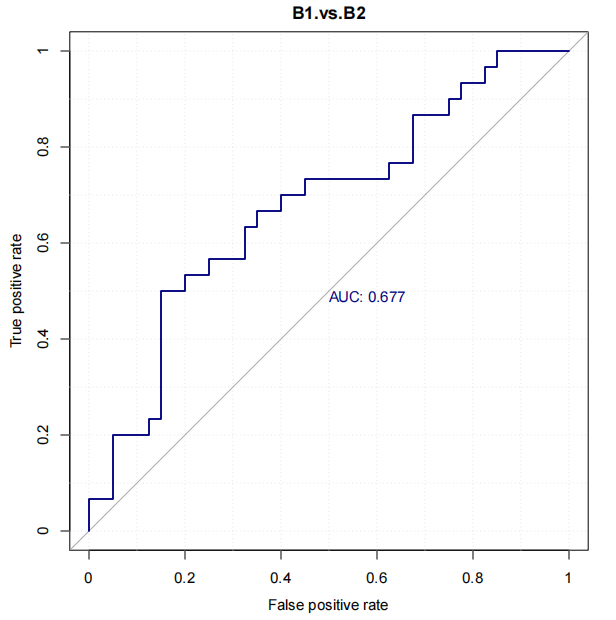


Figure S23: The Discriminatory Ability of Differential Metabolite 6397 between Group B1 and Group B2 under the Positive Ion Mode


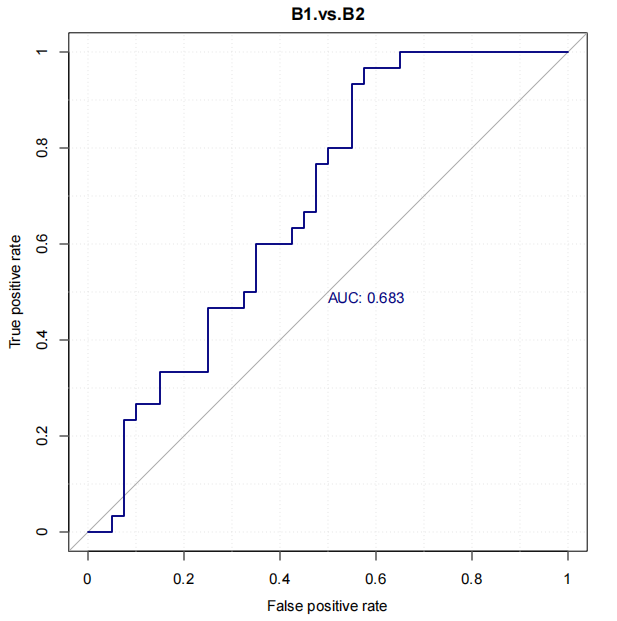


Figure S24: The Discriminatory Ability of Differential Metabolite 7135 between Group B1 and Group B2 under the Positive Ion Mode


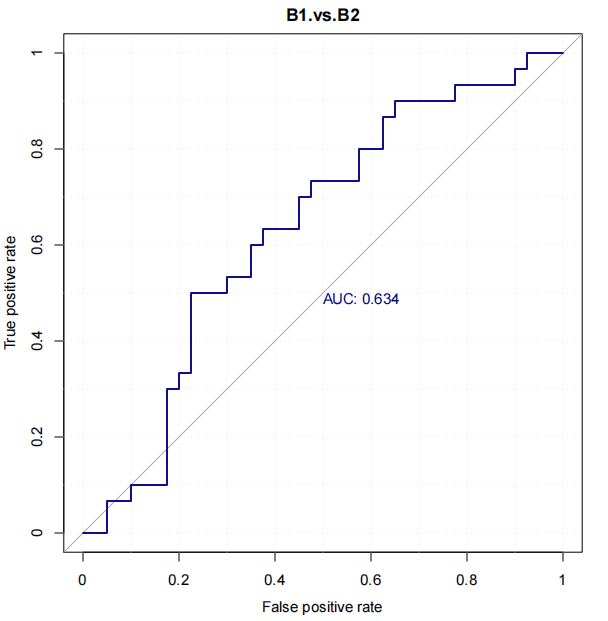


Figure S25: The Discriminatory Ability of Differential Metabolite 7452 between Group B1 and Group B2 under the Positive Ion Mode


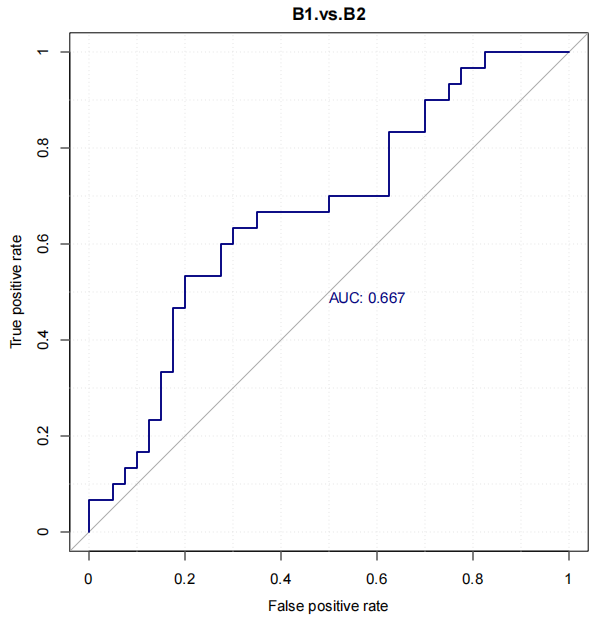


Figure S26: The Discriminatory Ability of Differential Metabolite 9678 between Group B1 and Group B2 under the Positive Ion Mode


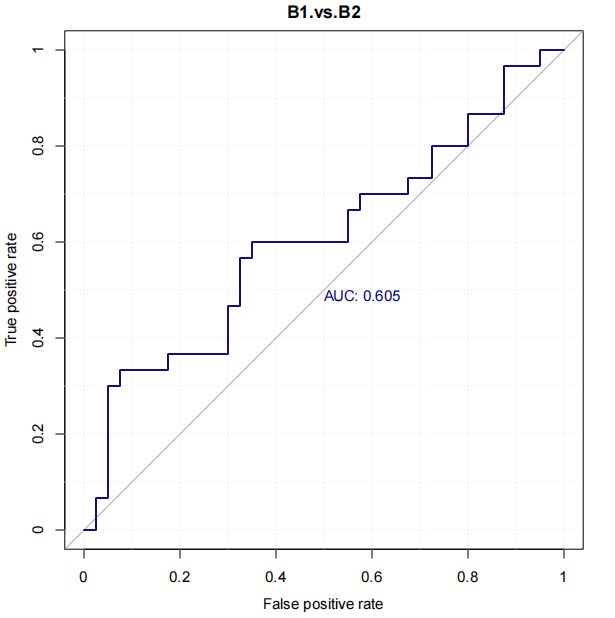


Figure S27: The Discriminatory Ability of Differential Metabolite 9778 between Group B1 and Group B2 under the Positive Ion Mode


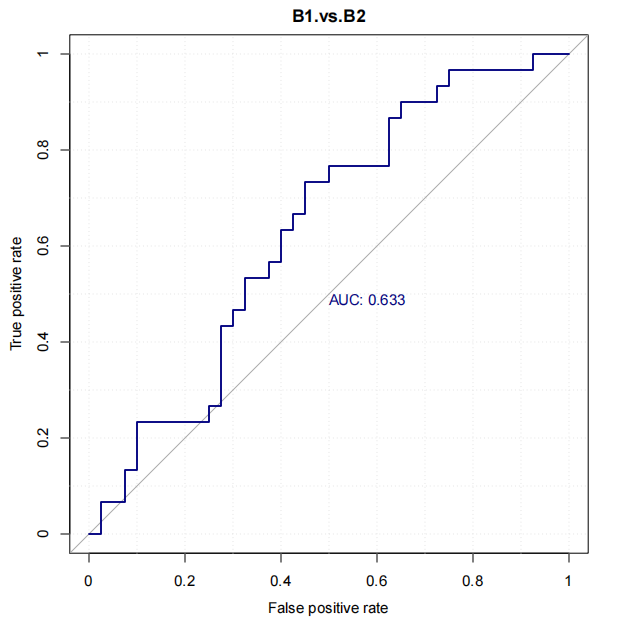


Figure S28: The Discriminatory Ability of Differential Metabolite 10829 between Group B1 and Group B2 under the Positive Ion Mode


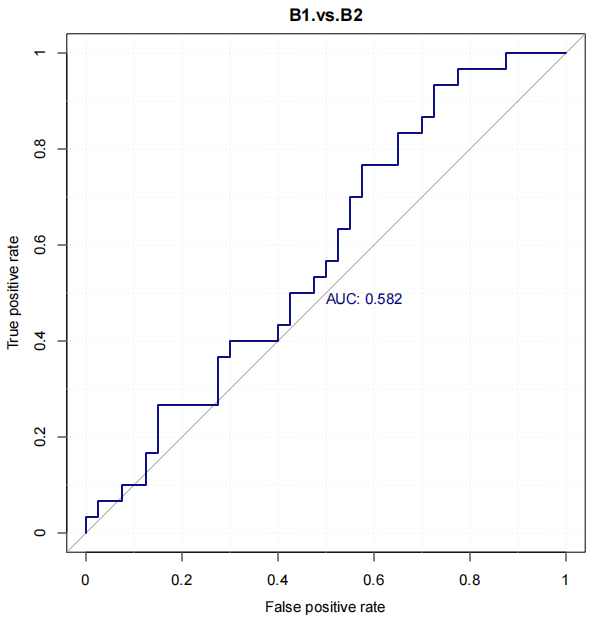


Figure S29: The Discriminatory Ability of Differential Metabolite 11214 between Group B1 and Group B2 under the Positive Ion Mode


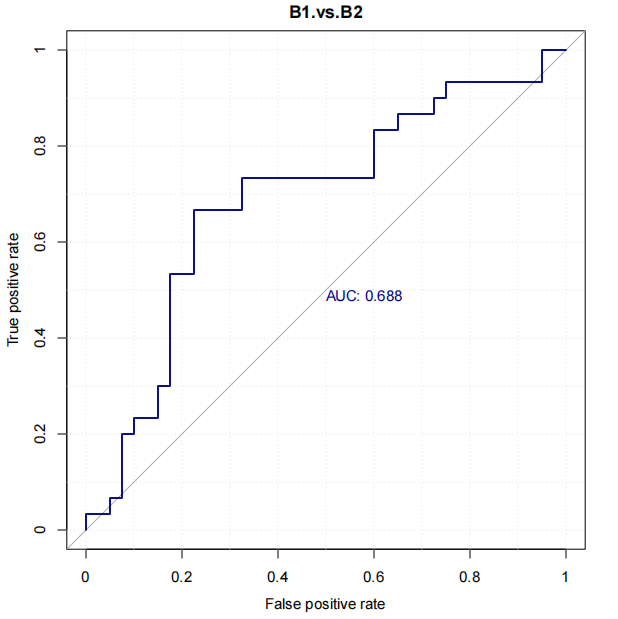


Figure S30: The Discriminatory Ability of Differential Metabolite 12350 between Group B1 and Group B2 under the Positive Ion Mode


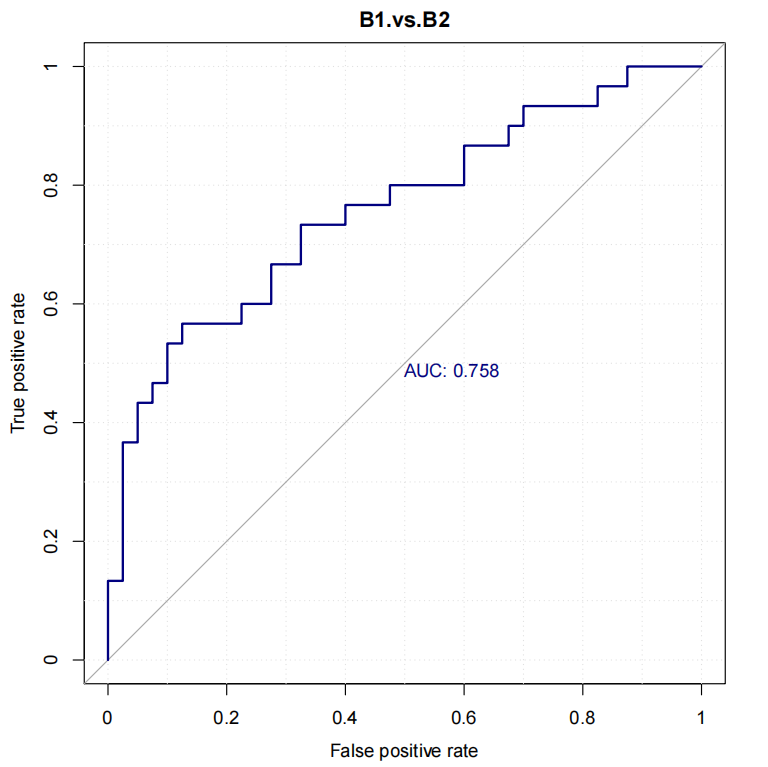


Figure S31: The Discriminatory Ability of Differential Metabolite 13709 between Group B1 and Group B2 under the Positive Ion Mode


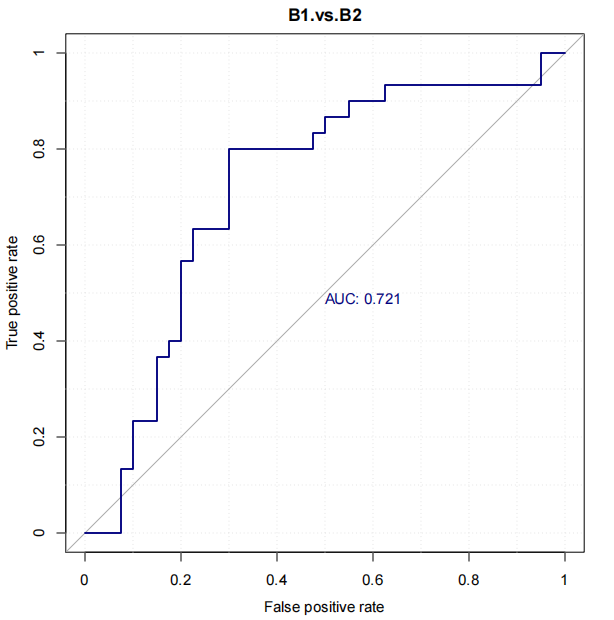


Figure S32: The Discriminatory Ability of Differential Metabolite 14626 between Group B1 and Group B2 under the Positive Ion Mode


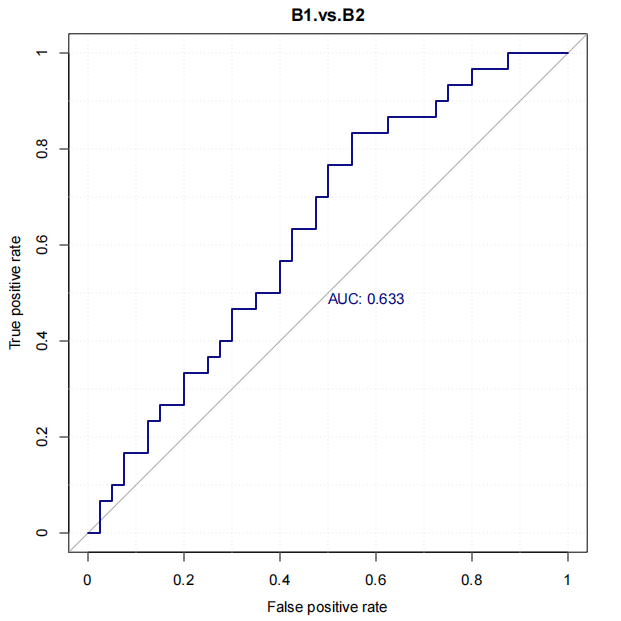


Figure S33: The Discriminatory Ability of Differential Metabolite 14878 between Group B1 and Group B2 under the Positive Ion Mode


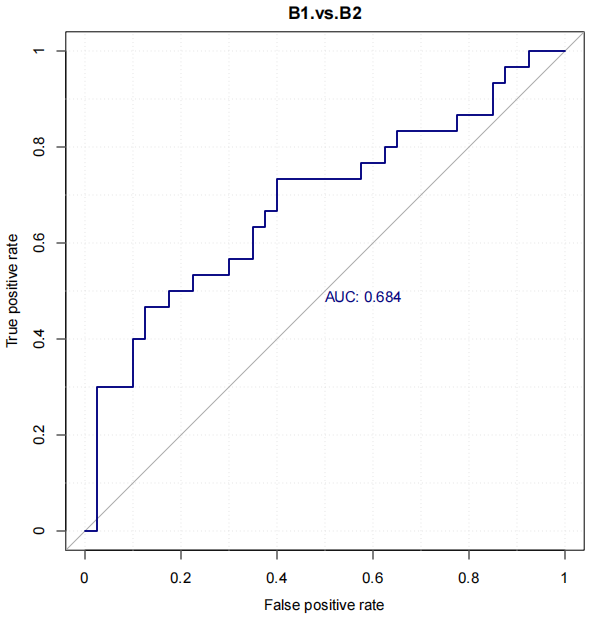


Figure S34: The Discriminatory Ability of Differential Metabolite 18420 between Group B1 and Group B2 under the Positive Ion Mode


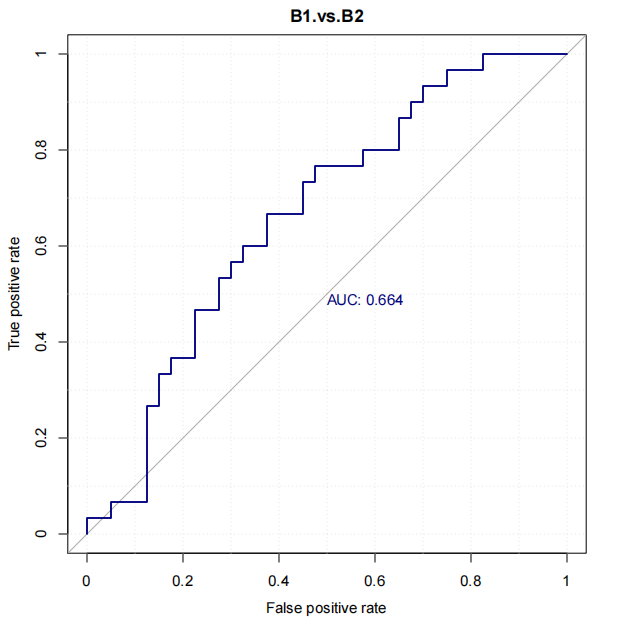


Figure S35: The Discriminatory Ability of Differential Metabolite 22379 between Group B1 and Group B2 under the Positive Ion Mode


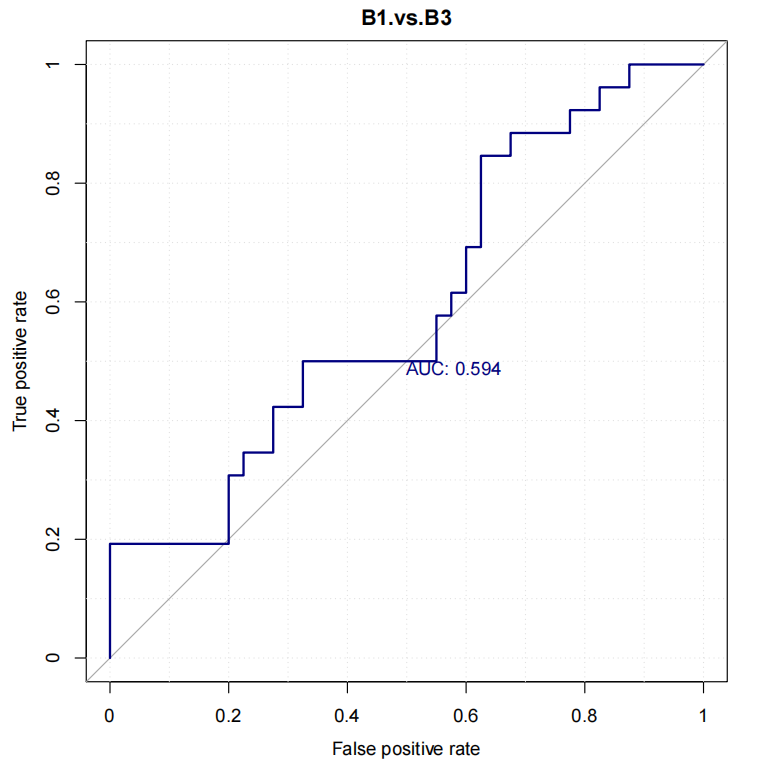


Figure S36: The Discriminatory Ability of Differential Metabolite 7 between Group B1 and Group B3 under the Negative Ion Mode


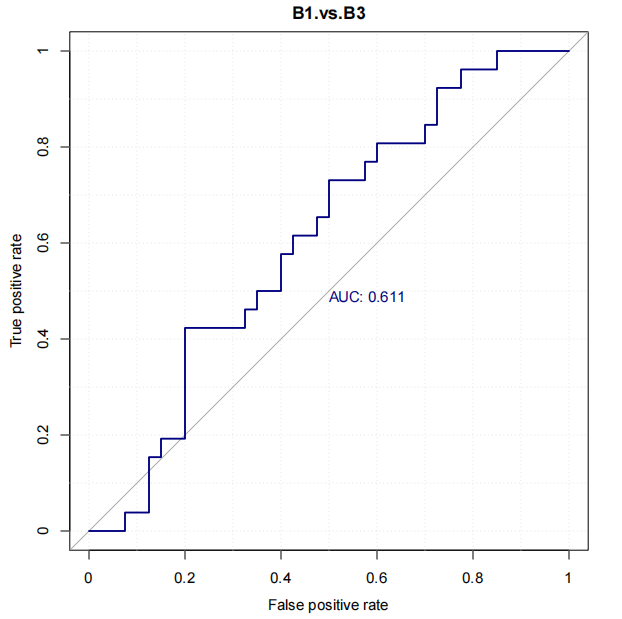


Figure S37: The Discriminatory Ability of Differential Metabolite 455 between Group B1 and Group B3 under the Negative Ion Mode


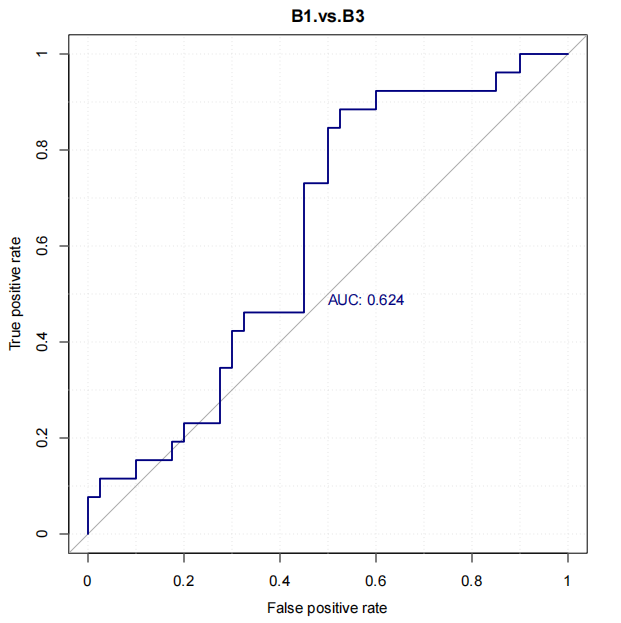


Figure S38: The Discriminatory Ability of Differential Metabolite 1711 between Group B1 and Group B3 under the Negative Ion Mode


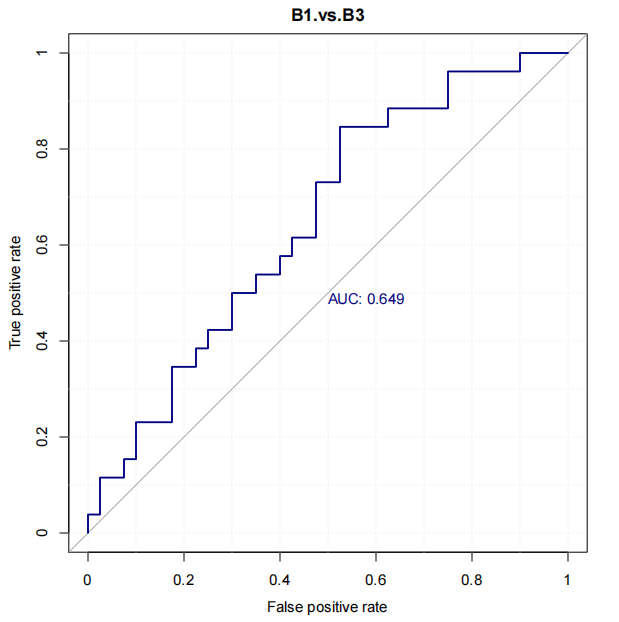


Figure S39: The Discriminatory Ability of Differential Metabolite 1754 between Group B1 and Group B3 under the Negative Ion Mode


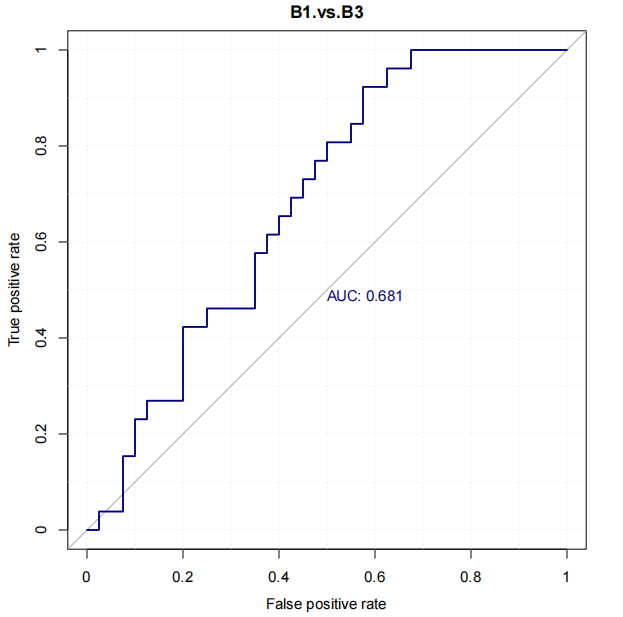


Figure S40: The Discriminatory Ability of Differential Metabolite 2044 between Group B1 and Group B3 under the Negative Ion Mode


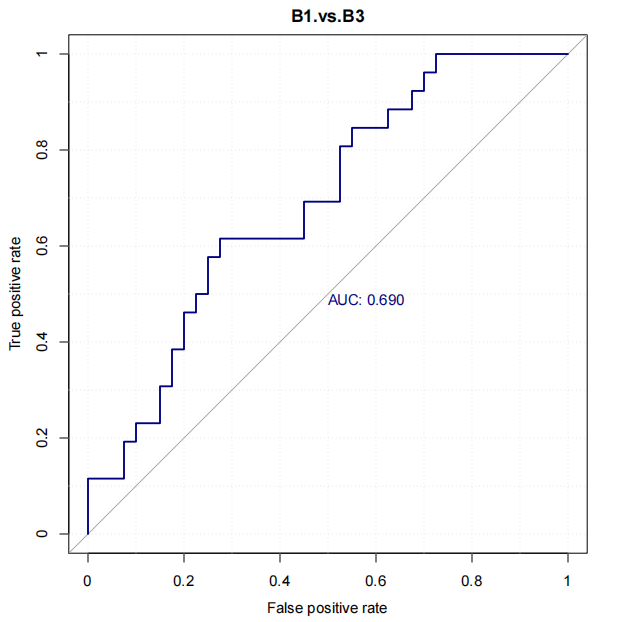


Figure S41: The Discriminatory Ability of Differential Metabolite 2430 between Group B1 and Group B3 under the Negative Ion Mode


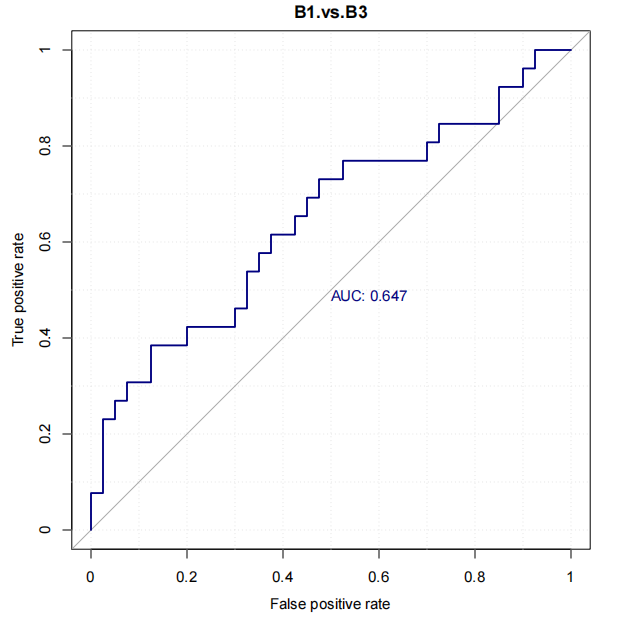


Figure S42: The Discriminatory Ability of Differential Metabolite 2693 between Group B1 and Group B3 under the Negative Ion Mode


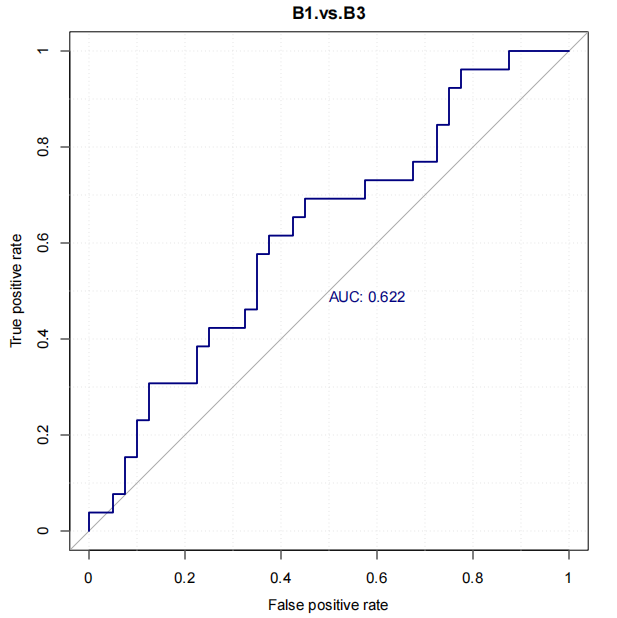


Figure S43: The Discriminatory Ability of Differential Metabolite 3337 between Group B1 and Group B3 under the Negative Ion Mode


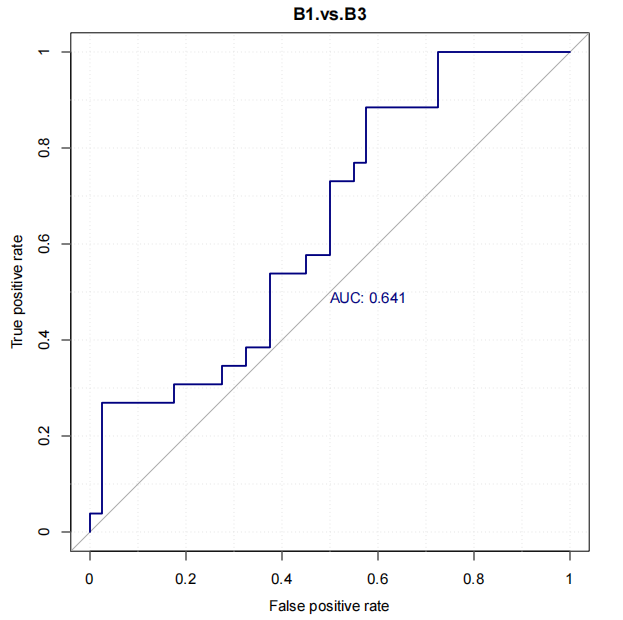


Figure S44: The Discriminatory Ability of Differential Metabolite 7348 between Group B1 and Group B3 under the Negative Ion Mode


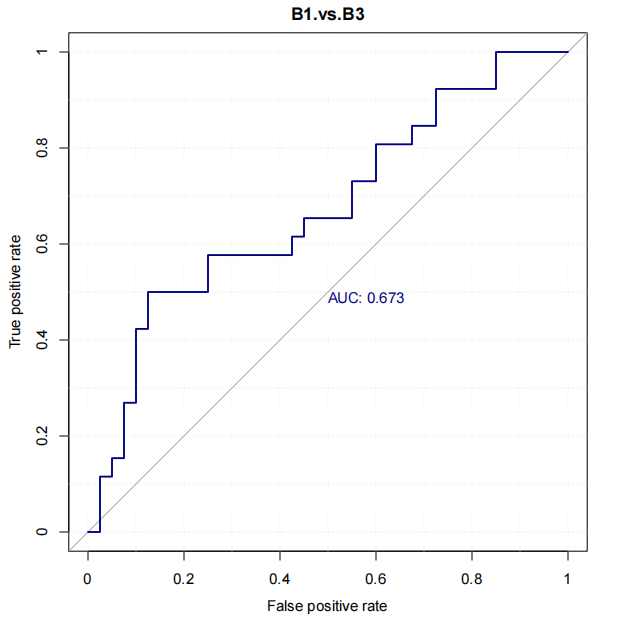


Figure S45: The Discriminatory Ability of Differential Metabolite 7485 between Group B1 and Group B3 under the Negative Ion Mode


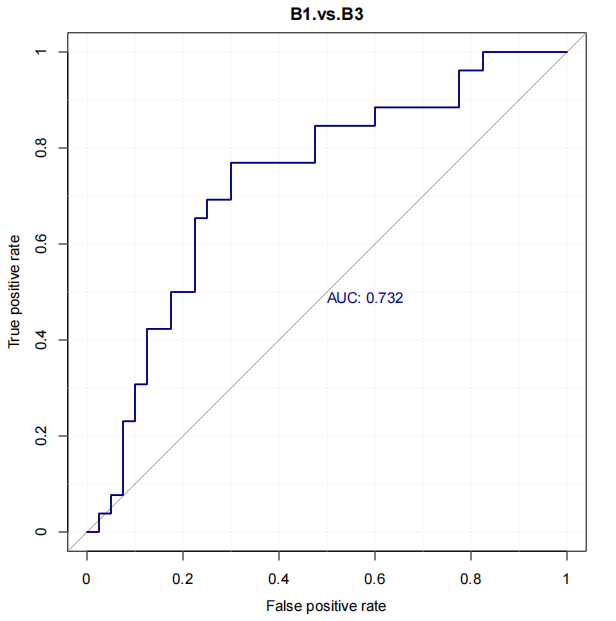


Figure S46: The Discriminatory Ability of Differential Metabolite 11 between Group B1 and Group B3 under the Negative Ion Mode


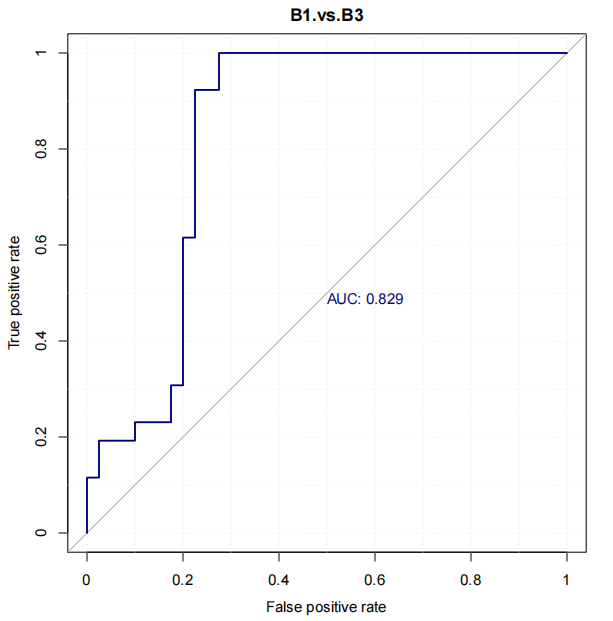


Figure S47: The Discriminatory Ability of Differential Metabolite 133 between Group B1 and Group B3 under the Negative Ion Mode


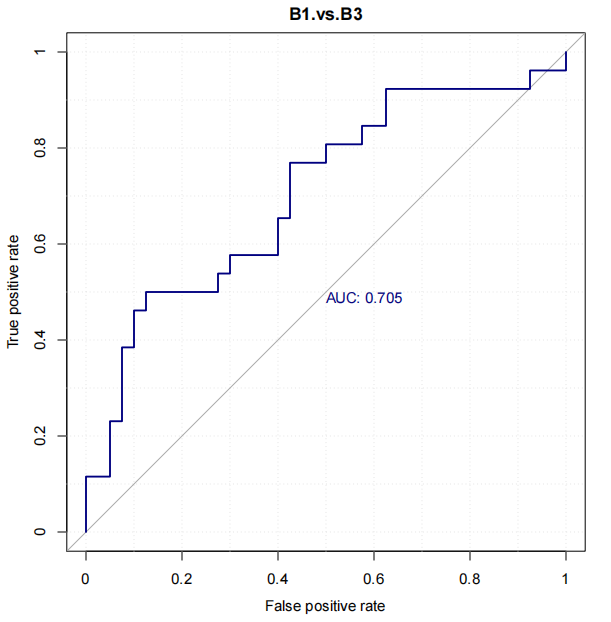


Figure S48: The Discriminatory Ability of Differential Metabolite 1499 between Group B1 and Group B3 under the Negative Ion Mode


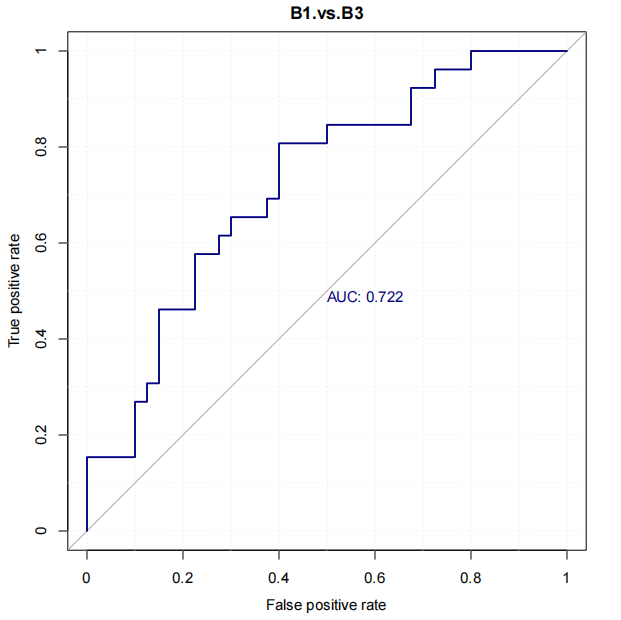


Figure S49: The Discriminatory Ability of Differential Metabolite 1741 between Group B1 and Group B3 under the Negative Ion Mode


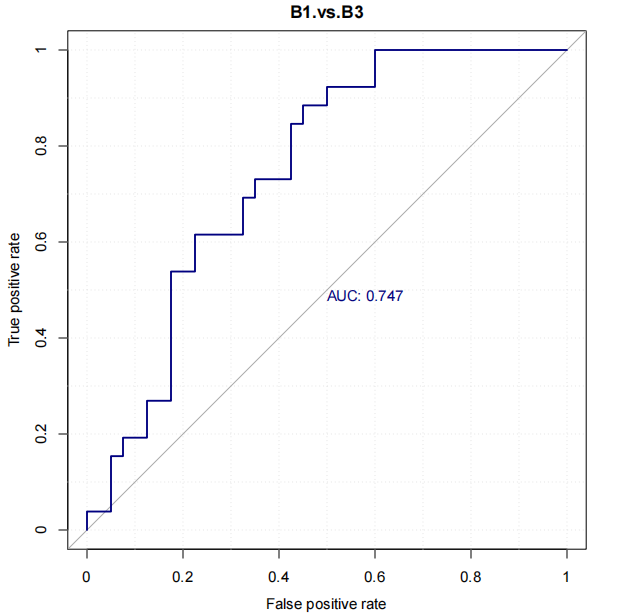


Figure S50: The Discriminatory Ability of Differential Metabolite 1757 between Group B1 and Group B3 under the Negative Ion Mode


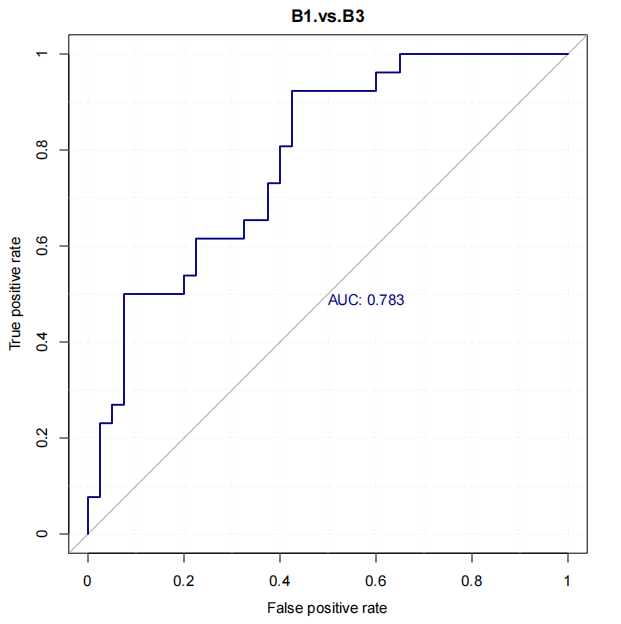


Figure S51: The Discriminatory Ability of Differential Metabolite 1988 between Group B1 and Group B3 under the Negative Ion Mode


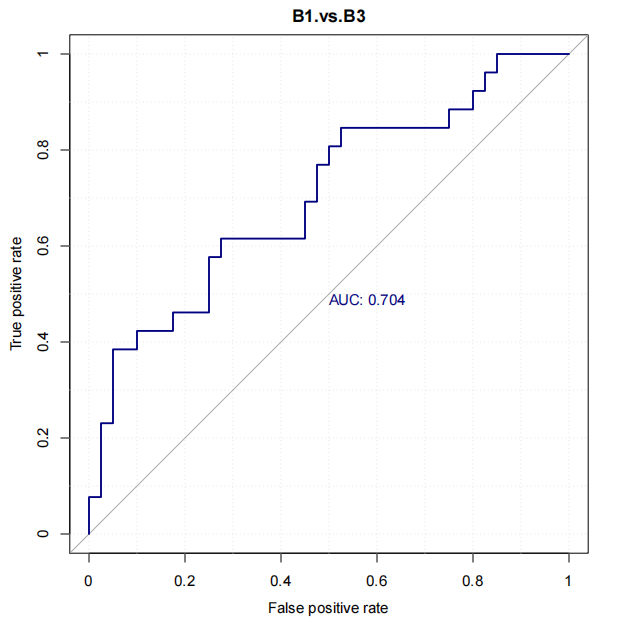


Figure S52: The Discriminatory Ability of Differential Metabolite 4051 between Group B1 and Group B3 under the Negative Ion Mode


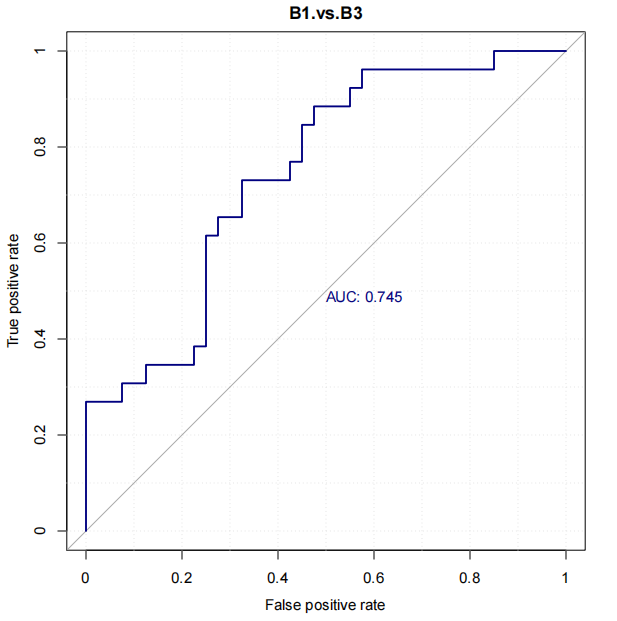


Figure S53: The Discriminatory Ability of Differential Metabolite 5124 between Group B1 and Group B3 under the Negative Ion Mode


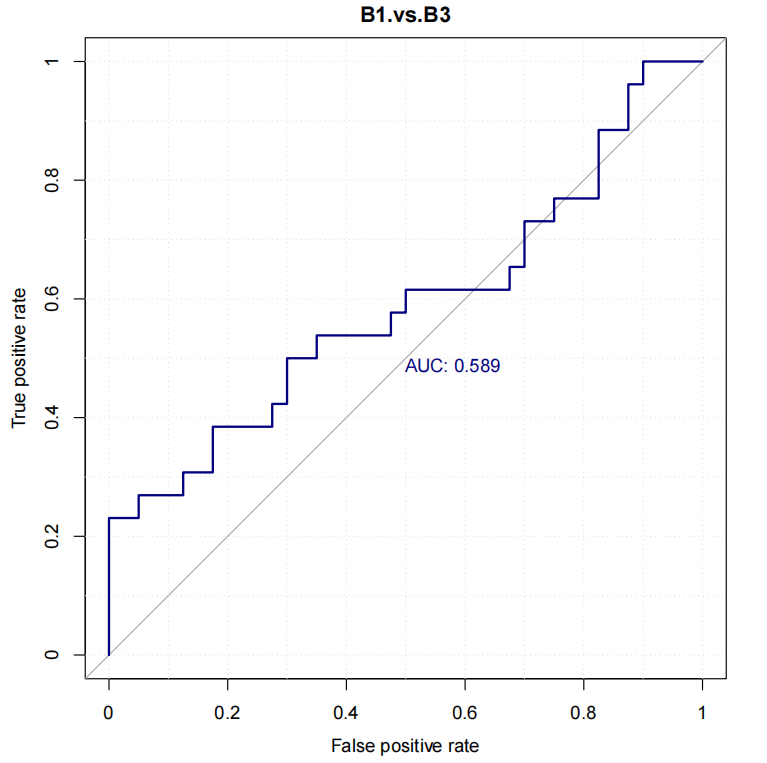


Figure S54: The Discriminatory Ability of Differential Metabolite 661 between Group B1 and Group B3 under the Positive Ion Mode


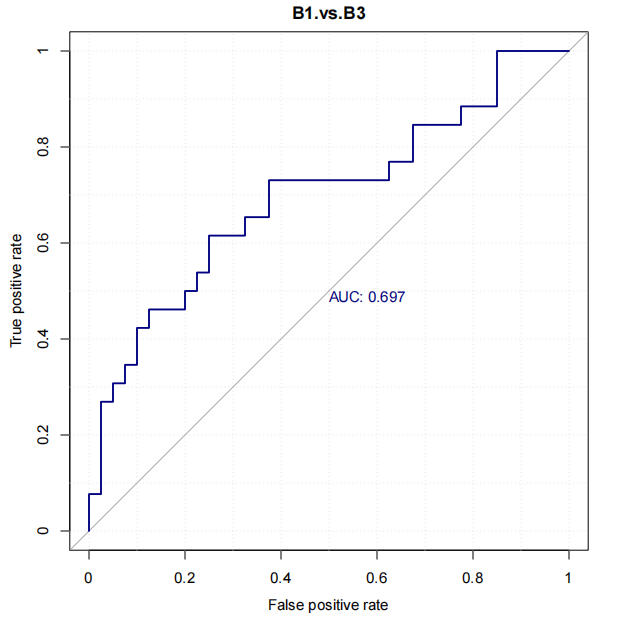


Figure S55: The Discriminatory Ability of Differential Metabolite 1044 between Group B1 and Group B3 under the Positive Ion Mode


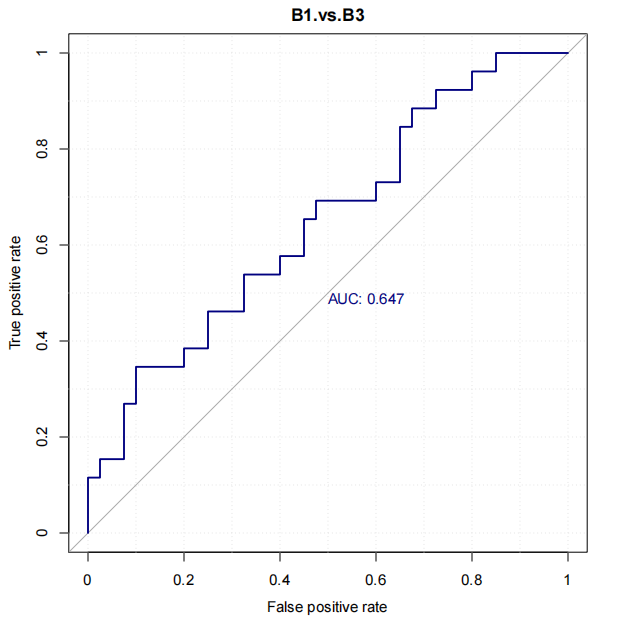


Figure S56: The Discriminatory Ability of Differential Metabolite 3854 between Group B1 and Group B3 under the Positive Ion Mode


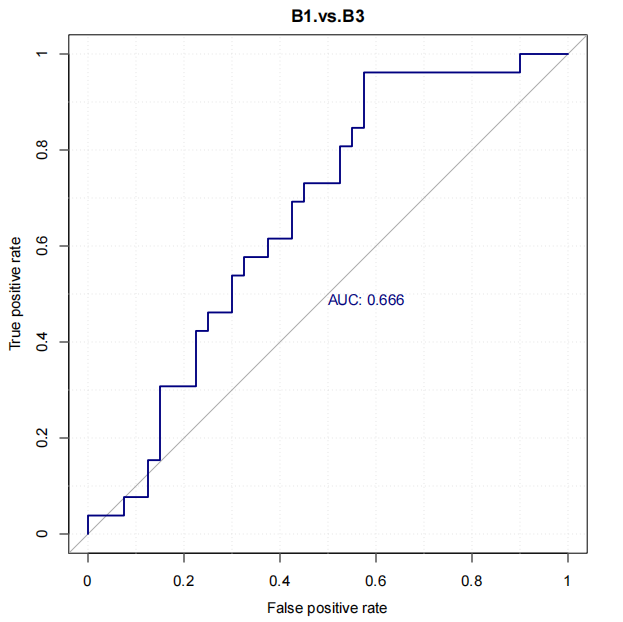


Figure S57: The Discriminatory Ability of Differential Metabolite 4053 between Group B1 and Group B3 under the Positive Ion Mode


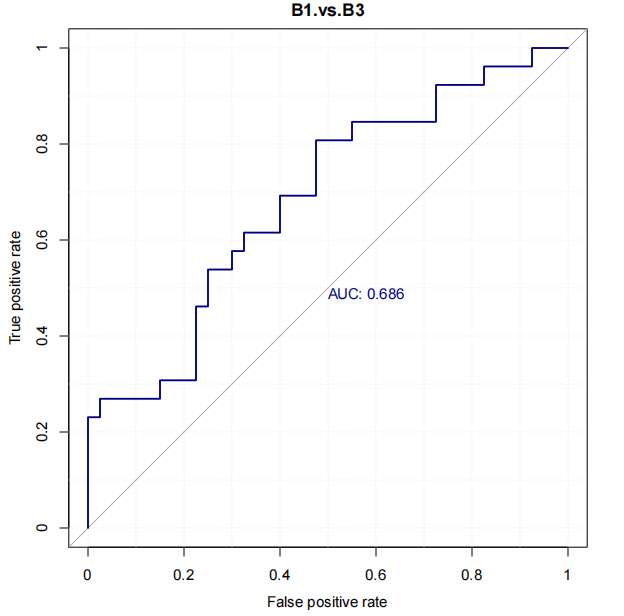


Figure S58: The Discriminatory Ability of Differential Metabolite 4596 between Group B1 and Group B3 under the Positive Ion Mode


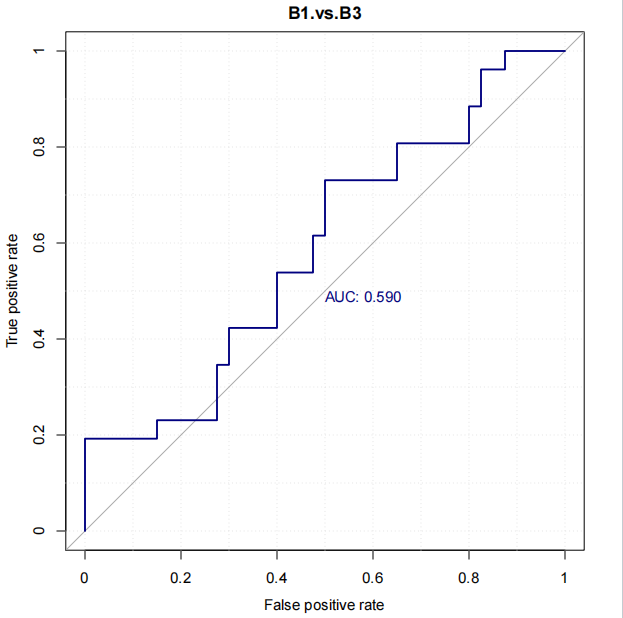


Figure S59: The Discriminatory Ability of Differential Metabolite 6226 between Group B1 and Group B3 under the Positive Ion Mode


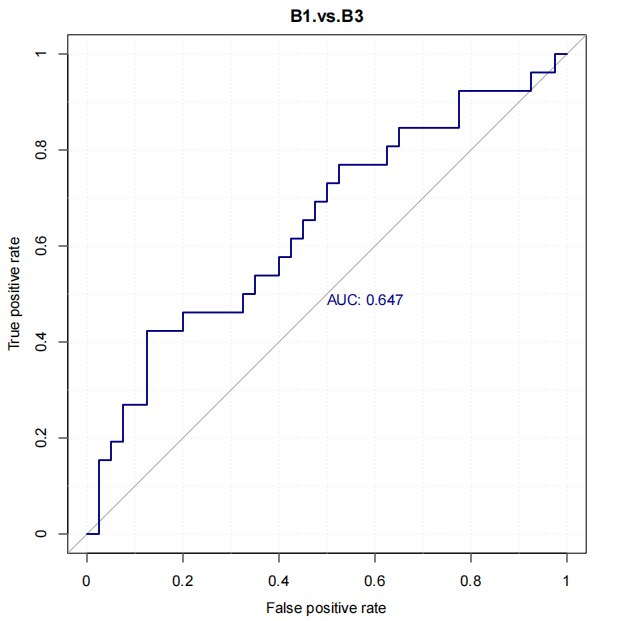


Figure S60: The Discriminatory Ability of Differential Metabolite 6984 between Group B1 and Group B3 under the Positive Ion Mode


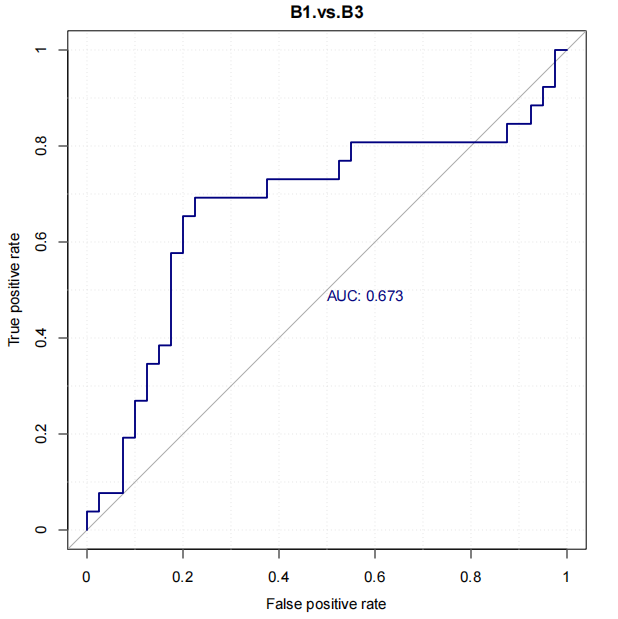


Figure S61: The Discriminatory Ability of Differential Metabolite 8147 between Group B1 and Group B3 under the Positive Ion Mode


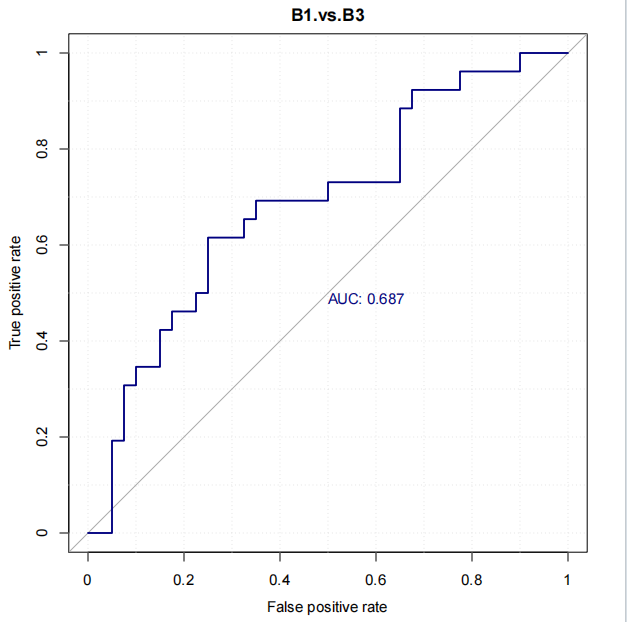


Figure S62: The Discriminatory Ability of Differential Metabolite 8491 between Group B1 and Group B3 under the Positive Ion Mode


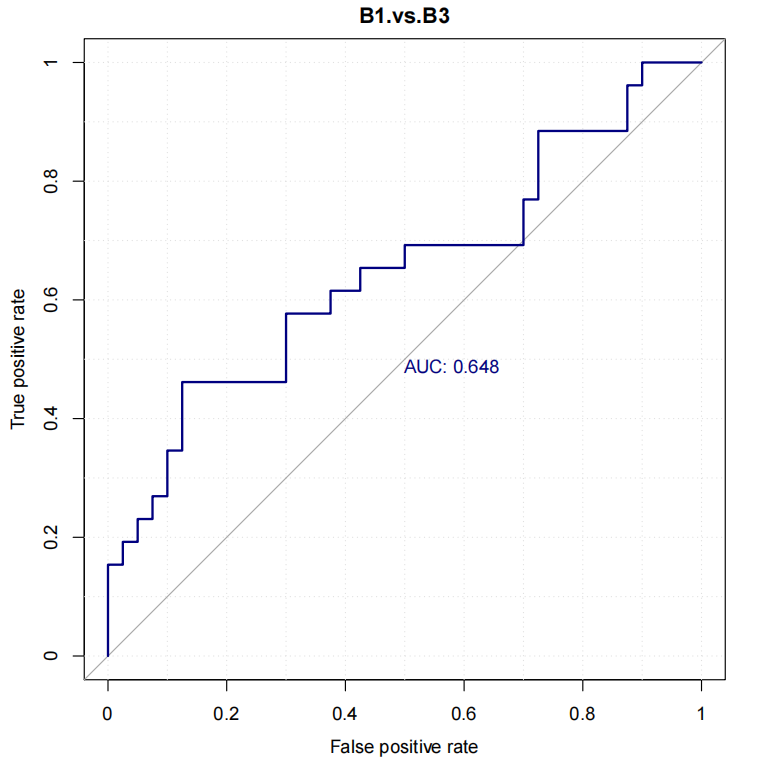


Figure S63: The Discriminatory Ability of Differential Metabolite 8910 between Group B1 and Group B3 under the Positive Ion Mode


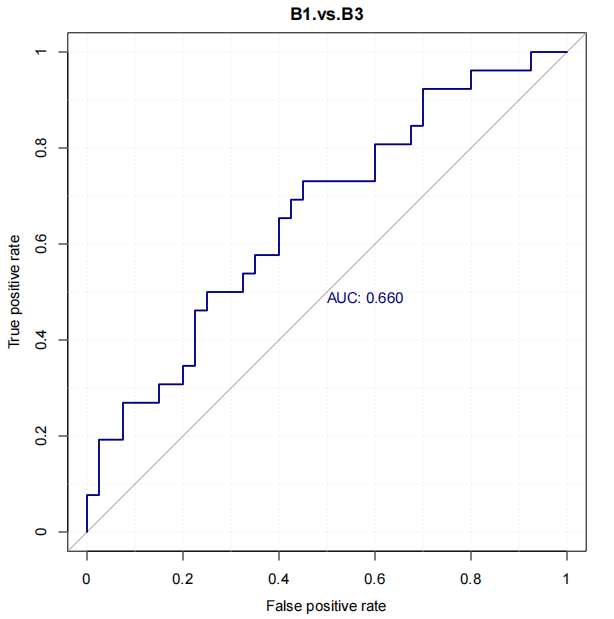


Figure S64: The Discriminatory Ability of Differential Metabolite 9530 between Group B1 and Group B3 under the Positive Ion Mode


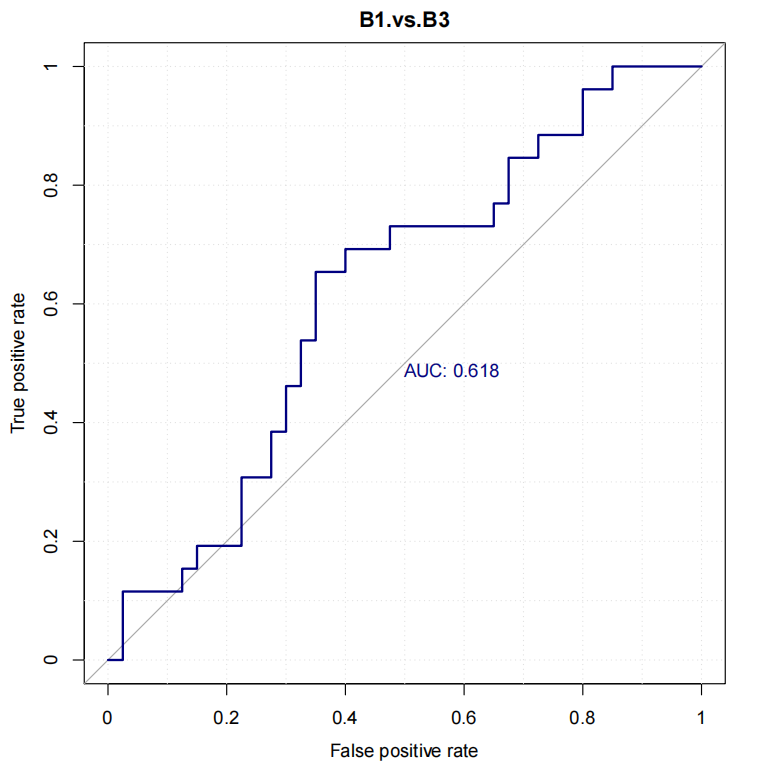


Figure S65: The Discriminatory Ability of Differential Metabolite 10072 between Group B1 and Group B3 under the Positive Ion Mode


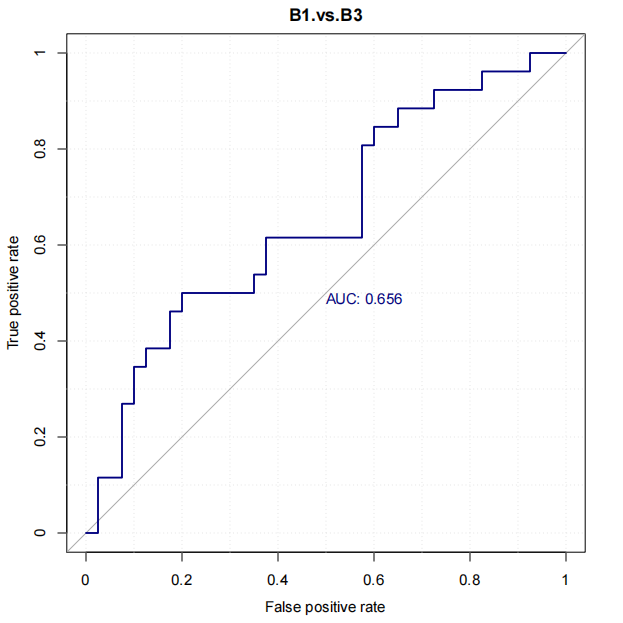


Figure S66: The Discriminatory Ability of Differential Metabolite 10290 between Group B1 and Group B3 under the Positive Ion Mode


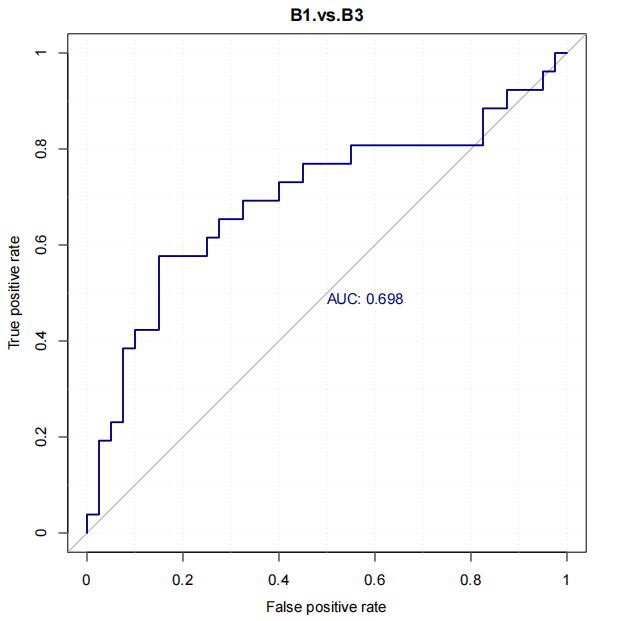


Figure S67: The Discriminatory Ability of Differential Metabolite 10739 between Group B1 and Group B3 under the Positive Ion Mode


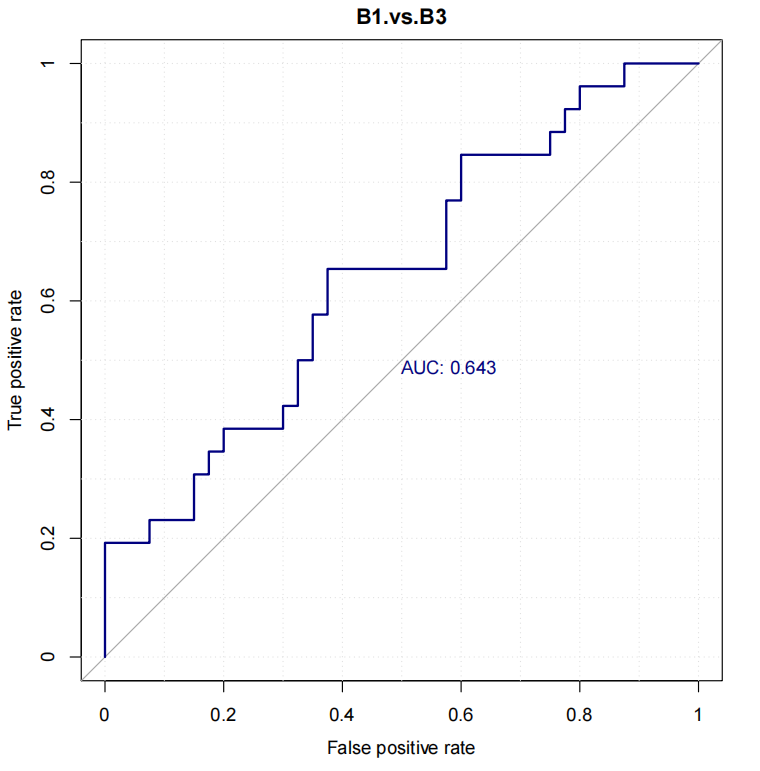


Figure S68: The Discriminatory Ability of Differential Metabolite 10916 between Group B1 and Group B3 under the Positive Ion Mode


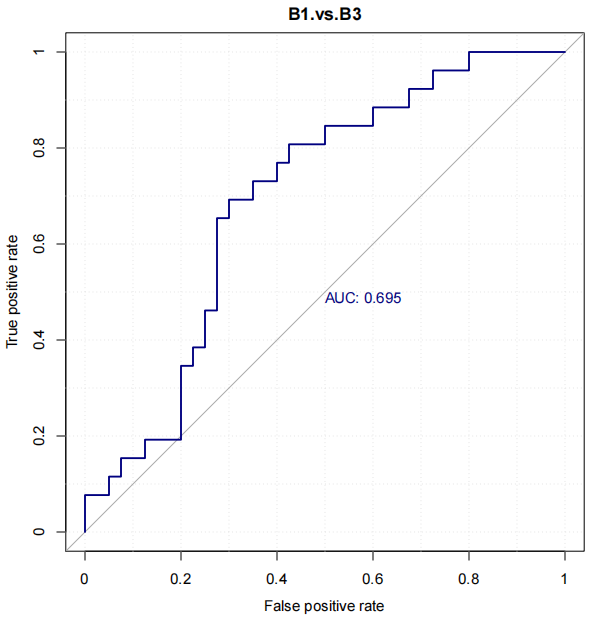


Figure S69: The Discriminatory Ability of Differential Metabolite 12372 between Group B1 and Group B3 under the Positive Ion Mode


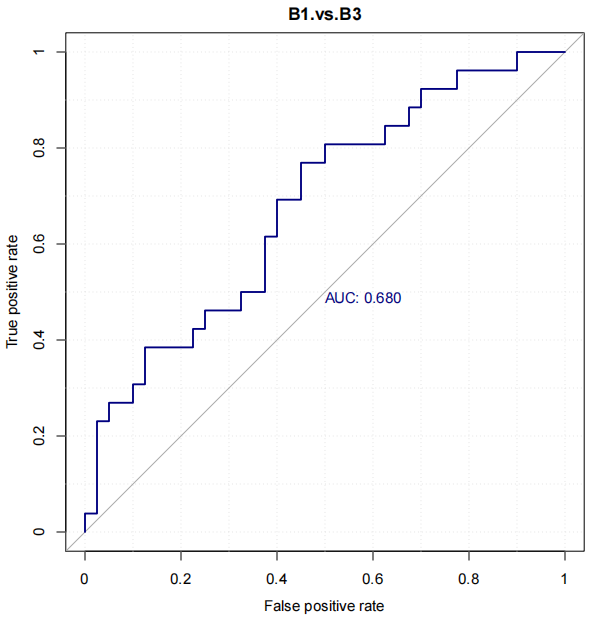


Figure S70: The Discriminatory Ability of Differential Metabolite 12462 between Group B1 and Group B3 under the Positive Ion Mode


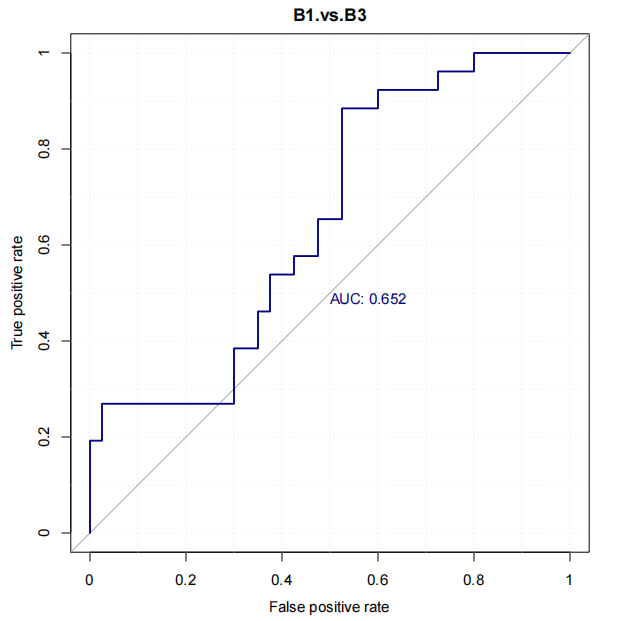


Figure S71: The Discriminatory Ability of Differential Metabolite 12812 between Group B1 and Group B3 under the Positive Ion Mode


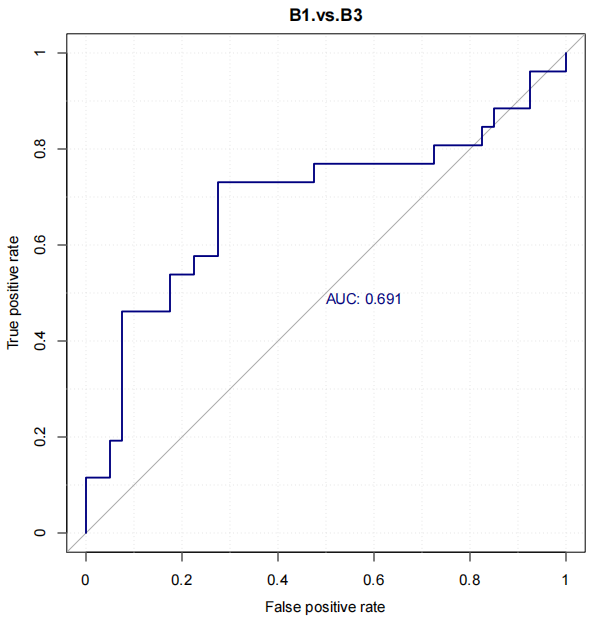


Figure S72: The Discriminatory Ability of Differential Metabolite 13309 between Group B1 and Group B3 under the Positive Ion Mode


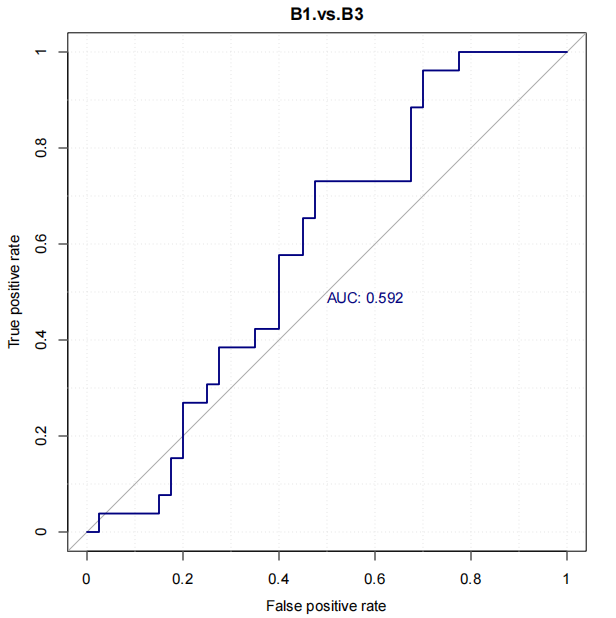


Figure S73: The Discriminatory Ability of Differential Metabolite 13550 between Group B1 and Group B3 under the Positive Ion Mode


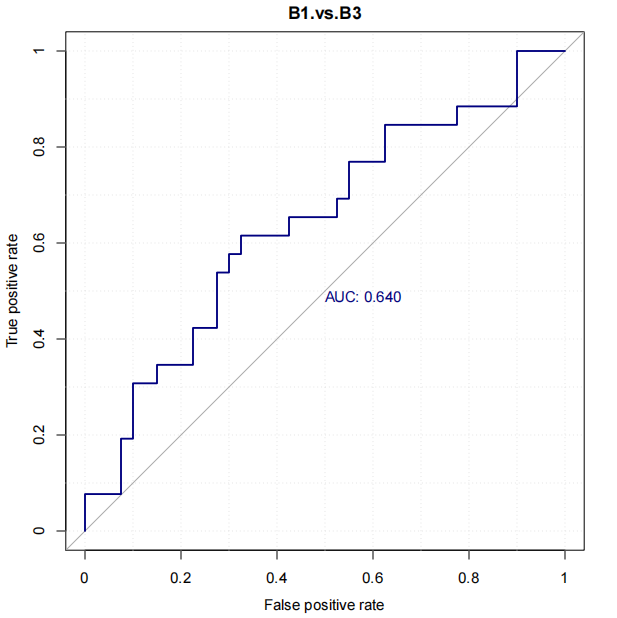


Figure S74: The Discriminatory Ability of Differential Metabolite 14476 between Group B1 and Group B3 under the Positive Ion Mode


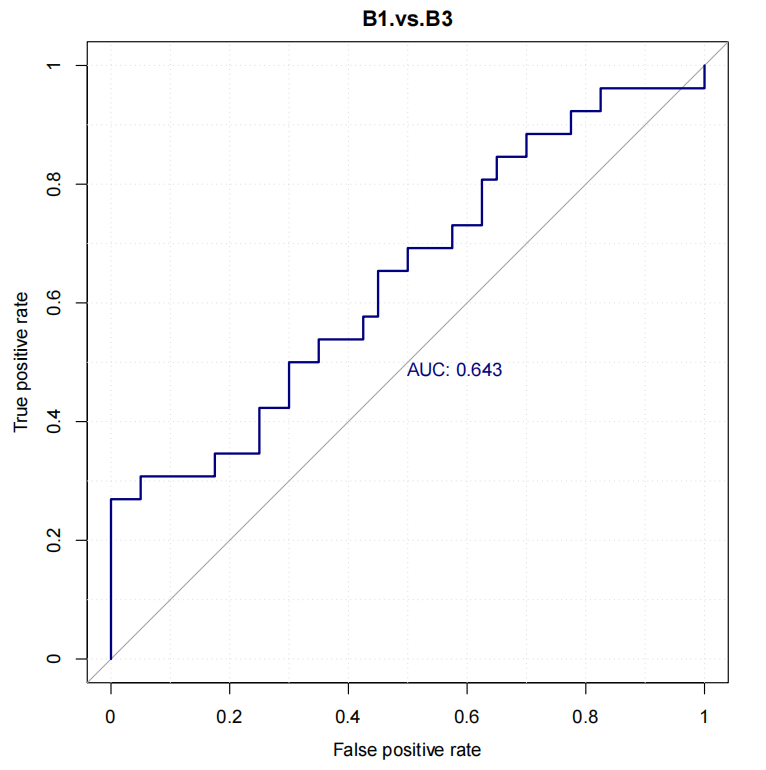


Figure S75: The Discriminatory Ability of Differential Metabolite 14679 between Group B1 and Group B3 under the Positive Ion Mode


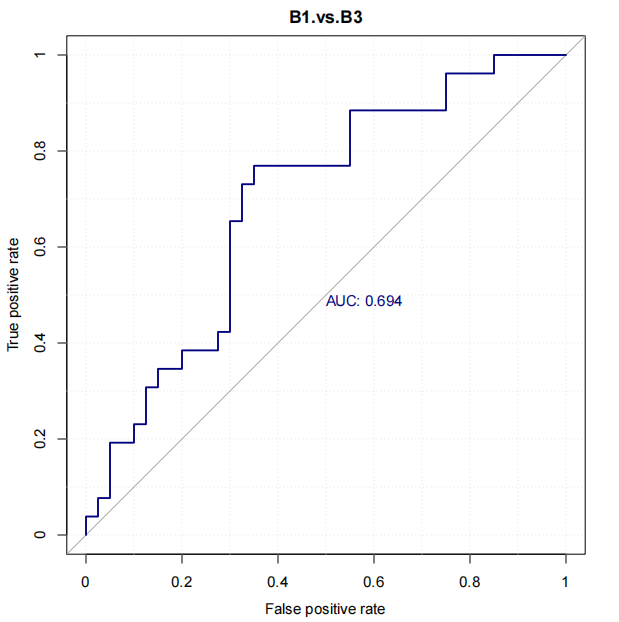


Figure S76: The Discriminatory Ability of Differential Metabolite 15197 between Group B1 and Group B3 under the Positive Ion Mode


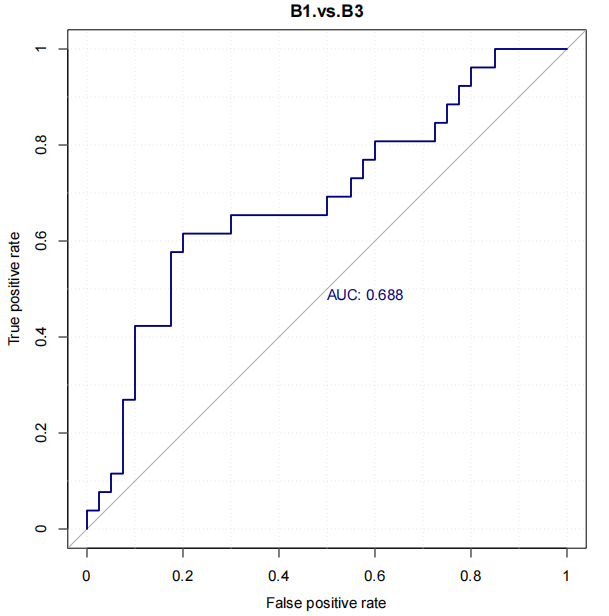


Figure S77: The Discriminatory Ability of Differential Metabolite 17163 between Group B1 and Group B3 under the Positive Ion Mode


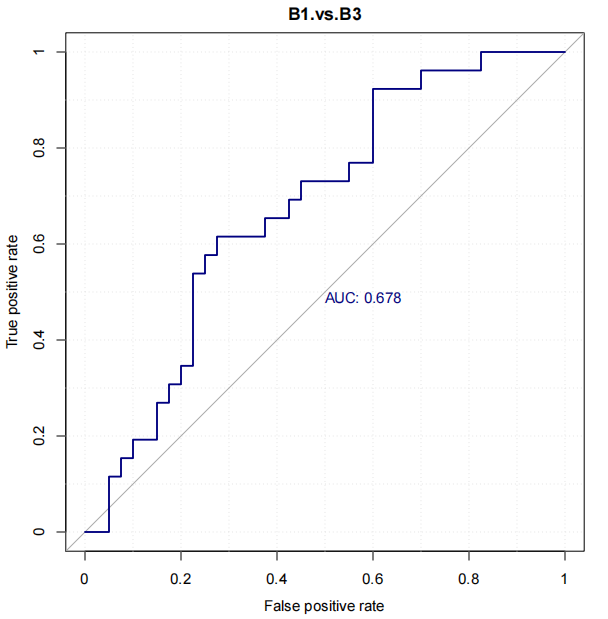


Figure S78: The Discriminatory Ability of Differential Metabolite 17785 between Group B1 and Group B3 under the Positive Ion Mode


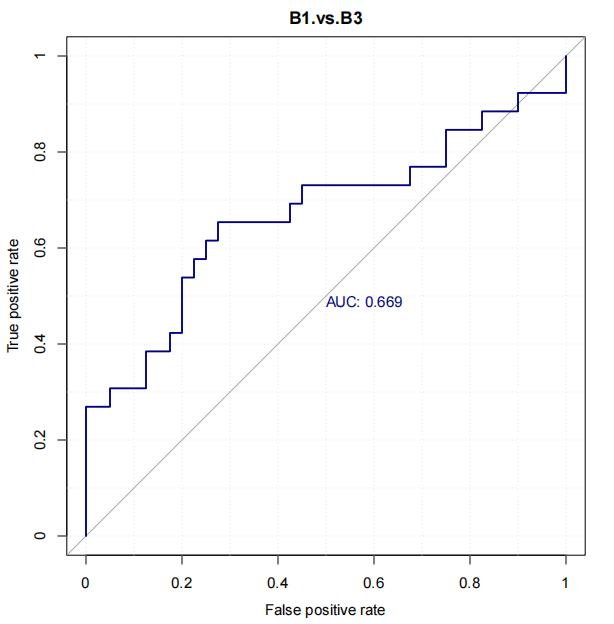


Figure S79: The Discriminatory Ability of Differential Metabolite 19917 between Group B1 and Group B3 under the Positive Ion Mode


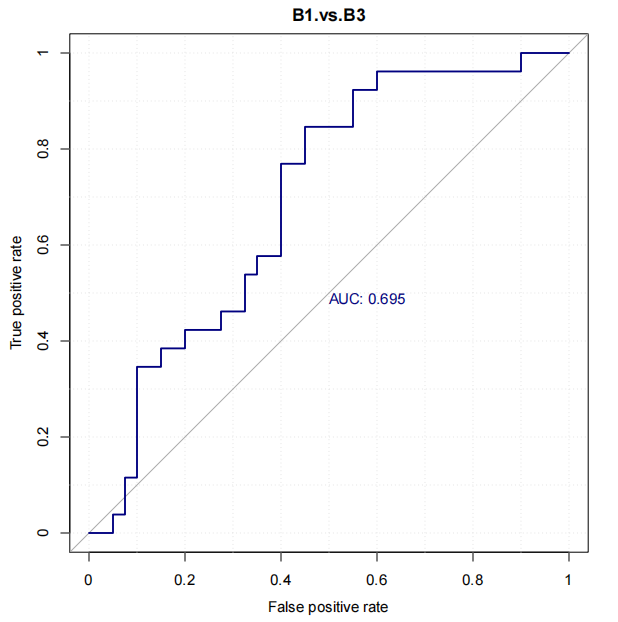


Figure S80: The Discriminatory Ability of Differential Metabolite 20761 between Group B1 and Group B3 under the Positive Ion Mode


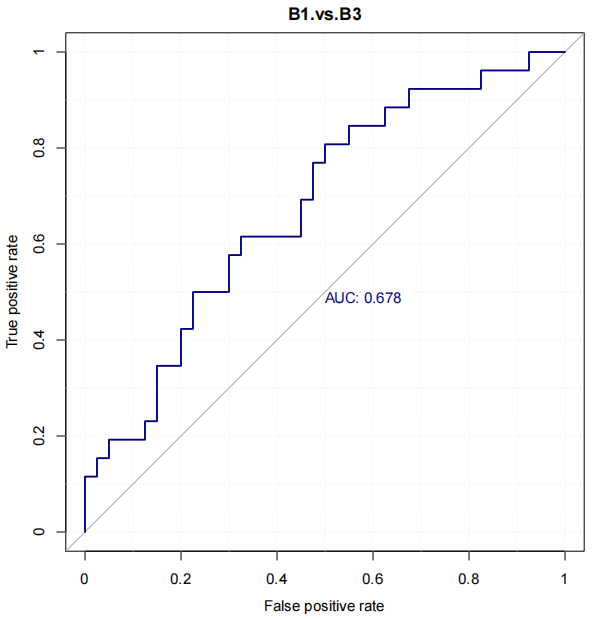


Figure S81: The Discriminatory Ability of Differential Metabolite 22940 between Group B1 and Group B3 under the Positive Ion Mode


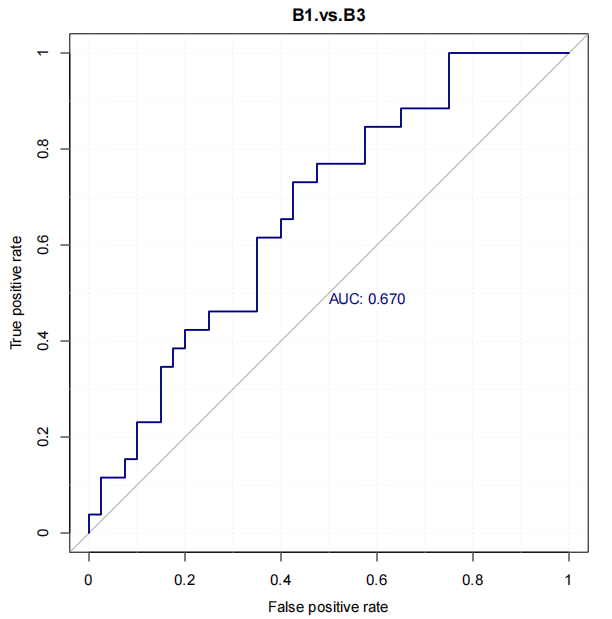


Figure S82: The Discriminatory Ability of Differential Metabolite 23402 between Group B1 and Group B3 under the Positive Ion Mode


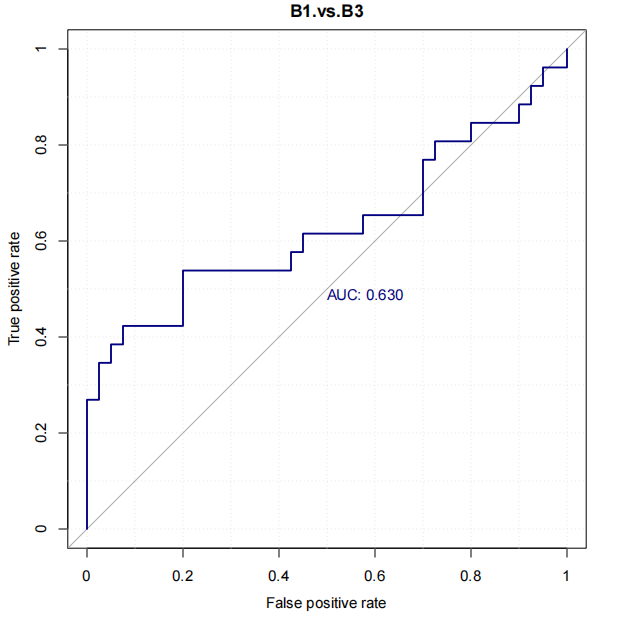


Figure S83: The Discriminatory Ability of Differential Metabolite 23998 between Group B1 and Group B3 under the Positive Ion Mode


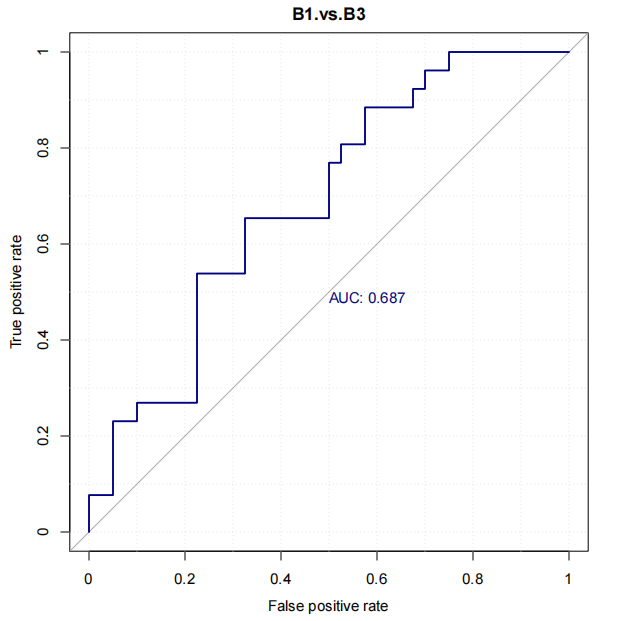


Figure S84: The Discriminatory Ability of Differential Metabolite 25597 between Group B1 and Group B3 under the Positive Ion Mode


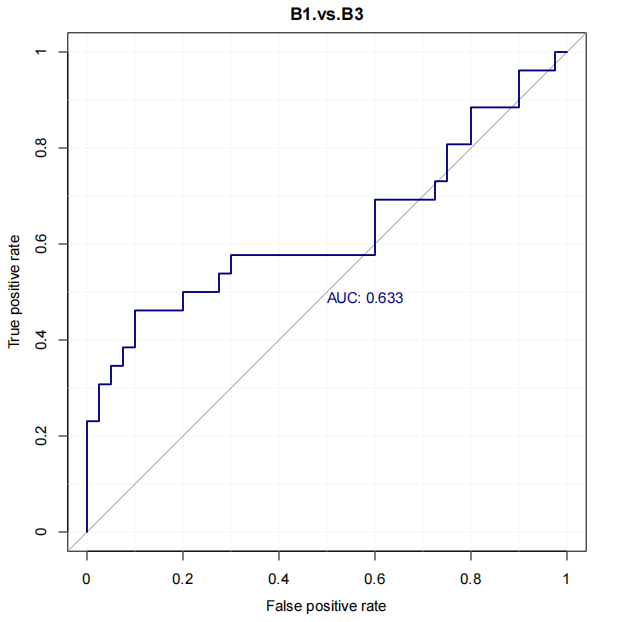


Figure S85: The Discriminatory Ability of Differential Metabolite 26829 between Group B1 and Group B3 under the Positive Ion Mode


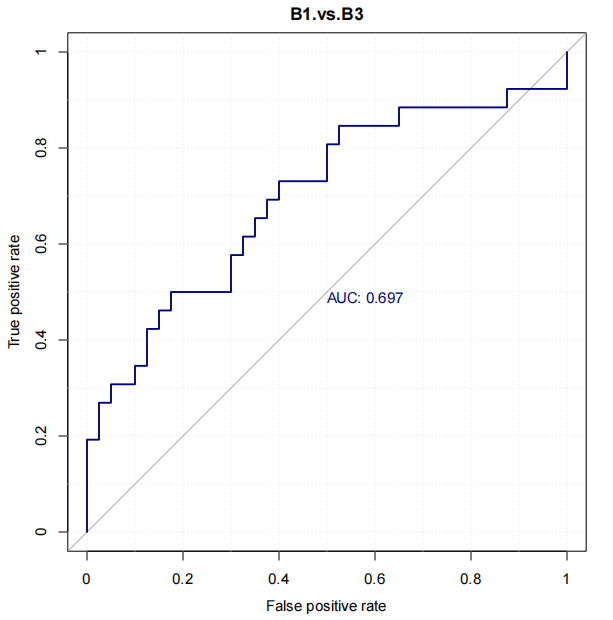


Figure S86: The Discriminatory Ability of Differential Metabolite 27480 between Group B1 and Group B3 under the Positive Ion Mode


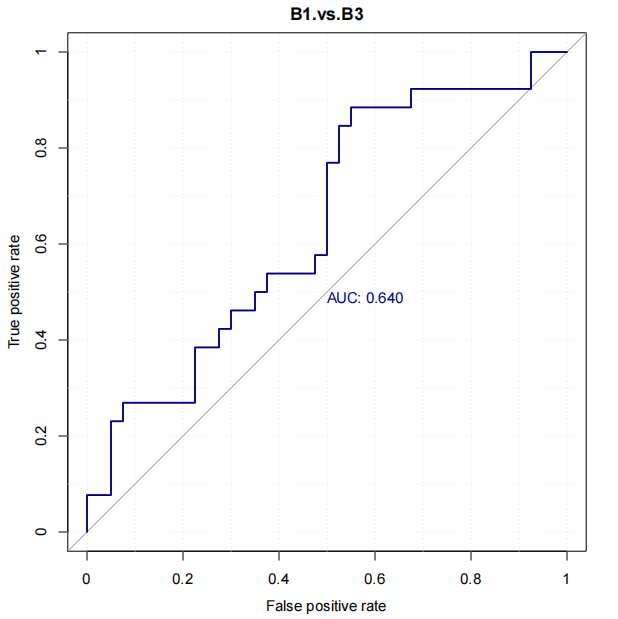


Figure S87: The Discriminatory Ability of Differential Metabolite 27486 between Group B1 and Group B3 under the Positive Ion Mode


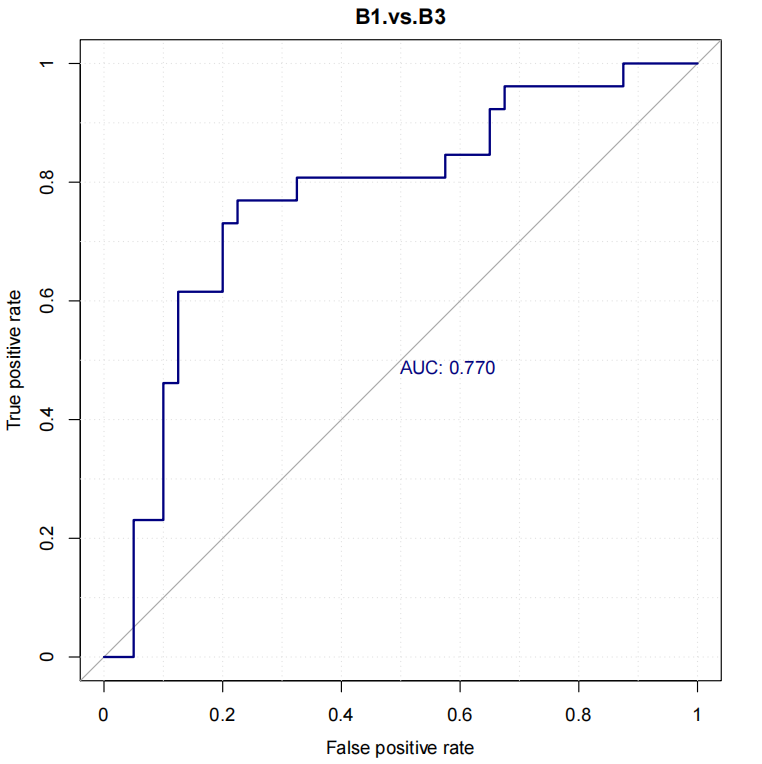


Figure S88: The Discriminatory Ability of Differential Metabolite 6 between Group B1 and Group B3 under the Positive Ion Mode


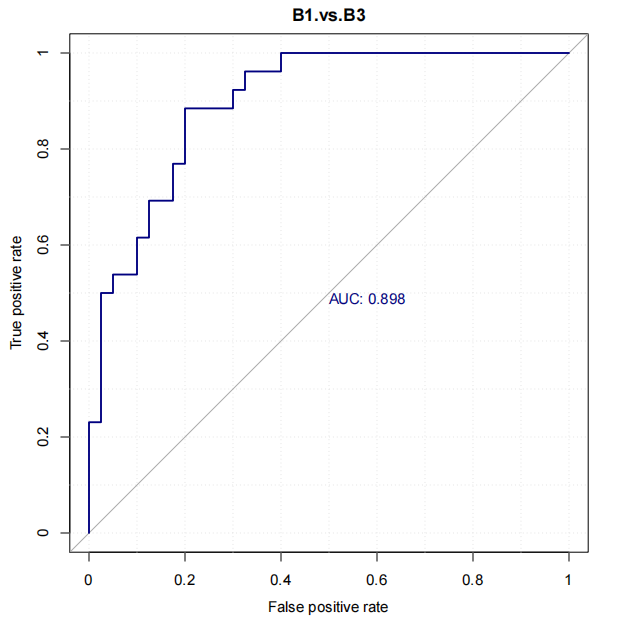


Figure S89: The Discriminatory Ability of Differential Metabolite 16 between Group B1 and Group B3 under the Positive Ion Mode


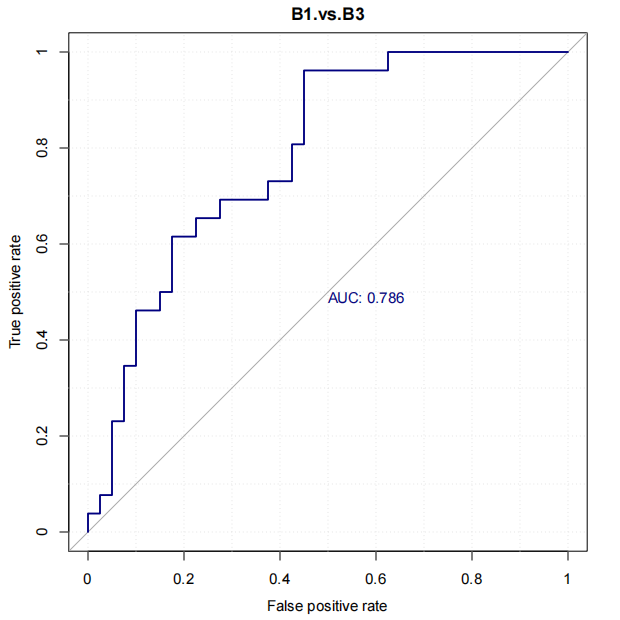


Figure S90: The Discriminatory Ability of Differential Metabolite 27 between Group B1 and Group B3 under the Positive Ion Mode


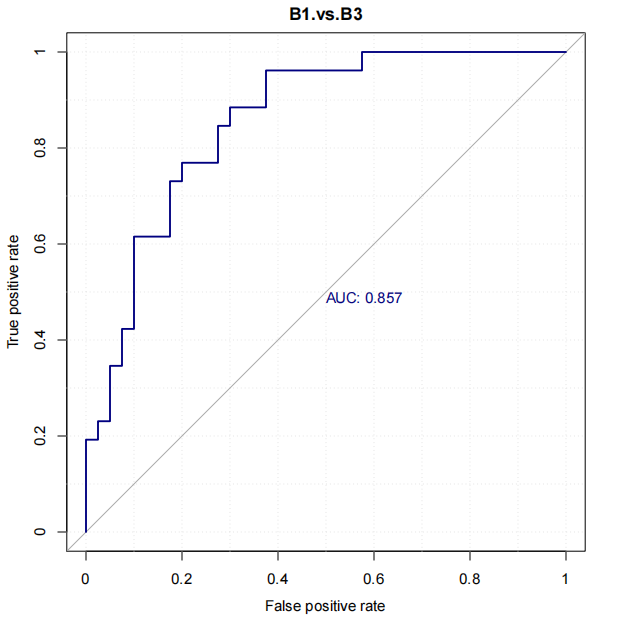


Figure S91: The Discriminatory Ability of Differential Metabolite 434 between Group B1 and Group B3 under the Positive Ion Mode


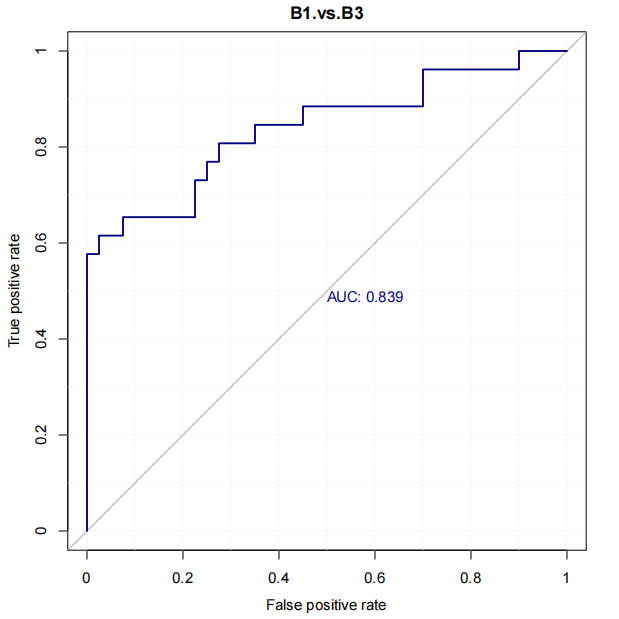


Figure S92: The Discriminatory Ability of Differential Metabolite 526 between Group B1 and Group B3 under the Positive Ion Mode


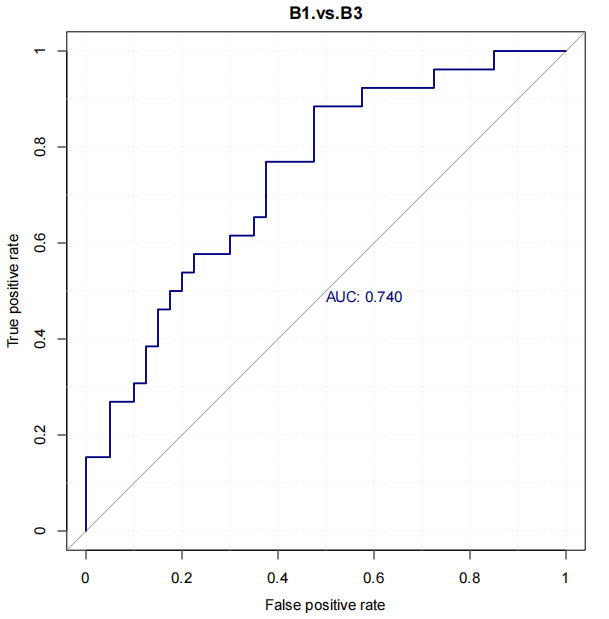


Figure S93: The Discriminatory Ability of Differential Metabolite 1071 between Group B1 and Group B3 under the Positive Ion Mode


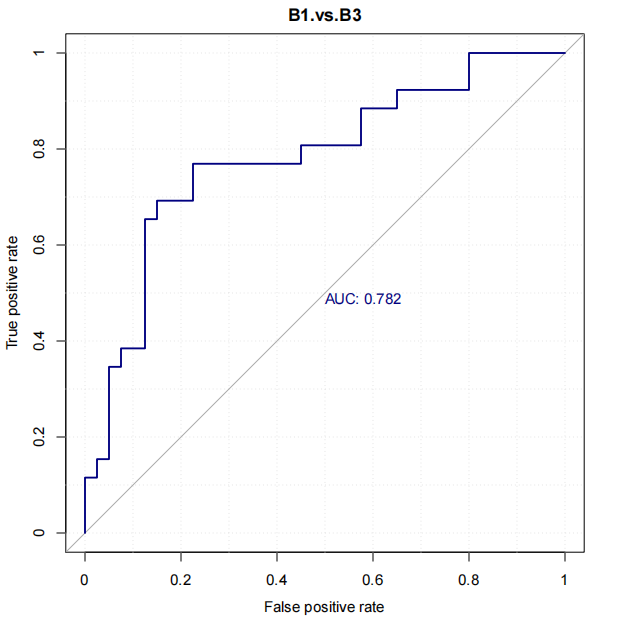


Figure S94: The Discriminatory Ability of Differential Metabolite 4526 between Group B1 and Group B3 under the Positive Ion Mode


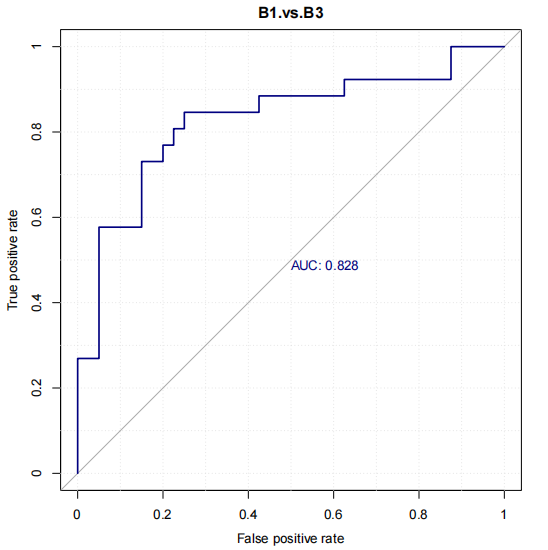


Figure S95: The Discriminatory Ability of Differential Metabolite 5557 between Group B1 and Group B3 under the Positive Ion Mode


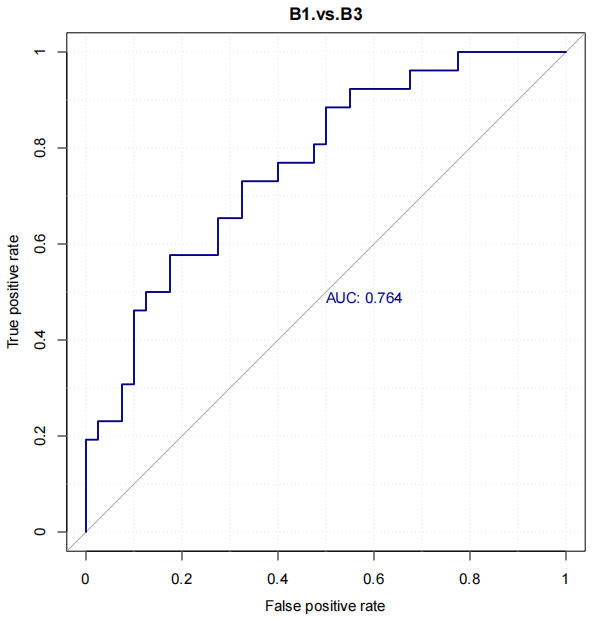


Figure S96: The Discriminatory Ability of Differential Metabolite 6181 between Group B1 and Group B3 under the Positive Ion Mode


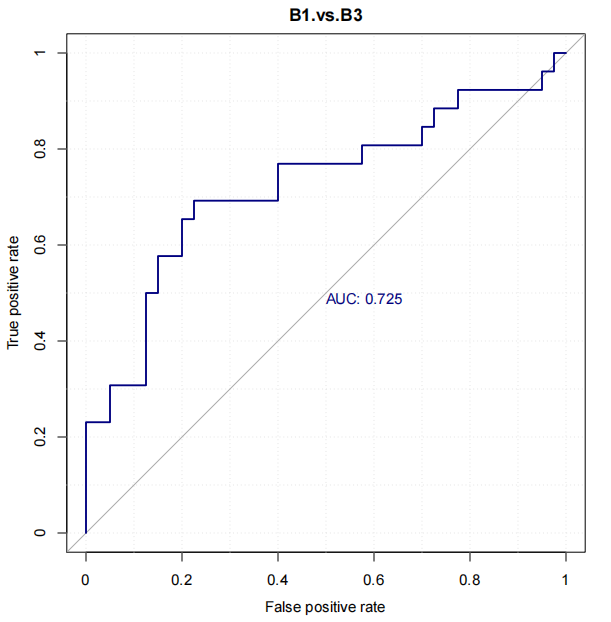


Figure S97: The Discriminatory Ability of Differential Metabolite 8069 between Group B1 and Group B3 under the Positive Ion Mode


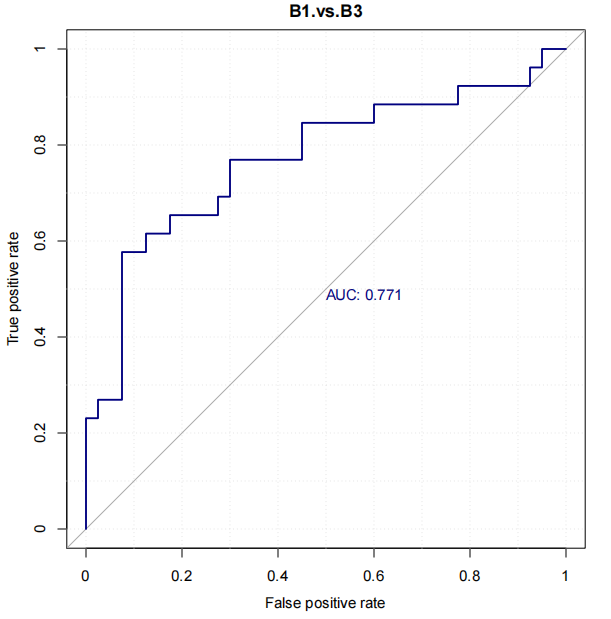


Figure S98: The Discriminatory Ability of Differential Metabolite 10993 between Group B1 and Group B3 under the Positive Ion Mode


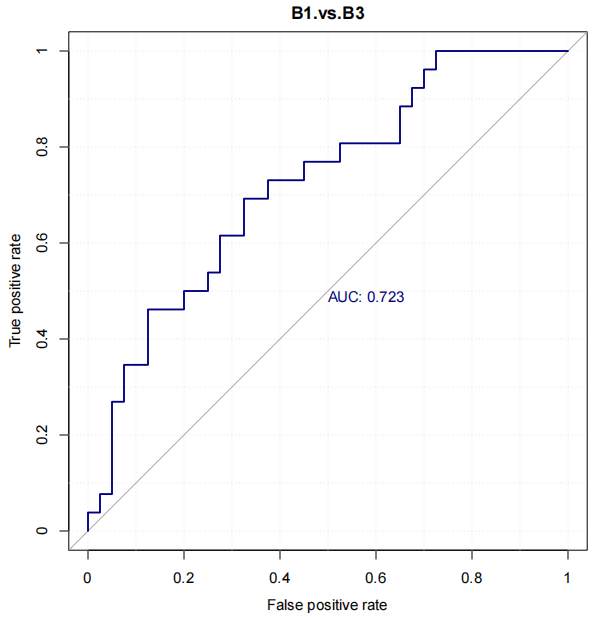


Figure S99: The Discriminatory Ability of Differential Metabolite 11079 between Group B1 and Group B3 under the Positive Ion Mode


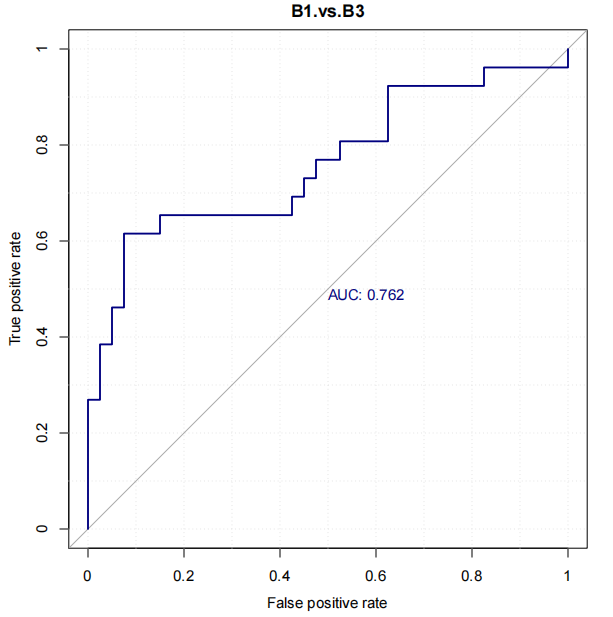


Figure S100: The Discriminatory Ability of Differential Metabolite 12548 between Group B1 and Group B3 under the Positive Ion Mode

Figure S101: The Discriminatory Ability of Differential Metabolite 13296 between Group B1 and Group B3 under the Positive Ion Mode

Figure S102: The Discriminatory Ability of Differential Metabolite 13725 between Group B1 and Group B3 under the Positive Ion Mode

Figure S103: The Discriminatory Ability of Differential Metabolite 14189 between Group B1 and Group B3 under the Positive Ion Mode

Figure S104: The Discriminatory Ability of Differential Metabolite 14249 between Group B1 and Group B3 under the Positive Ion Mode

Figure S105: The Discriminatory Ability of Differential Metabolite 14266 between Group B1 and Group B3 under the Positive Ion Mode

Figure S106: The Discriminatory Ability of Differential Metabolite 14626 between Group B1 and Group B3 under the Positive Ion Mode

Figure S107: The Discriminatory Ability of Differential Metabolite 15348 between Group B1 and Group B3 under the Positive Ion Mode

Figure S108: The Discriminatory Ability of Differential Metabolite 16962 between Group B1 and Group B3 under the Positive Ion Mode

Figure S109: The Discriminatory Ability of Differential Metabolite 20692 between Group B1 and Group B3 under the Positive Ion Mode

Figure S110: The Discriminatory Ability of Differential Metabolite 22455 between Group B1 and Group B3 under the Positive Ion Mode

Figure S111: The Discriminatory Ability of Differential Metabolite 24180 between Group B1 and Group B3 under the Positive Ion Mode

Figure S112: The Discriminatory Ability of Differential Metabolite 24534 between Group B1 and Group B3 under the Positive Ion Mode

Figure S113: The Discriminatory Ability of Differential Metabolite 25308 between Group B1 and Group B3 under the Positive Ion Mode

Figure S114: The Discriminatory Ability of Differential Metabolite 27256 between Group B1 and Group B3 under the Positive Ion Mode

Figure S115: The Discriminatory Ability of Differential Metabolite 51 between Group B2 and Group B3 under the Negative Ion Mode

Figure S116: The Discriminatory Ability of Differential Metabolite 133 between Group B2 and Group B3 under the Negative Ion Mode

Figure S117: The Discriminatory Ability of Differential Metabolite 443 between Group B2 and Group B3 under the Negative Ion Mode

Figure S118: The Discriminatory Ability of Differential Metabolite 659 between Group B2 and Group B3 under the Negative Ion Mode

Figure S119: The Discriminatory Ability of Differential Metabolite 910 between Group B2 and Group B3 under the Negative Ion Mode

Figure S120: The Discriminatory Ability of Differential Metabolite 974 between Group B2 and Group B3 under the Negative Ion Mode

Figure S121: The Discriminatory Ability of Differential Metabolite 1450 between Group B2 and Group B3 under the Negative Ion Mode

Figure S122: The Discriminatory Ability of Differential Metabolite 1457 between Group B2 and Group B3 under the Negative Ion Mode

Figure S123: The Discriminatory Ability of Differential Metabolite 1470 between Group B2 and Group B3 under the Negative Ion Mode

Figure S124: The Discriminatory Ability of Differential Metabolite 1522 between Group B2 and Group B3 under the Negative Ion Mode

Figure S125: The Discriminatory Ability of Differential Metabolite 1593 between Group B2 and Group B3 under the Negative Ion Mode

Figure S126: The Discriminatory Ability of Differential Metabolite 1741 between Group B2 and Group B3 under the Negative Ion Mode

Figure S127: The Discriminatory Ability of Differential Metabolite 1754 between Group B2 and Group B3 under the Negative Ion Mode

Figure S128: The Discriminatory Ability of Differential Metabolite 1757 between Group B2 and Group B3 under the Negative Ion Mode

Figure S129: The Discriminatory Ability of Differential Metabolite 1984 between Group B2 and Group B3 under the Negative Ion Mode

Figure S130: The Discriminatory Ability of Differential Metabolite 1988 between Group B2 and Group B3 under the Negative Ion Mode

Figure S131: The Discriminatory Ability of Differential Metabolite 2054 between Group B2 and Group B3 under the Negative Ion Mode

Figure S132: The Discriminatory Ability of Differential Metabolite 2430 between Group B2 and Group B3 under the Negative Ion Mode

Figure S133: The Discriminatory Ability of Differential Metabolite 2620 between Group B2 and Group B3 under the Negative Ion Mode

Figure S134: The Discriminatory Ability of Differential Metabolite 2765 between Group B2 and Group B3 under the Negative Ion Mode

Figure S135: The Discriminatory Ability of Differential Metabolite 2826 between Group B2 and Group B3 under the Negative Ion Mode

Figure S136: The Discriminatory Ability of Differential Metabolite 2843 between Group B2 and Group B3 under the Negative Ion Mode

Figure S137: The Discriminatory Ability of Differential Metabolite 4051 between Group B2 and Group B3 under the Negative Ion Mode

Figure S138: The Discriminatory Ability of Differential Metabolite 4371 between Group B2 and Group B3 under the Negative Ion Mode

Figure S139: The Discriminatory Ability of Differential Metabolite 4935 between Group B2 and Group B3 under the Negative Ion Mode

Figure S140: The Discriminatory Ability of Differential Metabolite 5124 between Group B2 and Group B3 under the Negative Ion Mode

Figure S141: The Discriminatory Ability of Differential Metabolite 7348 between Group B2 and Group B3 under the Negative Ion Mode

Figure S142: The Discriminatory Ability of Differential Metabolite 7485 between Group B2 and Group B3 under the Negative Ion Mode

Figure S143: The Discriminatory Ability of Differential Metabolite 7670 between Group B2 and Group B3 under the Negative Ion Mode

Figure S144: The Discriminatory Ability of Differential Metabolite 10207 between Group B2 and Group B3 under the Negative Ion Mode

Figure S145: The Discriminatory Ability of Differential Metabolite 6 between Group B2 and Group B3 under the Positive Ion Mode

Figure S146: The Discriminatory Ability of Differential Metabolite 16 between Group B2 and Group B3 under the Positive Ion Mode

Figure S147: The Discriminatory Ability of Differential Metabolite 98 between Group B2 and Group B3 under the Positive Ion Mode

Figure S148: The Discriminatory Ability of Differential Metabolite 285 between Group B2 and Group B3 under the Positive Ion Mode

Figure S149: The Discriminatory Ability of Differential Metabolite 378 between Group B2 and Group B3 under the Positive Ion Mode

Figure S150: The Discriminatory Ability of Differential Metabolite 460 between Group B2 and Group B3 under the Positive Ion Mode

Figure S151: The Discriminatory Ability of Differential Metabolite 526 between Group B2 and Group B3 under the Positive Ion Mode

Figure S152: The Discriminatory Ability of Differential Metabolite 649 between Group B2 and Group B3 under the Positive Ion Mode

Figure S153: The Discriminatory Ability of Differential Metabolite 661 between Group B2 and Group B3 under the Positive Ion Mode

Figure S154: The Discriminatory Ability of Differential Metabolite 779 between Group B2 and Group B3 under the Positive Ion Mode

Figure S155: The Discriminatory Ability of Differential Metabolite 823 between Group B2 and Group B3 under the Positive Ion Mode

Figure S156: The Discriminatory Ability of Differential Metabolite 1044 between Group B2 and Group B3 under the Positive Ion Mode

Figure S157: The Discriminatory Ability of Differential Metabolite 1063 between Group B2 and Group B3 under the Positive Ion Mode

Figure S158: The Discriminatory Ability of Differential Metabolite 1071 between Group B2 and Group B3 under the Positive Ion Mode

Figure S159: The Discriminatory Ability of Differential Metabolite 1163 between Group B2 and Group B3 under the Positive Ion Mode

Figure S160: The Discriminatory Ability of Differential Metabolite 1204 between Group B2 and Group B3 under the Positive Ion Mode

Figure S161: The Discriminatory Ability of Differential Metabolite 1485 between Group B2 and Group B3 under the Positive Ion Mode

Figure S162: The Discriminatory Ability of Differential Metabolite 1945 between Group B2 and Group B3 under the Positive Ion Mode

Figure S163: The Discriminatory Ability of Differential Metabolite 2241 between Group B2 and Group B3 under the Positive Ion Mode

Figure S164: The Discriminatory Ability of Differential Metabolite 2879 between Group B2 and Group B3 under the Positive Ion Mode

Figure S165: The Discriminatory Ability of Differential Metabolite 2948 between Group B2 and Group B3 under the Positive Ion Mode

Figure S166: The Discriminatory Ability of Differential Metabolite 3323 between Group B2 and Group B3 under the Positive Ion Mode

Figure S167: The Discriminatory Ability of Differential Metabolite 3428 between Group B2 and Group B3 under the Positive Ion Mode

Figure S168: The Discriminatory Ability of Differential Metabolite 3456 between Group B2 and Group B3 under the Positive Ion Mode

Figure S169: The Discriminatory Ability of Differential Metabolite 3640 between Group B2 and Group B3 under the Positive Ion Mode

Figure S170: The Discriminatory Ability of Differential Metabolite 3820 between Group B2 and Group B3 under the Positive Ion Mode

Figure S171: The Discriminatory Ability of Differential Metabolite 3867 between Group B2 and Group B3 under the Positive Ion Mode

Figure S172: The Discriminatory Ability of Differential Metabolite 3881 between Group B2 and Group B3 under the Positive Ion Mode

Figure S173: The Discriminatory Ability of Differential Metabolite 4219 between Group B2 and Group B3 under the Positive Ion Mode

Figure S174: The Discriminatory Ability of Differential Metabolite 4276 between Group B2 and Group B3 under the Positive Ion Mode

Figure S175: The Discriminatory Ability of Differential Metabolite 4502 between Group B2 and Group B3 under the Positive Ion Mode

Figure S176: The Discriminatory Ability of Differential Metabolite 4526 between Group B2 and Group B3 under the Positive Ion Mode

Figure S177: The Discriminatory Ability of Differential Metabolite 4596 between Group B2 and Group B3 under the Positive Ion Mode

Figure S178: The Discriminatory Ability of Differential Metabolite 4672 between Group B2 and Group B3 under the Positive Ion Mode

Figure S179: The Discriminatory Ability of Differential Metabolite 4834 between Group B2 and Group B3 under the Positive Ion Mode

Figure S180: The Discriminatory Ability of Differential Metabolite 4880 between Group B2 and Group B3 under the Positive Ion Mode

Figure S181: The Discriminatory Ability of Differential Metabolite 5557 between Group B2 and Group B3 under the Positive Ion Mode

Figure S182: The Discriminatory Ability of Differential Metabolite 5636 between Group B2 and Group B3 under the Positive Ion Mode

Figure S183: The Discriminatory Ability of Differential Metabolite 5657 between Group B2 and Group B3 under the Positive Ion Mode

Figure S184: The Discriminatory Ability of Differential Metabolite 6181 between Group B2 and Group B3 under the Positive Ion Mode

Figure S185: The Discriminatory Ability of Differential Metabolite 6262 between Group B2 and Group B3 under the Positive Ion Mode

Figure S186: The Discriminatory Ability of Differential Metabolite 6397 between Group B2 and Group B3 under the Positive Ion Mode

Figure S187: The Discriminatory Ability of Differential Metabolite 6424 between Group B2 and Group B3 under the Positive Ion Mode

Figure S188: The Discriminatory Ability of Differential Metabolite 7242 between Group B2 and Group B3 under the Positive Ion Mode

Figure S189: The Discriminatory Ability of Differential Metabolite 7452 between Group B2 and Group B3 under the Positive Ion Mode

Figure S190: The Discriminatory Ability of Differential Metabolite 8025 between Group B2 and Group B3 under the Positive Ion Mode

Figure S191: The Discriminatory Ability of Differential Metabolite 8069 between Group B2 and Group B3 under the Positive Ion Mode

Figure S192: The Discriminatory Ability of Differential Metabolite 8470 between Group B2 and Group B3 under the Positive Ion Mode

Figure S193: The Discriminatory Ability of Differential Metabolite 8910 between Group B2 and Group B3 under the Positive Ion Mode

Figure S194: The Discriminatory Ability of Differential Metabolite 9158 between Group B2 and Group B3 under the Positive Ion Mode

Figure S195: The Discriminatory Ability of Differential Metabolite 9530 between Group B2 and Group B3 under the Positive Ion Mode

Figure S196: The Discriminatory Ability of Differential Metabolite 9678 between Group B2 and Group B3 under the Positive Ion Mode

Figure S197: The Discriminatory Ability of Differential Metabolite 9703 between Group B2 and Group B3 under the Positive Ion Mode

Figure S198: The Discriminatory Ability of Differential Metabolite 9778 between Group B2 and Group B3 under the Positive Ion Mode

Figure S199: The Discriminatory Ability of Differential Metabolite 9857 between Group B2 and Group B3 under the Positive Ion Mode

Figure S200: The Discriminatory Ability of Differential Metabolite 10829 between Group B2 and Group B3 under the Positive Ion Mode

Figure S201: The Discriminatory Ability of Differential Metabolite 10993 between Group B2 and Group B3 under the Positive Ion Mode

Figure S202: The Discriminatory Ability of Differential Metabolite 11079 between Group B2 and Group B3 under the Positive Ion Mode

Figure S203: The Discriminatory Ability of Differential Metabolite 12299 between Group B2 and Group B3 under the Positive Ion Mode

Figure S204: The Discriminatory Ability of Differential Metabolite 12350 between Group B2 and Group B3 under the Positive Ion Mode

Figure S205: The Discriminatory Ability of Differential Metabolite 13687 between Group B2 and Group B3 under the Positive Ion Mode

Figure S206: The Discriminatory Ability of Differential Metabolite 13725 between Group B2 and Group B3 under the Positive Ion Mode

Figure S207: The Discriminatory Ability of Differential Metabolite 14189 between Group B2 and Group B3 under the Positive Ion Mode

Figure S208: The Discriminatory Ability of Differential Metabolite 14626 between Group B2 and Group B3 under the Positive Ion Mode

Figure S209: The Discriminatory Ability of Differential Metabolite 14679 between Group B2 and Group B3 under the Positive Ion Mode

Figure S210: The Discriminatory Ability of Differential Metabolite 15348 between Group B2 and Group B3 under the Positive Ion Mode

Figure S211: The Discriminatory Ability of Differential Metabolite 15938 between Group B2 and Group B3 under the Positive Ion Mode

Figure S212: The Discriminatory Ability of Differential Metabolite 16156 between Group B2 and Group B3 under the Positive Ion Mode

Figure S213: The Discriminatory Ability of Differential Metabolite 16384 between Group B2 and Group B3 under the Positive Ion Mode

Figure S214: The Discriminatory Ability of Differential Metabolite 16684 between Group B2 and Group B3 under the Positive Ion Mode

Figure S215: The Discriminatory Ability of Differential Metabolite 16962 between Group B2 and Group B3 under the Positive Ion Mode

Figure S216: The Discriminatory Ability of Differential Metabolite 17785 between Group B2 and Group B3 under the Positive Ion Mode

Figure S217: The Discriminatory Ability of Differential Metabolite 18426 between Group B2 and Group B3 under the Positive Ion Mode

Figure S218: The Discriminatory Ability of Differential Metabolite 18429 between Group B2 and Group B3 under the Positive Ion Mode

Figure S219: The Discriminatory Ability of Differential Metabolite 18576 between Group B2 and Group B3 under the Positive Ion Mode

Figure S220: The Discriminatory Ability of Differential Metabolite 19816 between Group B2 and Group B3 under the Positive Ion Mode

Figure S221: The Discriminatory Ability of Differential Metabolite 19917 between Group B2 and Group B3 under the Positive Ion Mode

Figure S222: The Discriminatory Ability of Differential Metabolite 20155 between Group B2 and Group B3 under the Positive Ion Mode

Figure S223: The Discriminatory Ability of Differential Metabolite 20355 between Group B2 and Group B3 under the Positive Ion Mode

Figure S224: The Discriminatory Ability of Differential Metabolite 20560 between Group B2 and Group B3 under the Positive Ion Mode

Figure S225: The Discriminatory Ability of Differential Metabolite 20761 between Group B2 and Group B3 under the Positive Ion Mode

Figure S226: The Discriminatory Ability of Differential Metabolite 21438 between Group B2 and Group B3 under the Positive Ion Mode

Figure S227: The Discriminatory Ability of Differential Metabolite 22048 between Group B2 and Group B3 under the Positive Ion Mode

Figure S228: The Discriminatory Ability of Differential Metabolite 22455 between Group B2 and Group B3 under the Positive Ion Mode

Figure S229: The Discriminatory Ability of Differential Metabolite 22850 between Group B2 and Group B3 under the Positive Ion Mode

Figure S230: The Discriminatory Ability of Differential Metabolite 22920 between Group B2 and Group B3 under the Positive Ion Mode

Figure S231: The Discriminatory Ability of Differential Metabolite 22940 between Group B2 and Group B3 under the Positive Ion Mode

Figure S232: The Discriminatory Ability of Differential Metabolite 23780 between Group B2 and Group B3 under the Positive Ion Mode

Figure S233: The Discriminatory Ability of Differential Metabolite 24180 between Group B2 and Group B3 under the Positive Ion Mode

Figure S234: The Discriminatory Ability of Differential Metabolite 24534 between Group B2 and Group B3 under the Positive Ion Mode

Figure S235: The Discriminatory Ability of Differential Metabolite 26429 between Group B2 and Group B3 under the Positive Ion Mode

Figure S236: The Discriminatory Ability of Differential Metabolite 27480 between Group B2 and Group B3 under the Positive Ion Mode

Figure S237: The Discriminatory Ability of Differential Metabolite 27844 between Group B2 and Group B3 under the Positive Ion Mode
